# Genetic control of functional traits related to photosynthesis and water use efficiency in *Pinus pinaster* Ait. drought response: integration of genome annotation, allele association and QTL detection for candidate gene identification

**DOI:** 10.1186/1471-2164-15-464

**Published:** 2014-06-12

**Authors:** Marina de Miguel, José-Antonio Cabezas, Nuria de María, David Sánchez-Gómez, María-Ángeles Guevara, María-Dolores Vélez, Enrique Sáez-Laguna, Luis-Manuel Díaz, Jose-Antonio Mancha, María-Carmen Barbero, Carmen Collada, Carmen Díaz-Sala, Ismael Aranda, María-Teresa Cervera

**Affiliations:** Departamento de Ecología y Genética Forestal, INIA-CIFOR., Ctra, de La Coruña Km 7.5, 28040 Madrid, Spain; Unidad Mixta de Genómica y Ecofisiología Forestal, INIA/UPM, Madrid, Spain; ETSIM, Departamento de Biotecnología, Ciudad Universitaria, s/n, 28040 Madrid, Spain; Departamento de Ciencias de la Vida, Universidad de Alcalá, Ctra. de Barcelona Km 33.6, 28871 Alcalá de Henares, Madrid, Spain

**Keywords:** Candidate gene, Drought, Genome annotation, Photochemistry, Photosynthesis, *Pinus pinaster*, QTL, Water use efficiency

## Abstract

**Background:**

Understanding molecular mechanisms that control photosynthesis and water use efficiency in response to drought is crucial for plant species from dry areas. This study aimed to identify QTL for these traits in a Mediterranean conifer and tested their stability under drought.

**Results:**

High density linkage maps for *Pinus pinaster* were used in the detection of QTL for photosynthesis and water use efficiency at three water irrigation regimes. A total of 28 significant and 27 suggestive QTL were found. QTL detected for photochemical traits accounted for the higher percentage of phenotypic variance. Functional annotation of genes within the QTL suggested 58 candidate genes for the analyzed traits. Allele association analysis in selected candidate genes showed three SNPs located in a MYB transcription factor that were significantly associated with efficiency of energy capture by open PSII reaction centers and specific leaf area.

**Conclusions:**

The integration of QTL mapping of functional traits, genome annotation and allele association yielded several candidate genes involved with molecular control of photosynthesis and water use efficiency in response to drought in a conifer species. The results obtained highlight the importance of maintaining the integrity of the photochemical machinery in *P. pinaster* drought response.

**Electronic supplementary material:**

The online version of this article (doi:10.1186/1471-2164-15-464) contains supplementary material, which is available to authorized users.

## Background

Drought resistance is crucial for growth and survival of species living in water scarce environments [1]. Unraveling the molecular mechanisms that control functional traits, such as photosynthesis and water use efficiency in response to drought, is especially relevant in view of its implication in survival, growth and biomass production. However, carbon uptake in response to drought is a complex process with many mechanisms acting in coordination in final CO_2_ fixation [2]. From stomatal and mesophyll resistances to diffusion of CO_2_ to biochemical processes within chloroplast, complex mechanisms are involved in net carbon fixation [2–5]. The functional bases that control carbon uptake under water stress have been largely studied [6, 7], but less information is available about its genetic regulation.

Complex functional trait dissection can be achieved through two approaches: association studies and QTL (Quantitative Trait Loci) mapping [8]. The resolution power of association studies is higher than QTL mapping [9]. However, the rapid decay in linkage disequilibrium of conifers [10] makes the development of genome wide association studies in these species laborious and advocates in favor of candidate gene approaches [11].

In the past, identification of candidate genes underlying QTL was difficult due to the use of anonymous markers and limited sequences with functional information, and thus restricted the approach to model plant species [12]. Nowadays, gene-based markers are easily developed and much more functional information is available for a wide range of organisms [13–18], allowing to integrate functional annotation with QTL studies [19]. Moreover, the development and application of high throughput genotyping technologies have allowed the construction of dense genetic maps [20–27], http://dendrome.ucdavis.edu/cmap/]. The use of highly saturated genetic maps allows to narrow down the position of loci involved in the genetic control of the targeted trait and the combination of high density gene based maps with functional annotation allows to identify positional candidate genes for these QTL [19, 28]. Suggested candidate genes are suitable for association studies that can validate marker-trait associations [29]. Therefore, identification of positional candidate genes within QTL confidence intervals, some of them with known function in other species, could be considered as a preliminary step that contributes to the detection of genes underlying traits of interest [30]. Additionally, QTL mapping allows the evaluation of the genetic basis for potential adaptation in natural populations [31, 32] and to extend the understanding of relationships between different morpho-functional traits [33]. The identification of the main QTL involved in drought response could be a first step to develop marker assisted selection (MAS) strategies for these traits [11].

Consequently, the detection of QTL involved in photosynthesis and water use efficiency in the context of drought response is a first attempt to understand the genetic basis regulating the expression of these traits. QTL studies on functional drought response have been largely implemented for non-forest model species [34–39]. Some of these QTL studies in crop species have recently identified genomic regions controlling photochemistry of carbon uptake [40, 41]. Breeding programs implemented in crops have reported yield improvement associated with increased photosynthesis [42]. However, fewer QTL analyses on functional drought response of forest tree species have been performed [11, 43–46] and to our knowledge none of them has focused on the photochemical machinery.

QTL studies involve development of a segregant progeny for target traits, phenotypic and molecular characterization of the progeny and construction of genetic maps [47]. The power to resolve the location of a QTL is related to the size of the studied population and the mapping coverage [48]. Additionally, forest tree species are characterized by long generation times which hinder development of backcross or three-generation pedigrees by controlled crosses. On the other hand, replication of each genotype is needed for a reliable phenotypic evaluation [49], especially when working with physiological parameters that are extremely sensible to environmental conditions [41, 50, 51].

Mediterranean species are particularly threatened by drought [52–54], especially in the context of climatic change predictions [55]. *Pinus pinaster* Ait. is an important conifer in Mediterranean region with a high ecological and socio-economical value [56–58]. Although *P. pinaster* shows evidence of drought adaptation [59, 60], recurrent or severe drought periods can limit its growth [61, 62]. Understanding the molecular basis of drought tolerance is of high importance for a suitable management of the available genetic resources of *P. pinaster* in conservation, afforestation or breeding programs. QTL and association studies of drought tolerance traits have been developed in several tree species, such as *P. taeda*[23, 63, 64], *Populus* sp. [19, 65, 66] or *Quercus robur*[43, 67]. Several QTL and association studies in *P. pinaster* have analyzed the molecular basis of different processes related to growth or wood quality traits [68–73], terpenes [74] and serotiny [75]. However, to date only association studies based on a few potential candidate genes [59, 60] and one QTL study have analyzed the molecular basis of drought tolerance in *P. pinaster*[44].

The main objective of this work was to unravel the genetic basis of different functional parameters related to carbon uptake and water use efficiency in response to drought for *P. pinaster.* For this purpose a QTL analysis using vegetatively propagated genotypes in order to improve the reliability of phenotypic estimates was designed. Several specific objectives were outlined: 1) construction of dense gene-based linkage maps with functional information; 2) identification of genomic regions underlying photosynthesis and water use efficiency in response to drought through QTL analysis; and 3) identification of a set of promising candidate genes in targeted genomic regions that may be involved in the genetic regulation of photosynthesis and water use efficiency in response to drought.

## Methods

### Plant material, experimental setup and phenotypic evaluation

Plant material, experimental setup and phenotypic evaluation are explained in detail in de Miguel et al. [76]. Briefly, 162 seedlings from a F1 full-sib family of *P. pinaster* obtained from a controlled cross between a male parent (Oria6) from Oria, a natural population from South-East Spain (37° 31 ’N 2° 21 ’W) and a female parent (Gal1056) from a breeding program established in Pontevedra, North-West Spain (42° 10 ’N 8° 30 ’W), were vegetatively replicated and established in an incomplete block design in a greenhouse at Instituto Nacional de Investigación y Tecnología Agraria y Alimentaria (INIA). Phenotypic evaluations were conducted on the 103 clones for which at least three ramets were obtained. For phenotypic characterization three time-points of measurement were carried out starting in October 2009. During the 1^st^ time-point, plants were watered close to full holding capacity. Then, watering was withdrawn and during the 2^nd^ time-point of measurement plants were left 7 days without watering. The final third batch of measurements was carried out after plants have been 14 days without watering. Net photosynthetic rate (A_n_, μmol CO_2_m^-2^ s^-1^), stomatal conductance to water vapour (g_sw_, molH_2_Om^-2^ s^-1^), intrinsic water use efficiency (WUE_i_, μmol CO_2_ molH_2_O^-1^), specific leaf area (SLA, m^2^Kg^-1^), maximum efficiency of photosystem II under light conditions (F_v_’F_m_’) and quantum yield (Φ_PSII_) were measured for all plants. Chlorophyll fluorescence parameters were measured following the procedure described in Cano et al. [6].

In the 1^st^ time-point of measurement four adult needles were collected for each plant, dried and ground into a fine homogeneous powder. Carbon isotope composition was measured with a PDZ Europa ANCA-GSL elemental analyzer interfaced to a PDZ Europa 20–20 continuous flow isotope ratio mass spectrometer (Sercon Ltd., Cheshire, UK) at Stable Isotope Facility UC Davis, California, USA. The isotopic composition of ^13^C (‰) was expressed as [77]:


Where R_s_ and R_b_ refer to the ^13^C/^12^C ratio in the sample and in the Pee Dee Belemnite standard, respectively.

Broad-sense heritability estimates and genetic correlations were calculated for the analyzed traits according to de Miguel et al. [76].

### DNA extraction and marker genotyping

The mapping progeny was genotyped with nuclear microsatellites (single sequence repeats, nSSR), selective amplification of microsatellite polymorphic loci (SAMPL) and single nucleotide polymorphism (SNP) markers. Different DNA extraction methods were used in needles: a modified protocol from Dellaporta et al. [78] for nSSRs, SAMPLs and SNP array D (detailed below); the commercial kit *Invisorb DNA plants HTS 96 kit* (Invitek GmbH, Berlin, Germany) for SNP arrays A and C (detailed below) and the commercial kit *DNeasy Plant mini kit* (Qiagen, Düsseldorf, Germany) for SNP array B (detailed below).

A total of twenty nine primer pairs designed for amplification of nSSR loci in *P. pinaster* and *P. taeda*[79, 80] were tested for their segregations in the mapping population and both progenitors and six progeny individuals were genotyped. The whole mapping progeny was then genotyped only for polymorphic microsatellite loci. PCRs were performed in 10 μl containing 10 ng of DNA, 1x PCR reaction buffer (Invitrogen, Grand Island, NY, USA), 250 μM of each dNTP (Invitrogen, Grand Island, NY, USA), 0.25 U Taq polymerase (Invitrogen, Grand Island, NY, USA), 4 mM MgCl_2_ (Invitrogen, Grand Island, NY, USA) except for A6D12 where 2 mM MgCl_2_ was used, 0.2 μM of forward primer and 0.2 μM of reverse primer labeled on its 5’ end with IRD800. The PCR profile used was 94°C 4 min, 2 cycles of 94°C 45 s, 60°C 45 s, 72°C 45 s, 18 touchdown cycles of 94°C 45 s, 59.5°C 45 s (-0.5°C/cycle), 72°C 45 s, 20 cycles 94°C 30 s, 50°C 30 s, 72°C 45 s and final extension at 72°C 5 min. PCR reactions were carried out with a Perkin-Elmer GenAmp 9700 thermal cycler (Perkin Elmer Inc., Waltham, Massachusetts, USA). Amplified products were separated on denaturing gels containing 6% (w/v) acrylamide/bisacrylamide (19:3), 7 M urea and 1 x TBE and visualized in a 4300 DNA Analyzer (LI-COR Biosciences, Lincoln, NE, USA). Fragments were scored visually as codominant markers.

SAMPL genotyping was performed as in de Miguel et al. [81] with several modifications. Preamplification was carried out using *Eco*RI + A / *Mse*I + C primer combination. In order to identify the most informative selective primer combinations (those with a higher number of informative polymorphic fragments) different primer combinations were tested using DNA from the progenitors and 6 offspring. A total of five CATA/*Eco*RI and three GATA/*Eco*RI primer combinations were used for the selective amplification. The whole mapping progeny was then genotyped for the eight selected SAMPL primer combinations. Primers CATA and GATA were IRDye 700 and IRDye 800 5’end labeled, respectively. Samples were loaded into denaturing gels containing 16% (w/v) Long Ranger® 50% (w/v) Gel Solution (Lonza, Basel, Switzerland), 7 M urea and 1 x TBE. Fragment detection was carried out on a 4300 DNA Analyzer (LI-COR Biosciences, Lincoln, NE, USA). Each gel was visually scored twice independently by two different people.

In this study, four SNP genotyping assays were used, three of which were Golden Gate assays (Illumina Inc., San Diego, CA, USA): SNP arrays A and C, which were two different 1,536 BeadArray™ experiments; and SNP array B, which was a 384 BeadXpress®. The SNP array D was a 12 K Infinium assay (Illumina Inc., San Diego, CA, USA). SNP arrays B and C were used to genotype the whole mapping progeny, whereas A and D could be used only on 83 and 70 progeny individuals, respectively. SNP array B was developed including many of the SNPs targeted in array A [82] and 14 additional SNPs from candidate genes for drought resistance [59] in order to complete the information for a set of genes of special interest (see de Miguel et al. [81] for further details). When the same SNP was successfully genotyped in both assays only the data of SNP array B was used because of the higher number of individuals genotyped in this assay. SNP array C was designed using a *P. pinaster* gene catalog obtained from 454 sequencing of cDNA libraries constructed with different tissues from 9 siblings of the mapping progeny submitted to different growing conditions (*i.e.* drought stress versus control plants; E Sáez-Laguna et al., unpublished). The genotyping of SNP array C was developed at CNIO, Madrid, Spain. Finally, SNP array D contained 10,593 SNPs identified from unigene set “PineContig_v2” of *P. pinaster*[20]. The four genotyping assays were carried out according to the manufacturer’s instructions (Illumina Inc., San Diego, CA, USA) and SNPs clusters revised manually with Illumina Genome Studio v.1.9.4 software with a GenCall score cutoff of 0.15. SNP clusters were modified manually to refine cluster positions when necessary. For the SNP array D (12 K Infinium) SNPs with Gen-Train values lower than 0.25 were discarded, with values between 0.25 and 0.5 were manually scored and with values higher than 0.5 were automatically scored.

### Construction of dense linkage maps

For the construction of two genetic maps, one for each progenitor (Gal1056 and Oria6), the “two-way-pseudo-testcross” mapping strategy was applied [83]. The consensus map for the cross, combining markers informative for both parents, was also developed (GxO). Linkage analyses and map estimations were performed using the regression mapping algorithm implemented in the software JoinMap® v4.1 [84] with the CP population type and using a recombination fraction < 0.35 and a LOD > 3 as mapping parameters. Map distances were calculated using Kosambi mapping function [85]. For map building a goodness-of-fit jump threshold of 5 was established. JoinMap suggests three genetic maps with increasing number of markers (map1, map2 and map3). In map2, new markers were added because more pair wise data were available but statistical support is the same as in map1. In map3, the remaining loci were added by increasing the goodness-of fit jump threshold. In these cases map2 was kept for further analyses. Mean χ^2^ contribution to the goodness of fit and number of double recombinants were inspected in order to remove not reliably positioned markers from the estimated maps. When a pair of markers was considered identical based on the lack of recombination between them, only one of the markers was selected for mapping (see Additional file 1). Segregation ratios were tested using χ^2^ test (p ≤ 0.01) after Bonferroni correction. Framework maps for Gal1056, Oria6 and GxO were also built. For this purpose, only the most informative markers with very reliable positions and inter marker distance of circa 10 cM were kept. Total genome length was calculated as the sum of all mapped marker intervals. Estimated genome length was determined from the partial linkage data according to Hulbert et al. [86] modified by Chakravarti et al. [87] (Method 3). To estimate genome length using framework maps, a minimum LOD score of three was chosen. Observed map coverage was calculated as the ratio of total genome length to estimated genome length. To estimate the number of different mapped genes a BlastN was performed between gene sequences contained in the different SNP genotyping arrays. Sequences with a percentage of identity higher than 98% were considered the same gene. To test whether the mapped genes were evenly distributed between linkage groups χ^2^ tests (p < 0.05) were performed by comparing observed and estimated numbers of genes per linkage group (LG). The expected number of genes for each LG was obtained by multiplying the ratio size of LG to total genome length by the total number of mapped genes. Linkage maps were compared with previously developed *P. pinaster* maps [20, 81, 82] based on common SNPs and SSRs.

### QTL mapping

In order to avoid errors in marker order that may have some impact on the precision and accuracy of QTL placement, QTL analyses were performed using the framework linkage maps. QTL detection was carried out using the regression algorithm implemented in the software MapQTL® v6.0 [88]. Interval mapping was applied followed by multiple QTL mapping (MQM) when more than one QTL was found for a trait. Analyses were performed using a mapping step size of one. The thresholds (95% and 99% confidence) for QTL significance were determined using a chromosome and genome wide permutation test with 10,000 iterations. Support intervals for the detected QTL were estimated based on the observed decrease of LOD value in one and two units. QTL identified with only the 95% significance at chromosome level were considered as suggestive of putative QTL. Each detected QTL received an identification name indicating the measured trait, the time-point of measurement, the linkage group (LG) and the map (“f” and “m” for female and male parents respectively and “i” for consensus map) where the QTL was detected.

### Candidate genes search

Functional annotation for gene based markers of SNP arrays A, B and D were described by Chancerel et al. [20, 82]. Functional annotation for SNP array C was obtained with Blast2GO software [89]. For high-scoring segment pair (HSP) a restrictive E^-20^ e-value was chosen finding in a Blastx search against a set of 88,516 reference proteins from UniProt (http://www.uniprot.org/). In order to update annotation information for the sequences of the four SNP arrays that mapped to the QTL, a second round of annotation was performed using Blast2GO software with a restrictive E^-25^ e-value for HSP, and annotation was completed with InterPro (http://www.ebi.ac.uk/interpro/) and Kyoto Encyclopedia of Genes and Genomes (KEGG, http://www.genome.jp/kegg/) searches. For those genes within the significant QTL confidence intervals (±2 LOD), functional annotations were queried to identify functional relationship between the positional candidate genes and each analyzed trait. In order to cover all mapped genes, QTL confidence intervals in framework maps were extrapolated to maps with all the mapped markers.

Association between phenotypes and alleles at candidate loci was further studied by ANOVA using the traits as dependent variables and the SNP genotypes as factors. Thereof nineteen traits (seven different traits measured at three water irrigation regimes, except δ^13^C measured only at 1^st^ time-point) and 73 SNPs located in 58 identified candidate genes were inspected. False discovery rate (FDR) was calculated using the package *qvalue*. Association analyses were carried out in R version 2.15.2 (R Development Core Team, 2012).

## Results

### Phenotypic evaluation

Descriptive statistics of all analyzed traits are shown in Table 1. Almost all traits showed a close to normal distribution with low levels of skewness and kurtosis. Although normal distribution is an assumption in interval mapping, this method and MQM are quiet robust against deviations from normality [88]. Water stress produced a decrease in mean values for almost all variables except for WUE_i_ and SLA that showed higher and very similar mean values, respectively, for the three time-points of measurement. Coefficients of variation were progressively higher with the imposition of drought stress being g_sw_ the trait that showed the higher coefficient of variation in the 3^rd^ time-point of measurement.

**Table 1:** **Descriptive statistics of measured traits in the F**_**1**_ **full sib family Gal1056xOria6 (n = 103)**

Trait	Time-point	Mean ± SD	Range	CV(%)	Skewness	Kurtosis	p-value
A_n_	1	10.8 ± 1.3	7.9-15.2	12.4	0.65	0.46	0.04
	2	10.1 ± 1.3	6.5-12.9	12.4	-0.21	0.16	0.63
	3	5.9 ± 1.7	1.2-9.9	29.8	-0.18	-0.3	0.67
g_sw_	1	0.21 ± 0.03	0.141-0.301	15.9	0.47	0.09	0.04
	2	0.146 ± 0.03	0.068-0.222	22.6	0.06	-0.44	0.57
	3	0.07 ± 0.03	0.01-0.198	45.7	0.71	1.28	0.02
WUE_i_	1	54.7 ± 9.8	32.5-81.8	17.9	0.7	0.52	0.003
	2	79.9 ± 16.4	41.2-130.1	20.6	0.4	0.21	0.38
	3	100.4 ± 27.8	44.8-179.4	27.7	0.52	0.34	0.05
δ^13^C	1	-29.6 ± 0.64	-31.1/-28	2.2	0.003	-0.09	0.92
SLA	1	7.3 ± 0.7	5.8-10.4	9.6	1.16	3.26	<0.001
	2	6.7 ± 0.6	5.6-8.6	9.4	0.55	-0.005	0.03
	3	6.6 ± 0.6	5.3-8.8	9.5	0.68	0.94	0.02
F_v_'F_m_'	1	0.618 ± 0.03	0.496-0.684	5.3	-0.71	1.16	0.02
	2	0.575 ± 0.04	0.48-0.688	7.5	-0.32	-0.26	0.04
	3	0.468 ± 0.04	0.361-0.591	8.5	0.14	0.17	0.94
Φ_PSII_	1	0.21 ± 0.02	0.157-0.252	9.9	-0.44	0.03	0.09
	2	0.213 ± 0.03	0.153-0.276	11.8	0.15	-0.43	0.63
	3	0.16 ± 0.02	0.096-0.223	15.5	-0.008	-0.05	0.99

A_n_ = net photosynthetic rate (μmol CO_2_m^-2^ s^-1^); g_sw_ = stomatal conductance to water vapour (molH_2_Om^-2^ s^-1^); WUE_i_ = intrinsic water use efficiency (μmol CO_2_ molH_2_O^-1^); δ^13^C = isotopic composition of ^13^C (‰); SLA = specific leaf area (m^2^Kg^-1^); F_v_’F_m_’ = maximum efficiency of PSII under light conditions; Φ_PSII_ = quantum yield. Time-points of measurements correspond with: 1, well watered plants; 2, seven days without irrigation; 3, fourteen days without irrigation. SD stands for standard deviation and CV for coefficient of variation. p-values were obtained from Shapiro- test to check normality.

Phenotypic correlations between the studied traits are presented in Table 2. A_n_ was correlated with g_sw_ and with chlorophyll fluorescence parameters (F_v_’F_m_’ and Φ_PSII_). The magnitude of the correlation coefficients was very similar for the 1^st^ time-point of measurement. However, under drought stress A_n_ showed a higher correlation coefficient with chlorophyll fluorescence parameters than with g_sw_. Besides, A_n_ and chlorophyll fluorescence parameters showed a tight genetic correlation (see Additional file 2). For WUE_i_ and δ^13^C, a significant phenotypic (Table 2) and broad sense genetic correlation (see Additional file 2) was found. Both traits had higher phenotypic correlation coefficients with g_sw_ than with A_n_. SLA was moderately correlated with A_n_, g_sw_, WUE_i_ and δ^13^C for the 1^st^ and 2^nd^ time-points of measurement (Table 2). Broad sense heritability estimates for the analyzed traits are presented in Additional file 3. All of them presented moderate to low values of heritability being the higher estimates for g_sw_, WUE_i_ and δ^13^C.

**Table 2:** **Pearson correlation coefficients and statistical significance for measured traits in the F**_**1**_ **full sib family Gal1056xOria6 (n = 103)**

Time-point	Trait	g _sw_	WUE _i_	δ ^13^ C	SLA	F _v_ ’F _m_ ’	Φ _PSII_
1	A_n_	0.42**	0.36**	n.s	0.23*	0.47**	0.54**
	g_sw_		-0.61**	-0.51**	0.37**	0.24*	n.s
	WUE_i_			0.49**	n.s	n.s	0.37**
	δ^13^C				0.34**	n.s	0.31**
	SLA					0.21*	n.s
	F_v_’F_m_’						0.31**
2	A_n_	0.52**	n.s	-	n.s	0.62**	0.58**
	g_sw_		-0.8**	-	0.29**	0.5**	0.2*
	WUE_i_			-	0.21*	0.24*	n.s
	SLA			-		0.22*	n.s
	F_v_’F_m_’			-			0.39**
3	A_n_	0.57**	n.s	-	n.s	0.82**	0.77**
	g_sw_		-0.65**	-	n.s	0.49**	0.27**
	WUE_i_			-	n.s	n.s	n.s
	SLA			-		n.s	n.s
	F_v_’F_m_’			-			0.73**

A_n =_ net photosynthetic rate (μmol CO_2_m^-2^ s^-1^); g_sw_ = stomatal conductance to water vapour (molH_2_Om^-2^ s^-1^); WUE_i_ = intrinsic water use efficiency (μmol CO_2_ molH_2_O^-1^); δ^13^C = isotopic composition of ^13^C (‰); SLA = specific leaf area (m^2^Kg^-1^); F_v_’F_m_’ = maximum efficiency of PSII under light conditions; Φ_PSII_ = quantum yield. Time-points of measurements correspond with: 1, well watered plants; 2, seven days without irrigation; 3, fourteen days without irrigation. *p < 0.05, **p < 0.01,***p < 0.001.

### Highly saturated linkage maps

For Gal1056, Oria6 and consensus map, 17, 16 and 13 linkage groups (LG) were obtained, respectively (Table 3). The three constructed genetic linkage maps had in total 2,107 markers representing 1,314 mapped genes (Table 3). Genes were evenly distributed between linkage groups (χ^2^ test p > 0.05 for the three linkage maps). Map coverage was 65–100% and average distance between two adjacent markers was smaller than 2 cM (Table 3). The vast majority of markers with distorted segregations were discarded because of insufficient linkage information to be mapped (Table 3). Out of the six distorted markers, five mapped in the first 10 and 20 cM of LG 5 in Oria6 and consensus maps, respectively (see Additional files 4 and 5).

**Table 3:** **Mapping features of the two parental linkage maps (Gal1056 and Oria6) and consensus map for the cross (GxO)**

Mapping features	Gal1056	Oria6	GxO
Total number of available markers	1,539	1,574	2,601
SSRs loci	8	7	8
SAMPL loci	29	33	55
SNP loci	1,502	1,534	2,538
Total number of distorted markers^a^	33 (2.1%)	36 (2.3%)	53 (2%)
Unlinked markers (%)	65 (4.2%)	78 (5%)	54 (2.1%)
Number of markers assigned to LG	1,474	1,496	2,547
SSRs loci	8	7	8
SAMPL loci	21	25	54
SNP loci	1,445	1,464	2,485
Number of positioned markers^b^	1,026 (66.7%)	1,184 (75.2%)	1,810 (69.6%)
SSR loci	2 (25%)	3 (42.9%)	1 (12.5%)
SAMPL loci	12 (41.4%)	12 (36.4%)	22 (40%)
SNP loci	1,012 (67.4%)	1,169 (76.2%)	1,787 (70.4%)
Number of positioned genes^c^	685	792	1,154
Number of distorted positioned markers	0	5	6
LG before alignments	17	16	13
Groups after alignments	12	12	12
Smallest LG before alignments	24 cM	28.7 cM	39.1 cM
Largest LG before alignments	141.9 cM	149.6 cM	165 cM
Average length LG ± SD before alignments (cM)	87.6 ± 42	92.9 ± 41.8	128.9 ± 31.8
Smallest group after alignments	76 cM	70.5 cM	116.1 cM
Largest groups after alignments	187.8 cM	149.6 cM	165 cM
Average length of a group ± SD after alignments (cM)	124.1 ± 26.9	123.9 ± 22.3	138.5 ± 17.1
Maximum distance between 2 adjacent markers	20 cM	28.8 cM	18.3 cM
Average distance between 2 adjacent markers ± SD^d^	1.92 ± 2.7	1.66 ± 2.6	1.24 ± 1.9
Observed map length (cM)	1,488.7	1,486.8	1,662.3
Estimated map length (cM)	2,337.7	1,479.7	2,378.2
Observed map coverage	64%	100%	69.9%
Estimated map coverage	100%	100%	100%

^a^At p < 0.01 after Bonferroni correction for the number of markers.

^b^Not positioned markers correspond to unlinked markers or markers which position could not be reliably estimated. Percentages calculated over the total number of available markers.

^c^Twenty one, 47 and 59 positioned contigs for Gal1056, Oria6 and GxO maps respectively, were not considered.

^d^Identical markers whose position was the same because of the lack of recombination between them were not considered.

SD: Standard deviation.

Through comparisons between both parental maps, as well as with previously developed maps for *P. pinaster*[20, 81, 82] based on 654 common markers, 12 groups could be identified for the three maps, which is in agreement with the haploid number of chromosomes for the species. Common markers among the different genetic maps compared mapped always in the same homologous LG excepting three markers (see Additional file 6): contigs FN696780 and AL749831 that mapped in LG 9 and LG 4 in Chancerel et al. [20] and in LG 7 and LG 9 in this study, respectively (see Additional files 4 and 5); contig CT577280 that mapped in LG 7 and LG 4 in the two different maps obtained in Chancerel et al. [20] while in Gal1056 and the consensus map it was mapped in LG 4.

For the 82% and 86% of contigs with more than one mapped SNP, they mapped at less than 1 cM in Gal1056 and Oria6 respectively. There was a significant exception for contig BX249015 that had one SNP mapped in LG 5 (BX249015-204) in Gal1056, Oria6 and the consensus map and the other SNP mapped in LG 8 (BX249015-289) in Oria6 and the consensus map (see Additional files 4 and 5), whereas this contig was mapped in LG 5 in Chancerel et al. [20].

The consensus linkage map is available at Dendrome (http://dendrome.ucdavis.edu/cmap/).

### QTL detection

**Figure 1:**
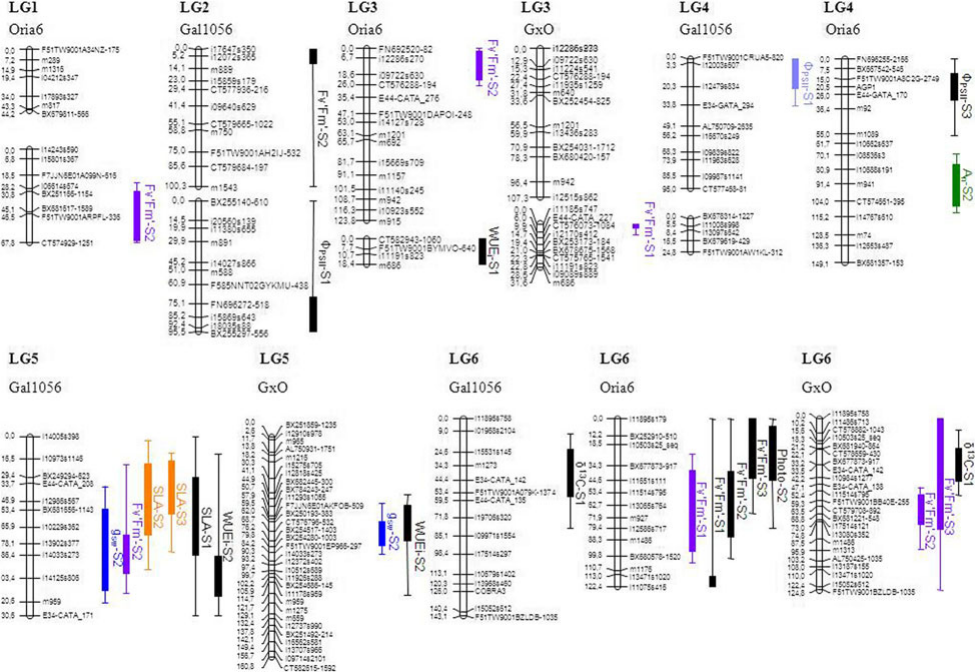
**Graphical representation of the QTL identified on the parental (Gal1056 and Oria6) and consensus (GxO) framework linkage maps (LGs 1 to 6).** Only linkage groups for which QTL have been detected are presented. The one and two LOD confidence intervals are indicated by squares and lines, respectively. Colored QTL are the significant QTL (significant at 99% at chromosome level or 95% at genome wide level), with each color representing a different trait, and black QTL are the suggestive QTL (significant at 95% confidence at chromosome level). A_n_ = net photosynthetic rate (μmol CO_2_m^-2^ s^-1^); g_sw_ = stomatal conductance to water vapour (molH_2_Om^-2^ s^-1^); WUE_i_ = intrinsic water use efficiency (μmol CO_2_ molH_2_O^-1^); δ^13^C = isotopic composition of ^13^C (‰); SLA = specific leaf area (m^2^Kg^-1^); F_v_’F_m_’ = maximum efficiency of PSII under light conditions; Φ_PSII_ = quantum yield. S1, S2 and S3 stand for 1^st^ time-point of measurement (well watered plants), 2^nd^ time-point of measurement (seven days without watering) and 3^rd^ time-point of measurement (14 days without watering) respectively.

**Figure 2:**
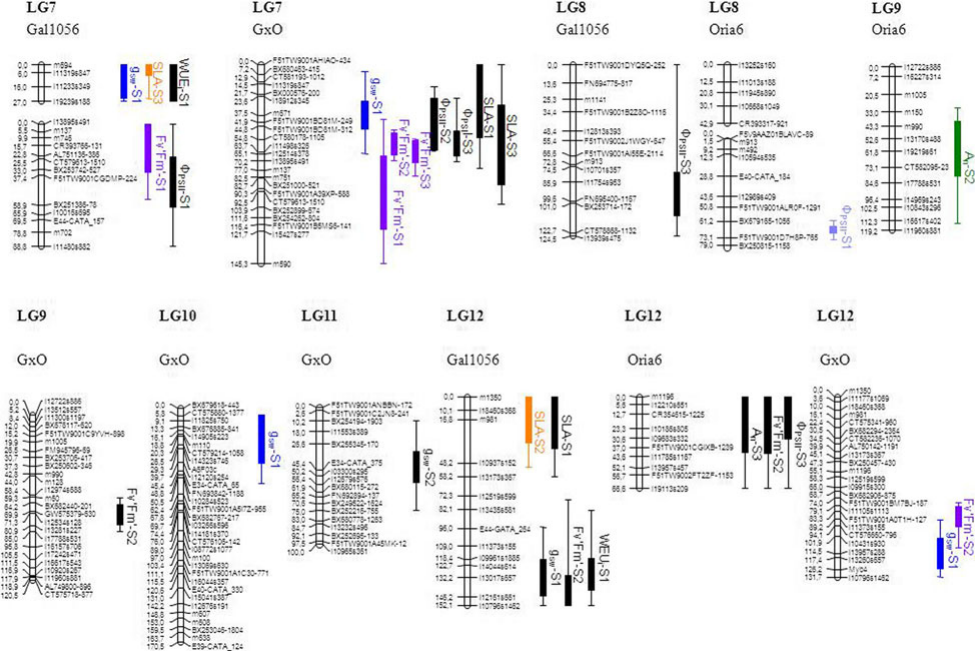
**Graphical representation of the QTL identified on the parental (Gal1056 and Oria6) and consensus (GxO) framework linkage maps (LGs 7 to 12).** Only linkage groups for which QTL have been detected are presented. The one and two LOD confidence intervals are indicated by squares and lines, respectively. Colored QTL are the significant QTL (significant at 99% at chromosome level or 95% at genome wide level), with each color representing a different trait, and black QTL are the suggestive QTL (significant at 95% confidence at chromosome level). A_n_ = net photosynthetic rate (μmol CO_2_m^-2^ s^-1^); g_sw_ = stomatal conductance to water vapour (molH_2_Om^-2^ s^-1^); WUE_i_ = intrinsic water use efficiency (μmol CO_2_ molH_2_O^-1^); δ^13^C = isotopic composition of ^13^C (‰); SLA = specific leaf area (m^2^Kg^-1^); F_v_’F_m_’ = maximum efficiency of PSII under light conditions; Φ_PSII_ = quantum yield. S1, S2 and S3 stand for 1^st^ time-point of measurement (well watered plants), 2^nd^ time-point of measurement (seven days without watering) and 3^rd^ time-point of measurement (14 days without watering) respectively.

Of the 55 detected QTL (Table 4, Figures 1 and 2), 28 were highly significant QTL, whereas the remaining 27 could be considered as suggestive or putative QTL. QTL were detected for all traits but the higher number of QTL were detected for F_v_’F_m_’ and Φ_PSII_ (Table 4, Figures 1 and 2). The total phenotypic variance explained for a single QTL ranged from 4.6% (WUE_i_) to 20.9% (F_v_’F_m_’). The higher percentage of total phenotypic variance explained by all the QTL detected for a trait in a time-point of measurement was 44% (F_v_’F_m_’).

**Table 4:** **Identified QTL in Gal1056, Oria6 and GxO maps**

Trait	Time-point	Map	Total var.	LG	LOD	Sig.	Var.	Add. Eff.	Position (cM)	CI _1LOD_ (cM)	CI _2LOD_ (cM)	QTL id
A_n_	2	Oria6	23	4	2.7	**ch	10	+	91.4	77.1-107.4	69.7-112.4	A_n_S2LG4m
				6	2	*ch	7.1	-	18.5	5-44.5	0-79.5	A_n_S2LG6m
				9	2.4	**ch	8.7	+	61.8	40-79.8	30-113.8	A_n_S2LG9m
	3	Oria6		12	1.5	*ch	12	-	30.6	0-41	0-66.6	A_n__S3LG12m
g_sw_	1	Gal1056	18.7	7_1	1.9	**ch	7.4	+	0.0	0-25	0-27	g_sw_ S1LG7_1f
				12	2.1	*ch	8	+	132.2	119-145.2	95.1-152.1	g_sw_ S1LG12f
		GxO	37.8	7	3.5	**ch	10.7		37.5	27-47	5-65	g_sw_ S1LG7i
				10	4.1	**ch	12.5		16.1	5-40	5-55	g_sw_ S1LG10i
				12	3.9	**ch	12		114.5	103.1-125.5	90-131.6	g_sw_ S1LG12i
	2	Gal1056		5	2.4	**ch	10.1	+	86.4	52.9-112.3	36.7-121.6	g_sw_ S2LG5f
		GxO	24.1	5	3.9	**ch	14.4		68.7	61.9-79.7	48.3-85.7	g_sw_ S2LG5i
				11	2.8	*ch	10		50.1	32.6-55.1	10-75	g_sw_ S2LG11i
WUE_i_	1	Gal1056	15	7_1	1.2	*ch	4.7	-	6.0	0-27	0-27	WUE_i_S1LG7_1f
				12	2.0	*ch	7.8	-	132.2	118-141.2	83.1-152.1	WUE_i_S1LG12f
		Oria6		3_2	1.06	*ch	4.6	+	0	0-18.4	0-18.4	WUE_i_S1LG3_2m
	2	Gal1056		5	2.1	*ch	9	-	103.3	87.4-116.3	13-130.6	WUE_i_S2LG5f
		GxO		5	3	*ch	12.7		68.7	49.9-76.2	42.1-115.7	WUE_i_S2LG5i
δ^13^C	1	Gal1056		6	2.3	*ch	9.7	+	34.3	22-56.4	11-79.8	δ^13^C S1LG6f
		GxO		6	3.2	*ch	13.4		33.2	21.2-45.2	8-55.4	δ^13^C S1LG6i
SLA	1	Gal1056	16.2	5	1.9	*ch	7.5	+	53.4	29.4-86.4	0-130.6	SLAS1LG5f
				12	2.2	*ch	8.8	-	10.1	0-38.1	0-58.2	SLAS1LG12f
		GxO		7	3.1	*ch	13.1		12.9	0-53.8	0-76.1	SLAS1LG7i
	2	Gal1056	18.6	5	2.5	**ch	9.7	+	46.9	20-72	3-96.9	SLAS2LG5f
				12	2.6	**ch	9.8	-	10.1	0-34.1	0-51.1	SLAS2LG12f
	3	Gal1056	16.8	5	2.5	**ch	9.7	+	46.9	17.5-55.9	12-83.9	SLAS3LG5f
				7_1	2.2	**ch	8.5	+	0.0	0-8	0-25	SLAS3LG7_1f
		GxO		7	3.1	*ch	13.1		44.8	29.6-87.7	0-101.5	SLAS3LG7i
F_v_'F_m_'	1	Gal1056		7_2	2.9	*gw	12.1	-	5.5	0-35	0-54.4	F_v_’F_m_’S1LG7_2f
		Oria6	18	6	1.9	*ch	7.4	+	122.4	114.7-122.4	0-122.4	F_v_’F_m_’S1LG6m1
				6	2.7	**ch	10.3	-	62.7	37.5-96.7	25.5-104.7	F_v_’F_m_’S1LG6m2
		GxO	28.2	3_2	3.5	**ch	12		0	0-3	0-8	F_v_’F_m_’S1LG3_2i
				7	3.7	**ch	12.9		90.3	67-120	40-145.3	F_v_’F_m_’S1LG7i
	2	Gal1056	22.9	2_1	1.9	*ch	7	-	0.0	0-11	0-100.3	F_v_’F_m_’S2LG2_1f
				5	2.7	**ch	10	+	86.5	71.9-99.4	20.5-114.4	F_v_’F_m_’S2LG5f
				12	2.1	*ch	7.5	+	152.1	130.6-152.1	75.2-152.1	F_v_’F_m_’S2LG12f
		Oria6	32.7	1_2	2.6	**ch	8.2	-	45.1	30.2-66.1	24.5-67.8	F_v_’F_m_’S2LG1_2m
				3_1	3.3	**gw	10.8	+	18.6	2-22.6	0-26.6	F_v_’F_m_’S2LG3_1m
				6	2	*ch	6.4	-	62.7	38.3-85.7	0-101.7	F_v_’F_m_’S2LG6m
				12	1.7	*ch	5.4	-	30.6	0-41.6	0-66.6	F_v_’F_m_’S2LG12m
		GxO	44	6	4.1	**ch	11.4		59.3	55-77	50-95	F_v_’F_m_’S2LG6i
				7	7	**gw	20.9		54.8	50-66	48-70	F_v_’F_m_’S2LG7i
				9	3.6	*ch	9.6		71.3	65-80	60-85	F_v_’F_m_’S2LG9i
				12	4.7	**gw	13.2		83.3	80-95	77-110	F_v_’F_m_’S2LG12i
	3	Oria6		6	2.1	*ch	9	-	34.3	0-43.3	0-68.7	F_v_’F_m_’S3LG6m
		GxO	29.1	6	3.7	**ch	13.3		54.8	0-80	0-124.8	F_v_’F_m_’S3LG6i
				7	4.5	*gw	15.6		63.7	55.8-71.6	54.5-81.6	F_v_’F_m_’S3LG7i
Φ_PSII_	1	Gal1056	20.1	2_2	1.8	*ch	6.8	-	85.2	69.9-95.5	0-95.5	Φ_PSII_ S1LG2_2f
				4_1	2.7	**ch	10.1	+	3.3	0-22.3	0-34.3	Φ_PSII_ S1LG4_1f
				7_2	1.8	*ch	6.5	-	37.4	23.8-60.4	0-88.8	Φ_PSII_ S1LG7_2f
		Oria6		8_2	2.7	**ch	11.3	-	79	74.1-79	60.8-79	Φ_PSII_ S1LG8_2m
	2	GxO		7	2.9	*ch	12.3		37.5	24.6-62.8	16.5-62.8	Φ_PSII_ S2LG7i
	3	Gal1056		8	1.8	*ch	7.8	-	99.6	78.5-110	0-124.5	Φ_PSII_ S3LG8f
		Oria6	15.1	4	2	*ch	7.9	+	20.5	10.5-30.5	0-56.5	Φ_PSII_ S3LG4m
				12	1.9	*ch	7.3	-	12.7	0-30.7	0-66.5	Φ_PSII_ S3LG12m
		GxO		7	2.9	*ch	12.2		63.7	48.8-67.1	24.6-71.2	Φ_PSII_ S3LG7i

Columns stand for trait names [A_n_ = net photosynthetic rate (μmol CO_2_m^-2^ s^-1^); g_sw_ = stomatal conductance to water vapour (molH_2_Om^-2^ s^-1^); WUE_i_ = intrinsic water use efficiency (μmol CO_2_ molH_2_O^-1^); δ^13^C = isotopic composition of ^13^C (‰); SLA = specific leaf area (m^2^Kg^-1^), F_v_’F_m_’ = maximum efficiency of PSII under light conditions; Φ_PSII_ = quantum yield], time-points of measurements (1^st^ stands for well watered, 2^nd^ and 3^rd^ for seven and 14 days without watering), genetic map where the QTL was identified, total phenotypic variance explained (%) for all detected QTL for a given trait in a given time-point of measurements, linkage group, maximum LOD score for mapped markers, level of significance (* <0.05, ** < 0.01, ch stands for chromosome and gw for genome wide level), total phenotypic variance explained for each QTL (%), sign of the additive effect, position of the marker with the maximum LOD score, one LOD confidence interval, two LOD confidence interval and QTL identification name.

Consequently, four QTL hotspots could be identified in LG 5, LG 6, LG 7 and LG 12 (Table 4, Figures 1 and 2) due to the co-localization of QTL for different traits (Figures 1 and 2): SLA co-localized with g_sw_ in LG 5 and LG 7; F_v_’F_m_’ co-localized with WUE_i_ in LG 5 and LG 12 with δ^13^C in LG 6, with A_n_ in LG 6 and LG 12 and with Φ_PSII_ in LG 7 and LG 12. QTL for SLA, F_v_’F_m_’ and Φ_PSII_ were detected for the three time-points of measurement. Some of them co-localized in the same region, such as the identified for SLA in LG 5, LG 7 and LG 12 and for F_v_’F_m_’ in LG 6 and LG 7. Co-localization of QTL for the same traits at different levels of water stress highlights the stability of QTL with the imposition of drought stress. QTL for A_n_ could only be detected for the 2^nd^ and 3^rd^ time-point of measurement while QTL for g_sw_ and WUE_i_ were only detected in the 1^st^ and 2^nd^ time-point of measurement (Table 4).

### Candidate gene identification

**Figure 3:**
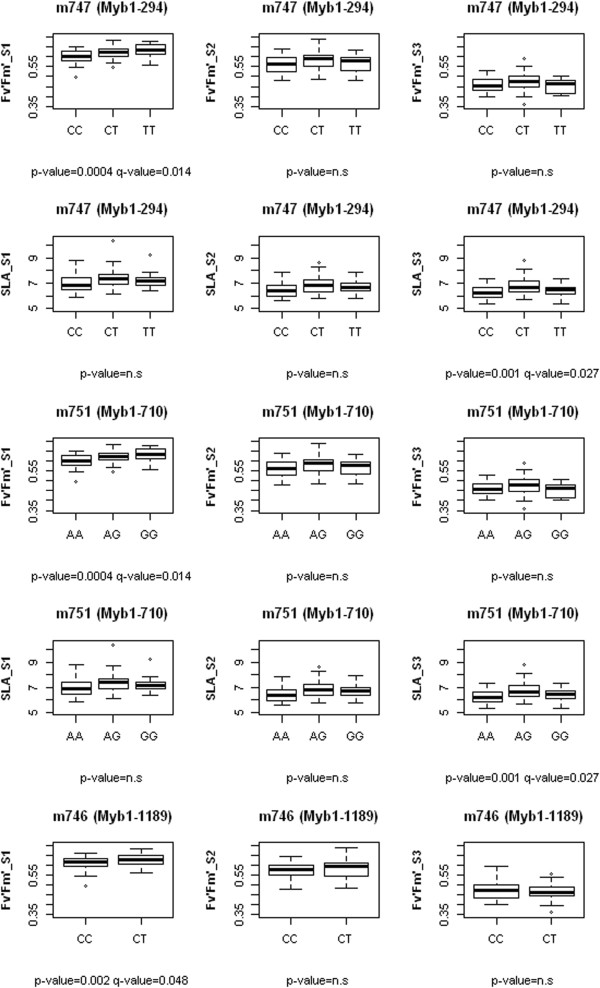
**Boxplots for SNPs in candidate gene MYB 1 significantly associated with traits.** For each one of the three SNPs (gene name and SNP position between brackets) is shown the p-value of ANOVA and false discovery rate (q-value) estimated for F_v_’F_m_’ (maximum efficiency of PSII under light conditions) and SLA (specific leaf area). S1, S2 and S3 stand 1^st^, 2^nd^ and 3^rd^ time-point of measurements respectively.

The 74% of the mapped sequences (991 out of 1,348) were annotated. Gene annotations and co-localization with the detected QTL lead to the identification of 58 positional candidate genes that could be involved in the expression of the targeted traits (see Additional file 7). Genes related with oxidative stress, ATPase family proteins or proteins of the light harvesting centers were found in the confidence intervals of QTL for net photosynthesis or chlorophyll fluorescence traits. Genes related with stomatal regulation, ABA signaling pathways or cell wall composition were found in QTL for g_sw_ and WUE_i_. Genes expressed under drought conditions co-localized with QTL identified in the 2^nd^ or 3^rd^ time-point of measurements but not in the first one, which could be pointing out the induced drought functional response of *P. pinaster*. Other remarkable co-localizations were found for two QTL for SLA with an enzyme involved in auxin biosynthesis, or between a QTL for δ^13^C and a member of the aquaporin family (see Additional file 7 for a detailed list of candidate genes). ANOVA test developed for the 73 tested SNPs in candidate genes resulted in 43 significant associations with at least one of the analyzed traits (data not shown). After corrections using the false discovery rate estimated, only three SNPs of the gene *MYB1* (m746, m747 and m751) remained significantly associated with F_v_’F_m_’ measured in the 1^st^ time-point (well watered plants) and SLA measured in the 3^rd^ time-point (14 days without watering; Figure 3). SNP m746 was located in an intron but m747 and m751 were located in exon regions. The base substitution in SNP m747 was a non-synonymous change between a threonine (when a cytosine is present) and an isoleucine (when a thymine is present) while in m751 was a synonymous change. SNPs m747 and m751 explained 14.4% and 12.6% of the phenotypic variance for F_v_’F_m_’ measured in the 1^st^ time-point and SLA measured in the 3^rd^ time-point respectively. SNP m746 explained 9% of the total phenotypic variance for F_v_’F_m_’ measured in the 1^st^ time-point.

## Discussion

### Highly saturated linkage maps

Combining different types of markers three highly dense linkage maps were constructed. They include more than 1,000 genes scattered throughout the genome of *P. pinaster* and distributed in 12 groups that match the chromosome number of the species. The aforementioned highly saturated maps, with less than 2 cM mean distance between markers, are in the range of recently published linkage maps for other conifer species [20, 22, 24, 27, 51]. Estimated map length was higher in the female than in the male parent. Differences in genome length between parental maps are usually found in conifer species [90–93] and it may be a consequence of differences in the recombination rate between parental trees [94, 95].

The accuracy of the SNP genotyping assays previously proved [20, 82] has been confirmed in this study by genes with more than one SNP that mapped in almost all cases within a distance lower than 1 cM. The single exception of contig BX249015 could be attributed to the existence of two paralogous genes for this sequence placed in different LGs. Indeed, high levels of synteny and colinearity were observed between female and male parental maps. The fact that four out of the five distorted markers mapped in the same region suggests that segregation distortion could be due to pre or post-zygotic selection rather than to genotyping errors.

The construction of dense genetic maps for different conifers provides additional tools for studying conifer genomes organization and evolution at a finer scale [27]. In addition, high density linkage maps can be used to position scaffolds along linkage groups contributing to the assembly of a reference genome sequence [24, 96]. *P. pinaster* genome sequencing is currently in progress, and it should be noted that Oria6, the male progenitor of the mapping family, is the genotype from which the haploid line was selected and its DNA used as template [97].

Additionally, development of dense genetic maps from individuals belonging to two Spanish natural populations (from Northwest coast and Southeast mountains) that show high levels of genetic divergence with the Spanish (from the Castilian Plateau; [81]) and French populations (from Landes and Corsica; [20]), from which segregating progenies have been previously mapped, is important to explore the genetic organization and evolution of the species. Synteny and colinearity were highly conserved when compared with 654 common markers with previous studies [20, 81, 82]. Only three discrepancies were found that supposed just a 0.46% over all the common markers analyzed: Contig CT577280 was mapped in LG 7 and LG 4 in two of three obtained maps for *P. pinaster* in Chancerel et al. [20] and it was suggested the existence of two paralogous genes for this sequence. In this study the position of CT577280 in LG 4 was confirmed. Contigs FN696780 and AL749831 mapped in LG 9 and LG 4 in Chancerel et al. [20] and in LG 7 and LG 9 in this study respectively, which suggest also the existence of two paralogous genes for these sequences.

The high level of synteny and colinearity observed between the genetic maps developed for individuals that belong to populations with very different genetic backgrounds [98] points out the high reliability in the marker order obtained. Thus, it is possible the development of a composite genetic map for the species by integrating the genetic maps developed by de Miguel et al. [81], Chancerel et al. [20] and those obtained in this work, which is currently in progress. Parental maps are the most accurate regarding both, marker order and marker distances; since they have been constructed through separated information of the meiosis occurred in each progenitor. Accuracy is also related with the presence of genotyping errors, missing values and segregation distortion in the molecular marker data used for the construction of linkage maps [99]. In this study it was achieved by the thorough genotypic data integrity obtained by using highly stringent thresholds to consider SNPs for mapping. Also, the position of the SNPs genotyped in fewer individuals (SNP array D) was validated through the comparison with previously developed *P. pinaster* maps [20]. In addition, only a few distorted markers have been mapped and almost all located in a narrow region of a single linkage group (Oria6 and GxO LG 5), which points towards a probable biological origin. Even so, QTL analyses have been developed using framework linkage maps to minimize the problems that possible errors in marker order could cause.

### QTL detection

One of the main goals of this work was to identify QTL for leaf functional traits related to photosynthesis and water use efficiency in response to drought. QTL analysis in forest tree species is challenging by its long generation times which hinder the development of classical mapping populations like backcross, F_2_ or recombinant inbred lines. In order to overcome this shortcoming alternative strategies are usually developed for QTL detection in trees, such as the two-way pseudo-testcross [83] used in this study. In this work, two parents from contrasting populations in their drought response were selected to maximize the variability of the F_1_ obtained progeny, at molecular and functional levels. Although some recent QTL studies in trees worked with larger progenies [51, 100, 101], the 162 obtained siblings in this study are in the range or higher than other QTL analysis in trees [31, 46, 69, 70, 102]. On the other side, gas exchange parameters are extremely sensible to variations in the environmental conditions. To cope with the problem of environmental noise in phenotypic evaluation, different strategies have been used for QTL analysis in the literature. For example the implementation of statistical and physiological models to adjust phenotypic values for microclimatic differences [41, 50] or the development of inbred line populations for annual species [103, 104]. In this study, four vegetative copies of each genotype were established in a completely randomized block design in a greenhouse. All these efforts allowed the identification of significant and suggestive QTL for important traits related to water use efficiency and carbon uptake in *P. pinaster*.

For all the analyzed traits several QTL were identified with moderate effects rather than a single or few QTL with large effect, as expected for complex functional traits. The only exception was δ^13^C for which only one QTL could be identified. For gas exchange parameters, the percentage of observed phenotypic variance explained when taking into account all significant QTL detected in a single trait in each time-point of measurements was in average 20%. In addition, some suggestive QTL for water use efficiency estimated from different approaches have been reported, although their existence should be further tested using larger population sizes or analyzing their stability in different genetic backgrounds [105]. For chlorophyll fluorescence parameters, a large number of QTL were identified. The significant QTL detected in each time-point of measurement for F_v_’F_m_’ explained together up to 44% of the observed phenotypic variance. The results achieved point towards a tight genetic control of photochemical traits, as previously described in crop species like maize [103, 106], barley [104] or soybean [40].

For δ^13^C only one QTL was detected in LG 6, in agreement with Brendel et al. [44] that found a QTL in the same region of LG 6 for *P. pinaster*. No co-localization of QTL for WUE_i_ and δ^13^C was found in spite of the significant phenotypic and genetic correlation between both traits. In this study δ^13^C was measured only in the 1^st^ time-point of measurement, thus its value probably reflected the water use efficiency in well watered conditions. As the genotypes analyzed have showed high genetic variability in water use efficiency in response to drought [76], it could be expected to observe higher variation in δ^13^C in needles developed under water limiting conditions maintained in a long-lasting water stress period [107–109]. Higher variability on δ^13^C would enhance the detection of QTL for this trait and it might be possible to find other QTL as reported by Brendel et al. [44], who found four significant and four suggestive QTL. Differences in the number of detected QTL for δ^13^C between both studies could also be explained because Brendel et al. [44] measured δ^13^C in tree growth rings from 15 years-old trees while in this study δ^13^C was measured in needles of one year-old seedlings. Nevertheless, the co-localization of this QTL for δ^13^C between two genetically unrelated progenies from wide geographic origins (Landes x Landes versus Galicia x Oria) and growing under different environmental conditions supports its stability.

Interaction between QTL and environmental conditions was tested performing the QTL analyses using three different time-points of measurements corresponding to different water irrigation regimes. In general, most of the detected QTL were environment-specific, suggesting that genes are differentially activated during maritime pine drought response [110]. Nevertheless, several QTL for F_v_’F_m_’, Φ_PSII_ and SLA were less sensitive to environmental conditions and maintained the same location with drought imposition, confirming the stability of these QTL across different levels of water stress endured by plants.

This way, four clusters of QTL were identified in LG 5, LG 6, LG 7 and LG 12. Clustering of QTL could be related with the pleiotropic effect of one or a few genes affecting different traits rather to the existence of rich gene regions, as genes were homogeneously distributed between LGs. Chancerel et al. [20] detected higher number of genes in LG 6 and LG 12 than in the other linkage groups, however the maps developed in this study could not confirm these results.

QTL for photosynthesis measured through gas exchange and chlorophyll fluorescence parameters co-localized in LG 6 and LG 12, accordingly with the high broad-sense genetic correlation found between both traits. However, additional and no co-localizing QTL were identified for these traits in other LGs, suggesting that CO_2_ fixation and electron transport were not entirely coupled, in agreement with Gu et al. [41]. Uncoupling of these two processes may be due to drought effects on stomatal conductance, biochemical alterations of carbon fixation enzymes, or photoinhibition affecting electron transport rate [7, 8].

Under drought stress A_n_ and g_sw_ showed a lower level of phenotypic correlation while the correlation coefficient between A_n_ and F_v_’F_m_’ or Φ_PSII_ increased with water stress, which suggests that under stomatal closure the differences that can be observed between genotypes in carbon fixation could be due to differences in electron transport through PSII rather than to differences in stomatal conductance, as previously observed in other species [106].

SLA showed a significant phenotypic and genetic correlation with WUE_i_ and F_v_’F_m_’. The identification of relationships between two traits using phenotypic correlations may not distinguish whether the traits could be causally related or simply varying in association. However, the coincidence of QTL for two traits is strong evidence that they could be functionally related [36]. QTL co-localization of SLA with WUE_i_ and F_v_’F_m_’ was found in LG 5 and LG 7 pointing towards a strong inter-relationship between SLA, WUE_i_ and F_v_’F_m_’. The aforementioned co-localization could indicate that plants with lower SLA are more efficient in water use but had a lower efficiency of electron transport through photosystem II that could be explained because of the higher importance of g_sw_ over A_n_ in determining WUE_i_ in this species [76, 111–113].

Most of the detected QTL were found only in one of the two progenitors. The parental trees were selected from two distant populations, showing high level of genetic differentiation, and with a different degree of drought tolerance. Oria6 came from the southeast of Spain governed by a Mediterranean climate with long, hard and frequent summer dry periods, while Gal1056 came from the northwest of Spain where Atlantic climate is present. Consequently, a higher degree of drought adaptation is expected in Oria6 than in Gal1056. Controlled crosses performed with so different parental trees in their response to drought are very useful to compare QTL identified in individuals with different genetic backgrounds.

### Candidate genes within QTL

The identification of the gene or genes underlying a trait has been described as one of the greatest challenge for geneticists during this century [114]. The development of high density linkage maps using gene-based markers selected, in some cases, for their known implication in drought response allowed the identification of potential candidate genes for the quantitative multigenic traits analyzed in this study. Due to the lack of sequence annotation, a considerable number of mapped sequences showing high homology with cDNA sequences from other conifers could not be functionally inspected. Thus, some QTL with large effect had no obvious candidate genes but hold great promise to identify unknown genes underlying the corresponding processes in the future. For other QTL, positional candidate genes with known function in other species that were selected according to their functional similarity with genes involved in processes related with the studied trait were identified. A MIXTA-LIKE TRANSCRIPTION FACTOR (MYB) and a HISTONE CHAPERONE were found at 25 and 12 cM from the LOD peak of one of the four most clearly detected QTL, F_v_’F_m_’S2LG7i. MYB transcription factors are a wide group related with multiple physiological processes such as photosynthesis signaling [115]. The HISTONE CHAPERONE acts as a heat protection protein [116]. The increase of leaf temperature could be an important consequence under drought stress conditions due to reduced transpiration caused by stomatal closure. In this sense, the gene encoding the MYB transcription factor and the HISTONE CHAPERONE also co-localized with F_v_’F_m_’S3LG7i, both QTL measured under water stress. Another gene of the *MYB* family encoding the MYB 1 transcription factor, co-localized with several QTL for F_v_’ F_m_’ and Φ_PSII_ measured in well watered conditions (F_v_’F_m_’S1LG7i, F_v_’F_m_’S1LG7_2f, Φ_PSII_ S1LG7_2f) and SLA measured under water stress (SLAS3LG7i). In this sense, three SNPs positioned in *MYB 1* gene resulted in a significant association with F_v_’F_m_’ measured in the 1^st^ time-point of measurements (well watered plants) and SLA measured in the 3^rd^ time-point of measurements (14 days without watering). Lepoittevin et al. [117] found that the gene *MYB 1* showed complete linkage disequilibrium in *P. pinaster* over a distance of 1,304 bp. Together with their intron/exon location and base substitution types, this points towards association of SNPs m751 and m746 with target traits could be the consequence of genetic linkage with m747, that had higher chance to influence F_v_’F_m_’ and SLA. The expression of MYB 1 regulates genes of the phenylalanine pathway in white spruce [118] and maritime pine [119]. Increase of isoprenoid related compounds has been described to be related with photoprotection mechanisms triggering under abiotic stresses [120]. In this respect, some of the SNPs observed for *MYB 1* at present could be related with enhancing maintenance of photochemistry function as higher F_v_’F_m_’ during drought. These associations should be further validated analyzing, i.e. specific nucleotide variants in a panel of unrelated genotypes [121].

Several genes related with oxidative stress co-localized with QTL for photosynthesis under water stress conditions inferred both by gas exchange or chlorophyll fluorescence. For example, 5 -ADENYLSULFATE REDUCTASE-LIKE 4-LIKE that was implicated in the cell redox homeostasis [122], co-localized with QTL Φ_PSII_S1LG8_2m; PROLYL 4-HYDROXYLASE ALPHA SUBUNIT-LIKE PROTEIN that has oxidoreductase activity [123], co-localized with QTL A_n_S2LG9m; or CINNAMOYL- REDUCTASE 1-LIKE and PEROXIREDOXIN- CHLOROPLASTIC-LIKE that are enzymes from the flavonoid and phenylpropanoid biosynthesis pathways, respectively [124], were on the confidence interval of QTL F_v_’F_m_’S2LG1_2m. Overall, gene annotation seems to point out to an important role of maintenance photochemical integrity machinery in the drought response of *P. pinaster*.

Several genes that have been described to be related with regulation of stomatal aperture were found in the range of QTL for g_sw_ and WUE_i_. For example, MALATE DEHYDROGENASE catalyzes the reaction which converts malate to oxalacetate and a reduction in malate before stomatal closure was observed [125, 126]. Also, PHOSPHOLIPASE C 3-LIKE is required for the control of stomatal aperture by ABA [127, 128]. Genes encoding these enzymes co-localized with QTL g_sw_S2LG5f, WUE_i_S2LG5f, WUE_i_S2LG5i. AQUAPORIN NIP1-2-LIKE co-localized with δ^13^CS1LG6i, which was found interesting because of the importance of aquaporins in determining the leaf water status [129] and the proved stability of this QTL.

## Conclusions

The in-depth analysis of genetic control of the CO_2_ fixation process in response to drought was possible after measuring different functional parameters using complementary techniques, such as gas exchange and chlorophyll fluorescence, that measure final carbon capacity uptake. The use of maritime pine replicated genotypes and a suitable experimental design have made possible to identify genetic control for functional and morphological leaf traits, measured under three water irrigation regimes as they are highly dependent on environmental conditions. Several genomic regions implicated in the genetic control of drought resistance traits have been identified. The identification of potential candidate genes leads this project a step beyond the simple detection of QTL. Nonetheless, further association studies with proposed candidate genes are needed in order to validate detected SNP marker-trait associations.

## Electronic supplementary material

Additional file 1: **Identical markers based on recombination rate.** Not positioned markers correspond to unlinked markers or markers which position could not be reliably estimated. (XLSX 31 KB)

Additional file 2: **Broad sense genetic correlations (±standard error) between the analyzed traits.** (DOCX 13 KB)

Additional file 3: **Broad sense heritability (estimate ± standard error).** (DOCX 13 KB)

Additional file 4: **Parental linkage maps for Gal1056, Oria6 and consensus map for both progenitors (GxO).** (PDF 281 KB)

Additional file 5: **Mapped markers in parental linkage maps for Gal1056, Oria6 and consensus map for both progenitors (GxO).** (XLSX 213 KB)

Additional file 6: **Marker order comparison with maps obtained by Chancerel et al.** [20] **.** (PDF 63 KB)

Additional file 7: **Candidate genes within QTL** [130–180] **.** (XLSX 30 KB)
