# Supplementary material for: Genetic control of functional traits related to photosynthesis and water use efficiency in Pinus pinaster Ait. drought response: integration of genome annotation, allele association and QTL detection for candidate gene identification
Source: BMC Genomics. 2014 Jun 12;15(1):464. doi: 10.1186/1471-2164-15-464 (PMC4144121; doi:10.1186/1471-2164-15-464)
Supplement: Supplementary file 2 — Additional file 2: Broad sense genetic correlations (±standard error) between the analyzed traits. (DOCX 13 KB) [file 12864_2013_6163_MOESM2_ESM.docx]

**Additional file 2. Broad sense genetic correlations (± standard error) between the analyzed traits.** A_n_ = net photosynthetic rate (µmol CO_2_m^-2^s^-1^); g_sw_ = stomatal conductance to water vapour (molH_2_Om^-2^s^-1^); WUE_i_ = Intrinsic Water Use Efficiency (µmol CO_2_ molH_2_O^-1^); δ^13^C = isotopic composition of ^13^C (‰); SLA = Specific Leaf Area (m^2^Kg^-1^); Fv’Fm’ = maximum efficiency of PSII under light conditions; Φ_PSII_ = quantum yield. Time-points of measurements correspond with three levels of water stress (1, well watered plants; 2, seven days without irrigation; 3, fourteen days without irrigation). Only significant genetic correlations are presented. Genetic correlation between δ^13^Cand WUE_i_ was performed only in the first time-point of measurements while for the other traits was calculated for the three time-points of measurements. WUE_i_ was log-transformed (natural logarithm) to reach normal distribution of residuals. This table was partially redrawn from de Miguel et al. [74].

|  | g_sw_ | WUE_i_ | SLA | Fv’Fm’ | Φ_PSII_ | δ^13^C |
| --- | --- | --- | --- | --- | --- | --- |
| A_n_ | n.s | n.s | n.s | 0.93±0.01 | 0.81±0.03 |  |
| g_sw_ |  | -0.86±0.03 | n.s | n.s | 0.45±0.08 |  |
| WUE_i_ |  |  | -0.56±0.07 | n.s | n.s | 0.71±0.05 |
| SLA |  |  |  | 0.37±0.09 | n.s |  |
| Fv’Fm’ |  |  |  |  | 0.59±0.06 |  |
