# Supplementary material for: Genetic control of functional traits related to photosynthesis and water use efficiency in Pinus pinaster Ait. drought response: integration of genome annotation, allele association and QTL detection for candidate gene identification
Source: BMC Genomics. 2014 Jun 12;15(1):464. doi: 10.1186/1471-2164-15-464 (PMC4144121; doi:10.1186/1471-2164-15-464)
Supplement: Supplementary file 3 — Additional file 3: Broad sense heritability (estimate ± standard error). (DOCX 13 KB) [file 12864_2013_6163_MOESM3_ESM.docx]

**Additional file 3. Broad sense heritability (estimate ±standard error).** A_n_ = net photosynthetic rate (µmol CO_2_m^-2^s^-1^); g_sw_ = stomatal conductance to water vapour (molH_2_Om^-2^s^-1^); WUE_i_ = Intrinsic Water Use Efficiency (µmol CO_2_ molH_2_O^-1^); δ^13^C = isotopic composition of ^13^C (‰); SLA = Specific Leaf Area (m^2^Kg^-1^); Fv’Fm’= maximum efficiency of PSII under light conditions; Φ_PSII_ = quantum yield. Time-points of measurements correspond with three levels of water stress (1, well watered plants; 2, seven days without irrigation; 3, fourteen days without irrigation). Estimates significantly different from zero are in bold. WUE_i_ was log-transformed (natural logarithm) to reach normal distribution of residuals. This table was partially redrawn from de Miguel et al. [74].

|  | Pool data | 1^st^ series | 2^nd^ series | 3^rd^ series |
| --- | --- | --- | --- | --- |
| A_n_ | 0.04±0.05 | **0.10±0.05** | 0 | 0.01±0.05 |
| g_sw_ | **0.41±0.05** | **0.16±0.05** | **0.11±0.05** | 0.05±0.05 |
| WUE_i_ | **0.56±0.05** | **0.15±0.05** | **0.07±0.05** | 0 |
| SLA | **0.27±0.06** | **0.14±0.05** | **0.23±0.06** | **0.26±0.06** |
| Fv’Fm’ | **0.09±0.06** | **0.1±0.06** | **0.09±0.06** | **0.09±0.06** |
| Φ_PSII_ | **0.09±0.06** | 0.03±0.05 | **0.11±0.06** | 0.03±0.05 |
| δ^13^C |  | **0.34±0.08** |  |  |
