# Supplementary material for: Genetic control of functional traits related to photosynthesis and water use efficiency in Pinus pinaster Ait. drought response: integration of genome annotation, allele association and QTL detection for candidate gene identification
Source: BMC Genomics. 2014 Jun 12;15(1):464. doi: 10.1186/1471-2164-15-464 (PMC4144121; doi:10.1186/1471-2164-15-464)
Supplement: Supplementary file 4 — Additional file 4: Parental linkage maps for Gal1056, Oria6 and consensus map for both progenitors (GxO). (PDF 281 KB) [file 12864_2013_6163_MOESM4_ESM.pdf]

Figure S1. **Parental linkage maps for Gal1056, Oria6 and consensus map (GxO).** Markers marked in bold in Gal1056 and Oria6 maps are common markers between them. Markers with an asterisk in LG5 and LG10 are distorted markers at $p<0.01$ after Bonferroni correction. Homolog markers between parental and consensus map are linked with a solid line. Markers with special features (see results section) are marked in red.

LG 1

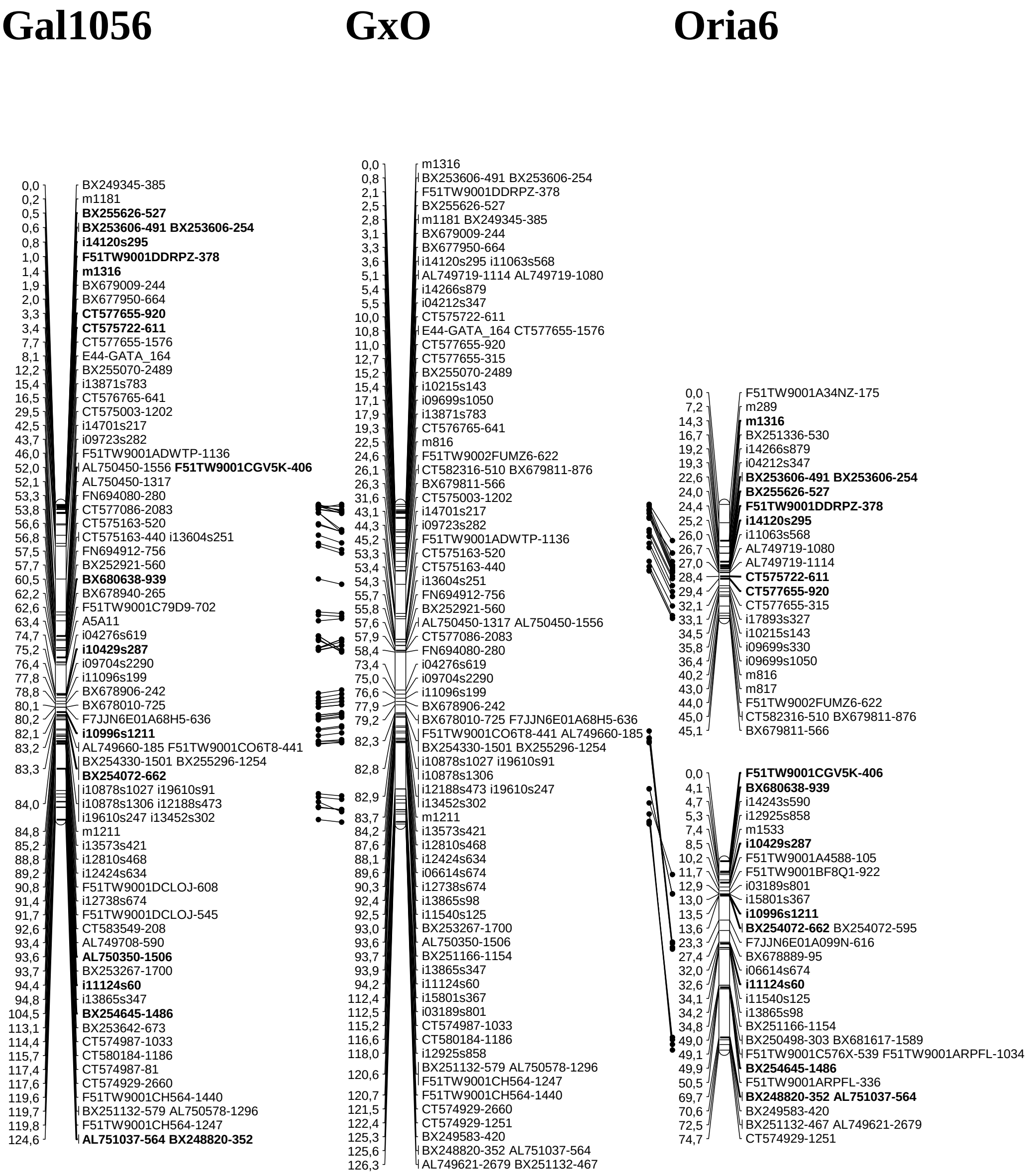

Gal1056
GxO
Oria6
0,0 BX249345-385
0,2 m1181
0,5 BX255626-527
0,6 BX253606-491 BX253606-254
0,8 i14120s295
1,0 F51TW9001DDRPZ-378
1,4 m1316
1,9 BX679009-244
2,0 BX677950-664
3,3 CT577655-920
3,4 CT575722-611
7,7 CT577655-1576
8,1 E44-GATA_164
12,2 BX255070-2489
15,4 i13871s783
16,5 CT576765-641
29,5 CT575003-1202
42,5 i14701s217
43,7 i09723s282
46,0 F51TW9001ADWTP-1136
52,0 AL750450-1556 F51TW9001CGV5K-406
52,1 AL750450-1317
53,3 FN694080-280
53,8 CT577086-2083
56,6 CT575163-520
56,8 CT575163-440 i13604s251
57,5 FN694912-756
57,7 BX252921-560
60,5 BX680638-939
62,2 BX678940-265
62,6 F51TW9001C79D9-702
63,4 A5A11
74,7 i04276s619
75,2 i10429s287
76,4 i09704s2290
77,8 i11096s199
78,8 BX678906-242
80,1 BX678010-725
80,2 F7JJN6E01A68H5-636
82,1 i10996s1211
83,2 AL749660-185 F51TW9001CO6T8-441
83,3 BX254330-1501 BX255296-1254
BX254072-662
84,0 i10878s1027 i19610s91
i10878s1306 i12188s473
i19610s247 i13452s302
84,8 m1211
85,2 i13573s421
88,8 i12810s468
89,2 i12424s634
90,8 F51TW9001DCLOJ-608
91,4 i12738s674
91,7 F51TW9001DCLOJ-545
92,6 CT583549-208
93,4 AL749708-590
93,6 AL750350-1506
93,7 BX253267-1700
94,4 i11124s60
94,8 i13865s347
104,5 BX254645-1486
113,1 BX253642-673
114,4 CT574987-1033
115,7 CT580184-1186
117,4 CT574987-81
117,6 CT574929-2660
119,6 F51TW9001CH564-1440
119,7 BX251132-579 AL750578-1296
119,8 F51TW9001CH564-1247
124,6 AL751037-564 BX248820-352
0,0 m1316
0,8 BX253606-491 BX253606-254
2,1 F51TW9001DDRPZ-378
2,5 BX255626-527
2,8 m1181 BX249345-385
3,1 BX679009-244
3,3 BX677950-664
3,6 i14120s295 i11063s568
5,1 AL749719-1114 AL749719-1080
5,4 i14266s879
5,5 i04212s347
10,0 CT575722-611
10,8 E44-GATA_164 CT577655-1576
11,0 CT577655-920
12,7 CT577655-315
15,2 BX255070-2489
15,4 i10215s143
17,1 i09699s1050
17,9 i13871s783
19,3 CT576765-641
22,5 m816
24,6 F51TW9002FUMZ6-622
26,1 CT582316-510 BX679811-876
26,3 BX679811-566
31,6 CT575003-1202
43,1 i14701s217
44,3 i09723s282
45,2 F51TW9001ADWTP-1136
53,3 CT575163-520
53,4 CT575163-440
54,3 i13604s251
55,7 FN694912-756
55,8 BX252921-560
57,6 AL750450-1317 AL750450-1556
57,9 CT577086-2083
58,4 FN694080-280
73,4 i04276s619
75,0 i09704s2290
76,6 i11096s199
77,9 BX678906-242
79,2 BX678010-725 F7JJN6E01A68H5-636
82,3 F51TW9001CO6T8-441 AL749660-185
BX254330-1501 BX255296-1254
i10878s1027 i19610s91
82,8 i10878s1306
82,9 i12188s473 i19610s247
i13452s302
83,7 m1211
84,2 i13573s421
87,6 i12810s468
88,1 i12424s634
89,6 i06614s674
90,3 i12738s674
92,4 i13865s98
92,5 i11540s125
93,0 BX253267-1700
93,6 AL750350-1506
93,7 BX251166-1154
93,9 i13865s347
94,2 i11124s60
112,4 i15801s367
112,5 i03189s801
115,2 CT574987-1033
116,6 CT580184-1186
118,0 i12925s858
120,6 BX251132-579 AL750578-1296
F51TW9001CH564-1247
120,7 F51TW9001CH564-1440
121,5 CT574929-2660
122,4 CT574929-1251
125,3 BX249583-420
125,6 BX248820-352 AL751037-564
126,3 AL749621-2679 BX251132-467
0,0 F51TW9001A34NZ-175
7,2 m289
14,3 m1316
16,7 BX251336-530
19,2 i14266s879
19,3 i04212s347
22,6 BX253606-491 BX253606-254
24,0 BX255626-527
24,4 F51TW9001DDRPZ-378
25,2 i14120s295
26,0 i11063s568
26,7 AL749719-1080
27,0 AL749719-1114
28,4 CT575722-611
29,4 CT577655-920
32,1 CT577655-315
33,1 i17893s327
34,5 i10215s143
35,8 i09699s330
36,4 i09699s1050
40,2 m816
43,0 m817
44,0 F51TW9002FUMZ6-622
45,0 CT582316-510 BX679811-876
45,1 BX679811-566
0,0 F51TW9001CGV5K-406
4,1 BX680638-939
4,7 i14243s590
5,3 i12925s858
7,4 m1533
8,5 i10429s287
10,2 F51TW9001A4588-105
11,7 F51TW9001BF8Q1-922
12,9 i03189s801
13,0 i15801s367
13,5 i10996s1211
13,6 BX254072-662 BX254072-595
23,3 F7JJN6E01A099N-616
27,4 BX678889-95
32,0 i06614s674
32,6 i11124s60
34,1 i11540s125
34,2 i13865s98
34,8 BX251166-1154
49,0 BX250498-303 BX681617-1589
49,1 F51TW9001C576X-539 F51TW9001ARPFL-1034
49,9 BX254645-1486
50,5 F51TW9001ARPFL-336
69,7 BX248820-352 AL751037-564
70,6 BX249583-420
72,5 BX251132-467 AL749621-2679
74,7 CT574929-1251

LG 2

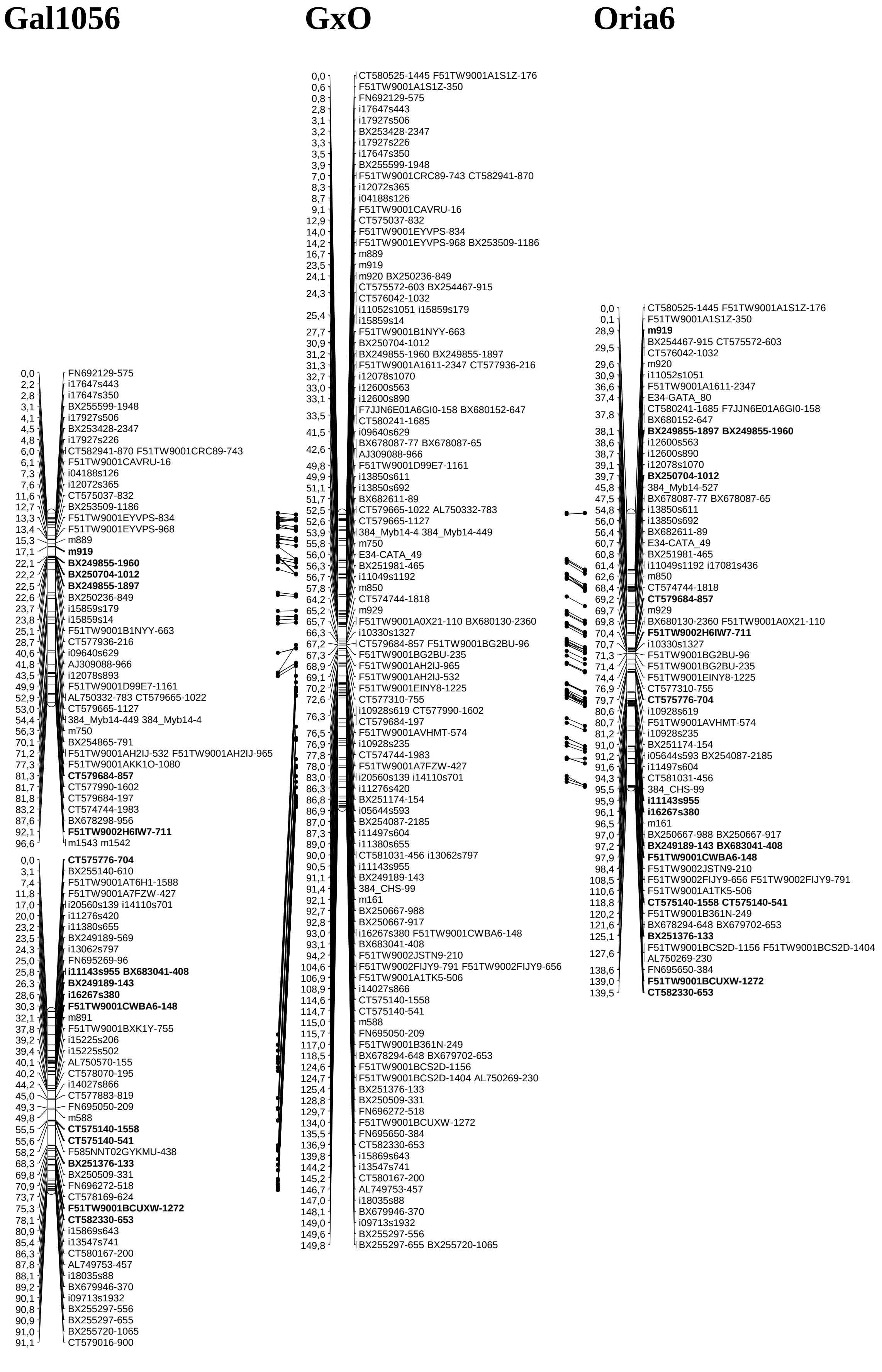
Gal1056
0,0 FN692129-575
2,2 i17647s443
2,8 i17647s350
3,1 BX255599-1948
4,1 i17927s506
4,5 BX253428-2347
4,8 i17927s226
6,0 CT582941-870 F51TW9001CRC89-743
6,1 F51TW9001CAVRU-16
7,3 i04188s126
7,6 i12072s365
11,6 CT575037-832
12,7 BX253509-1186
13,3 F51TW9001EYVPS-834
13,4 F51TW9001EYVPS-968
15,3 m889
17,1 m919
22,1 BX249855-1960
22,2 BX250704-1012
22,5 BX249855-1897
22,6 BX250236-849
23,7 i15859s179
23,8 i15859s14
25,1 F51TW9001B1NYY-663
28,7 CT577936-216
40,6 i09640s629
41,8 AJ309088-966
43,5 i12078s893
49,9 F51TW9001D99E7-1161
52,9 AL750332-783 CT579665-1022
53,0 CT579665-1127
54,4 384_Myb14-449 384_Myb14-4
56,3 m750
70,1 BX254865-791
71,2 F51TW9001AH2IJ-532 F51TW9001AH2IJ-965
77,3 F51TW9001AKK1O-1080
81,3 CT579684-857
81,7 CT577990-1602
81,8 CT579684-197
83,2 CT574744-1983
87,6 BX678298-956
92,1 F51TW9002H6IW7-711
96,6 m1543 m1542
0,0 CT575776-704
3,1 BX255140-610
7,4 F51TW9001AT6H1-1588
11,8 F51TW9001A7FZW-427
17,0 i20560s139 i14110s701
20,0 i11276s420
23,2 i11380s655
23,5 BX249189-569
24,3 i13062s797
25,0 FN695269-96
25,8 i11143s955 BX683041-408
26,3 BX249189-143
28,6 i16267s380
30,3 F51TW9001CWBA6-148
32,1 m891
37,8 F51TW9001BXK1Y-755
39,2 i15225s206
39,4 i15225s502
40,1 AL750570-155
40,2 CT578070-195
44,2 i14027s866
45,0 CT577883-819
49,3 FN695050-209
49,8 m588
55,5 CT575140-1558
55,6 CT575140-541
58,2 F585NNT02GYKMU-438
68,3 BX251376-133
69,8 BX250509-331
70,9 FN696272-518
73,7 CT578169-624
75,3 F51TW9001BCUXW-1272
78,1 CT582330-653
80,9 i15869s643
85,4 i13547s741
86,3 CT580167-200
87,8 AL749753-457
88,1 i18035s88
89,2 BX679946-370
90,1 i09713s1932
90,8 BX255297-556
90,9 BX255297-655
91,0 BX255720-1065
91,1 CT579016-900
GxO
0,0 CT580525-1445 F51TW9001A1S1Z-176
0,6 F51TW9001A1S1Z-350
0,8 FN692129-575
2,8 i17647s443
3,1 i17927s506
3,2 BX253428-2347
3,3 i17927s226
3,5 i17647s350
3,9 BX255599-1948
7,0 F51TW9001CRC89-743 CT582941-870
8,3 i12072s365
8,7 i04188s126
9,1 F51TW9001CAVRU-16
12,9 CT575037-832
14,0 F51TW9001EYVPS-834
14,2 F51TW9001EYVPS-968 BX253509-1186
16,7 m889
23,5 m919
24,1 m920 BX250236-849
CT575572-603 BX254467-915
24,3 CT576042-1032
i11052s1051 i15859s179
25,4 i15859s14
27,7 F51TW9001B1NYY-663
30,9 BX250704-1012
31,2 BX249855-1960 BX249855-1897
31,3 F51TW9001A1611-2347 CT577936-216
32,7 i12078s1070
33,0 i12600s563
33,1 i12600s890
F7JJN6E01A6GI0-158 BX680152-647
33,5 CT580241-1685
41,5 i09640s629
BX678087-77 BX678087-65
42,6 AJ309088-966
49,8 F51TW9001D99E7-1161
49,9 i13850s611
51,1 i13850s692
51,7 BX682611-89
52,5 CT579665-1022 AL750332-783
52,6 CT579665-1127
53,9 384_Myb14-4 384_Myb14-449
55,8 m750
56,0 E34-CATA_49
56,3 BX251981-465
56,7 i11049s1192
57,8 m850
64,2 CT574744-1818
65,2 m929
65,7 F51TW9001A0X21-110 BX680130-2360
66,3 i10330s1327
67,2 CT579684-857 F51TW9001BG2BU-96
67,3 F51TW9001BG2BU-235
68,9 F51TW9001AH2IJ-965
69,1 F51TW9001AH2IJ-532
70,2 F51TW9001EINY8-1225
72,6 CT577310-755
i10928s619 CT577990-1602
76,3 CT579684-197
76,5 F51TW9001AVHMT-574
76,9 i10928s235
77,8 CT574744-1983
78,0 F51TW9001A7FZW-427
83,0 i20560s139 i14110s701
86,3 i11276s420
86,8 BX251174-154
86,9 i05644s593
87,0 BX254087-2185
87,3 i11497s604
89,0 i11380s655
90,0 CT581031-456 i13062s797
90,5 i11143s955
91,1 BX249189-143
91,4 384_CHS-99
92,1 m161
92,7 BX250667-988
92,8 BX250667-917
93,0 i16267s380 F51TW9001CWBA6-148
93,1 BX683041-408
94,2 F51TW9002JSTN9-210
104,6 F51TW9002FIJY9-791 F51TW9002FIJY9-656
106,9 F51TW9001A1TK5-506
108,9 i14027s866
114,6 CT575140-1558
114,7 CT575140-541
115,0 m588
115,7 FN695050-209
117,0 F51TW9001B361N-249
118,5 BX678294-648 BX679702-653
124,6 F51TW9001BCS2D-1156
124,7 F51TW9001BCS2D-1404 AL750269-230
125,4 BX251376-133
128,8 BX250509-331
129,7 FN696272-518
134,0 F51TW9001BCUXW-1272
135,5 FN695650-384
136,9 CT582330-653
139,8 i15869s643
144,2 i13547s741
145,2 CT580167-200
146,7 AL749753-457
147,0 i18035s88
148,1 BX679946-370
149,0 i09713s1932
149,6 BX255297-556
149,8 BX255297-655 BX255720-1065
Oria6
0,0 CT580525-1445 F51TW9001A1S1Z-176
0,1 F51TW9001A1S1Z-350
28,9 m919
BX254467-915 CT575572-603
29,5 CT576042-1032
29,6 m920
30,9 i11052s1051
36,6 F51TW9001A1611-2347
37,4 E34-GATA_80
CT580241-1685 F7JJN6E01A6GI0-158
37,8 BX680152-647
38,1 BX249855-1897 BX249855-1960
38,6 i12600s563
38,7 i12600s890
39,1 i12078s1070
39,7 BX250704-1012
45,8 384_Myb14-527
47,5 BX678087-77 BX678087-65
54,8 i13850s611
56,0 i13850s692
56,4 BX682611-89
60,7 E34-CATA_49
60,8 BX251981-465
61,4 i11049s1192 i17081s436
62,6 m850
68,4 CT574744-1818
69,2 CT579684-857
69,7 m929
69,8 BX680130-2360 F51TW9001A0X21-110
70,4 F51TW9002H6IW7-711
70,7 i10330s1327
71,3 F51TW9001BG2BU-96
71,4 F51TW9001BG2BU-235
74,4 F51TW9001EINY8-1225
76,9 CT577310-755
79,7 CT575776-704
80,6 i10928s619
80,7 F51TW9001AVHMT-574
81,2 i10928s235
91,0 BX251174-154
91,2 i05644s593 BX254087-2185
91,6 i11497s604
94,3 CT581031-456
95,5 384_CHS-99
95,9 i11143s955
96,1 i16267s380
96,5 m161
97,0 BX250667-988 BX250667-917
97,2 BX249189-143 BX683041-408
97,9 F51TW9001CWBA6-148
98,4 F51TW9002JSTN9-210
108,5 F51TW9002FIJY9-656 F51TW9002FIJY9-791
110,6 F51TW9001A1TK5-506
118,8 CT575140-1558 CT575140-541
120,2 F51TW9001B361N-249
121,6 BX678294-648 BX679702-653
125,1 BX251376-133
F51TW9001BCS2D-1156 F51TW9001BCS2D-1404
127,6 AL750269-230
138,6 FN695650-384
139,0 F51TW9001BCUXW-1272
139,5 CT582330-653

LG 3

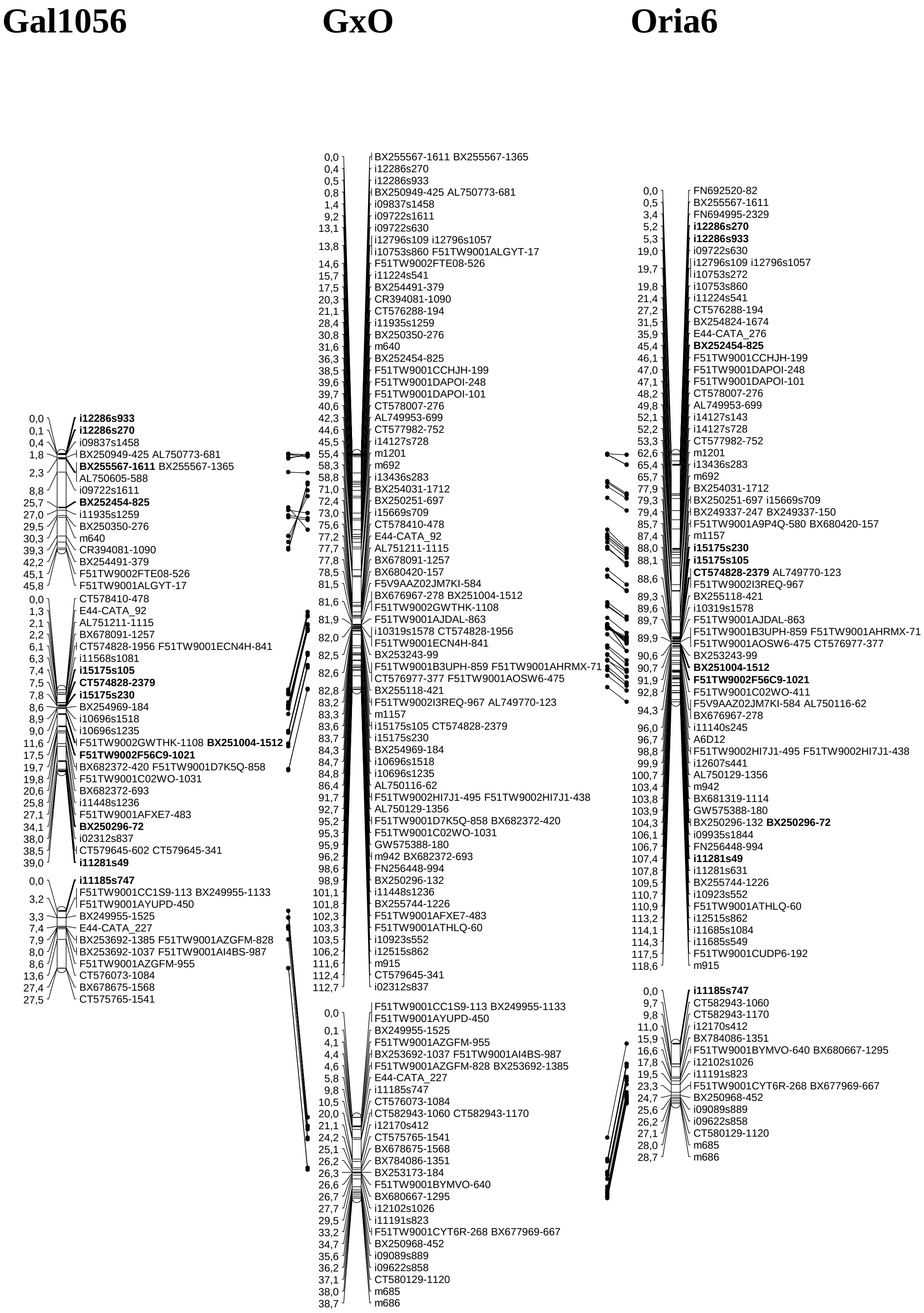
Gal1056
GxO
Oria6
0,0 i12286s933
0,1 i12286s270
0,4 i09837s1458
1,8 BX250949-425 AL750773-681
2,3 BX255567-1611 BX255567-1365
AL750605-588
8,8 i09722s1611
25,7 BX252454-825
27,0 i11935s1259
29,5 BX250350-276
30,3 m640
39,3 CR394081-1090
42,2 BX254491-379
45,1 F51TW9002FTE08-526
45,8 F51TW9001ALGYT-17
0,0 CT578410-478
1,3 E44-CATA_92
2,1 AL751211-1115
2,2 BX678091-1257
6,1 CT574828-1956 F51TW9001ECN4H-841
6,3 i11568s1081
7,4 i15175s105
7,5 CT574828-2379
7,8 i15175s230
8,6 BX254969-184
8,9 i10696s1518
9,0 i10696s1235
11,6 F51TW9002GWTHK-1108 BX251004-1512
17,5 F51TW9002F56C9-1021
19,7 BX682372-420 F51TW9001D7K5Q-858
19,8 F51TW9001C02WO-1031
20,6 BX682372-693
25,8 i11448s1236
27,1 F51TW9001AFXE7-483
34,1 BX250296-72
38,0 i02312s837
38,5 CT579645-602 CT579645-341
39,0 i11281s49
0,0 i11185s747
3,2 F51TW9001CC1S9-113 BX249955-1133
F51TW9001AYUPD-450
3,3 BX249955-1525
7,4 E44-CATA_227
7,9 BX253692-1385 F51TW9001AZGFM-828
8,0 BX253692-1037 F51TW9001AI4BS-987
8,6 F51TW9001AZGFM-955
13,6 CT576073-1084
27,4 BX678675-1568
27,5 CT575765-1541
0,0 BX255567-1611 BX255567-1365
0,4 i12286s270
0,5 i12286s933
0,8 BX250949-425 AL750773-681
1,4 i09837s1458
9,2 i09722s1611
13,1 i09722s630
13,8 i12796s109 i12796s1057
i10753s860 F51TW9001ALGYT-17
14,6 F51TW9002FTE08-526
15,7 i11224s541
17,5 BX254491-379
20,3 CR394081-1090
21,1 CT576288-194
28,4 i11935s1259
30,8 BX250350-276
31,6 m640
36,3 BX252454-825
38,5 F51TW9001CCHJH-199
39,6 F51TW9001DAPOI-248
39,7 F51TW9001DAPOI-101
40,6 CT578007-276
42,3 AL749953-699
44,6 CT577982-752
45,5 i14127s728
55,4 m1201
58,3 m692
58,8 i13436s283
71,0 BX254031-1712
72,4 BX250251-697
73,0 i15669s709
75,6 CT578410-478
77,2 E44-CATA_92
77,7 AL751211-1115
77,8 BX678091-1257
78,5 BX680420-157
81,5 F5V9AAZ02JM7KI-584
81,6 BX676967-278 BX251004-1512
F51TW9002GWTHK-1108
81,9 F51TW9001AJDAL-863
82,0 i10319s1578 CT574828-1956
F51TW9001ECN4H-841
82,5 BX253243-99
82,6 F51TW9001B3UPH-859 F51TW9001AHRMX-71
CT576977-377 F51TW9001AOSW6-475
82,8 BX255118-421
83,2 F51TW9002I3REQ-967 AL749770-123
83,3 m1157
83,6 i15175s105 CT574828-2379
83,7 i15175s230
84,3 BX254969-184
84,7 i10696s1518
84,8 i10696s1235
86,4 AL750116-62
91,7 F51TW9002HI7J1-495 F51TW9002HI7J1-438
92,7 AL750129-1356
95,2 F51TW9001D7K5Q-858 BX682372-420
95,3 F51TW9001C02WO-1031
95,9 GW575388-180
96,2 m942 BX682372-693
98,6 FN256448-994
98,9 BX250296-132
101,1 i11448s1236
101,8 BX255744-1226
102,3 F51TW9001AFXE7-483
103,3 F51TW9001ATHLQ-60
103,5 i10923s552
106,2 i12515s862
111,6 m915
112,4 CT579645-341
112,7 i02312s837
0,0 F51TW9001CC1S9-113 BX249955-1133
F51TW9001AYUPD-450
0,1 BX249955-1525
4,1 F51TW9001AZGFM-955
4,4 BX253692-1037 F51TW9001AI4BS-987
4,6 F51TW9001AZGFM-828 BX253692-1385
5,8 E44-CATA_227
9,8 i11185s747
10,5 CT576073-1084
20,0 CT582943-1060 CT582943-1170
21,1 i12170s412
24,2 CT575765-1541
25,1 BX678675-1568
26,2 BX784086-1351
26,3 BX253173-184
26,6 F51TW9001BYMVO-640
26,7 BX680667-1295
27,7 i12102s1026
29,5 i11191s823
33,2 F51TW9001CYT6R-268 BX677969-667
34,7 BX250968-452
35,6 i09089s889
36,2 i09622s858
37,1 CT580129-1120
38,0 m685
38,7 m686
0,0 FN692520-82
0,5 BX255567-1611
3,4 FN694995-2329
5,2 i12286s270
5,3 i12286s933
19,0 i09722s630
19,7 i12796s109 i12796s1057
i10753s272
19,8 i10753s860
21,4 i11224s541
27,2 CT576288-194
31,5 BX254824-1674
35,9 E44-CATA_276
45,4 BX252454-825
46,1 F51TW9001CCHJH-199
47,0 F51TW9001DAPOI-248
47,1 F51TW9001DAPOI-101
48,2 CT578007-276
49,8 AL749953-699
52,1 i14127s143
52,2 i14127s728
53,3 CT577982-752
62,6 m1201
65,4 i13436s283
65,7 m692
77,9 BX254031-1712
79,3 BX250251-697 i15669s709
79,4 BX249337-247 BX249337-150
85,7 F51TW9001A9P4Q-580 BX680420-157
87,4 m1157
88,0 i15175s230
88,1 i15175s105
88,6 CT574828-2379 AL749770-123
F51TW9002I3REQ-967
89,3 BX255118-421
89,6 i10319s1578
89,7 F51TW9001AJDAL-863
89,9 F51TW9001B3UPH-859 F51TW9001AHRMX-71
F51TW9001AOSW6-475 CT576977-377
90,6 BX253243-99
90,7 BX251004-1512
91,9 F51TW9002F56C9-1021
92,8 F51TW9001C02WO-411
94,3 F5V9AAZ02JM7KI-584 AL750116-62
BX676967-278
96,0 i11140s245
96,7 A6D12
98,8 F51TW9002HI7J1-495 F51TW9002HI7J1-438
99,9 i12607s441
100,7 AL750129-1356
103,4 m942
103,8 BX681319-1114
103,9 GW575388-180
104,3 BX250296-132 BX250296-72
106,1 i09935s1844
106,7 FN256448-994
107,4 i11281s49
107,8 i11281s631
109,5 BX255744-1226
110,7 i10923s552
110,9 F51TW9001ATHLQ-60
113,2 i12515s862
114,1 i11685s1084
114,3 i11685s549
117,5 F51TW9001CUDP6-192
118,6 m915
0,0 i11185s747
9,7 CT582943-1060
9,8 CT582943-1170
11,0 i12170s412
15,9 BX784086-1351
16,6 F51TW9001BYMVO-640 BX680667-1295
17,8 i12102s1026
19,5 i11191s823
23,3 F51TW9001CYT6R-268 BX677969-667
24,7 BX250968-452
25,6 i09089s889
26,2 i09622s858
27,1 CT580129-1120
28,0 m685
28,7 m686

LG 4

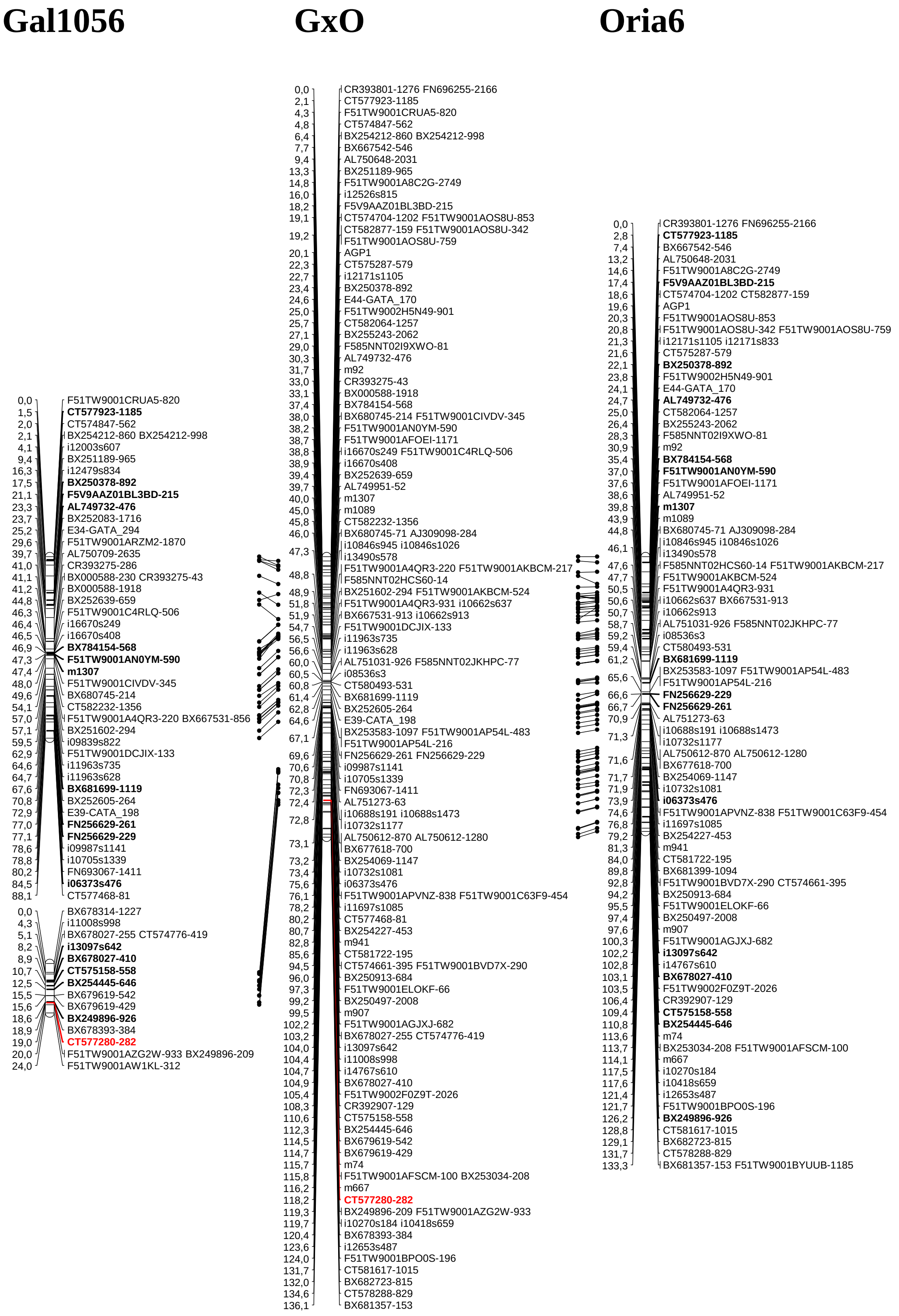

Gal1056
0,0 F51TW9001CRUA5-820
1,5 CT577923-1185
2,0 CT574847-562
2,1 BX254212-860 BX254212-998
4,1 i12003s607
9,4 BX251189-965
16,3 i12479s834
17,5 BX250378-892
21,1 F5V9AAZ01BL3BD-215
23,3 AL749732-476
23,7 BX252083-1716
25,2 E34-GATA_294
29,6 F51TW9001ARZM2-1870
39,7 AL750709-2635
41,0 CR393275-286
41,1 BX000588-230 CR393275-43
41,2 BX000588-1918
44,8 BX252639-659
46,3 F51TW9001C4RLQ-506
46,4 i16670s249
46,5 i16670s408
46,9 BX784154-568
47,3 F51TW9001AN0YM-590
47,4 m1307
48,0 F51TW9001CIVDV-345
49,6 BX680745-214
54,1 CT582232-1356
57,0 F51TW9001A4QR3-220 BX667531-856
57,1 BX251602-294
59,5 i09839s822
62,9 F51TW9001DCJIX-133
64,6 i11963s735
64,7 i11963s628
67,6 BX681699-1119
70,8 BX252605-264
72,9 E39-CATA_198
77,0 FN256629-261
77,1 FN256629-229
78,6 i09987s1141
78,8 i10705s1339
80,2 FN693067-1411
84,5 i06373s476
88,1 CT577468-81
0,0 BX678314-1227
4,3 i11008s998
5,1 BX678027-255 CT574776-419
8,2 i13097s642
8,9 BX678027-410
10,7 CT575158-558
12,5 BX254445-646
15,5 BX679619-542
15,6 BX679619-429
18,6 BX249896-926
18,9 BX678393-384
19,0 CT577280-282
20,0 F51TW9001AZG2W-933 BX249896-209
24,0 F51TW9001AW1KL-312
GxO
0,0 CR393801-1276 FN696255-2166
2,1 CT577923-1185
4,3 F51TW9001CRUA5-820
4,8 CT574847-562
6,4 BX254212-860 BX254212-998
7,7 BX667542-546
9,4 AL750648-2031
13,3 BX251189-965
14,8 F51TW9001A8C2G-2749
16,0 i12526s815
18,2 F5V9AAZ01BL3BD-215
19,1 CT574704-1202 F51TW9001AOS8U-853
19,2 CT582877-159 F51TW9001AOS8U-342
F51TW9001AOS8U-759
20,1 AGP1
22,3 CT575287-579
22,7 i12171s1105
23,4 BX250378-892
24,6 E44-GATA_170
25,0 F51TW9002H5N49-901
25,7 CT582064-1257
27,1 BX255243-2062
29,0 F585NNT02I9XWO-81
30,3 AL749732-476
31,7 m92
33,0 CR393275-43
33,1 BX000588-1918
37,4 BX784154-568
38,0 BX680745-214 F51TW9001CIVDV-345
38,2 F51TW9001AN0YM-590
38,7 F51TW9001AFOEI-1171
38,8 i16670s249 F51TW9001C4RLQ-506
38,9 i16670s408
39,4 BX252639-659
39,7 AL749951-52
40,0 m1307
45,0 m1089
45,8 CT582232-1356
46,0 BX680745-71 AJ309098-284
47,3 i10846s945 i10846s1026
i13490s578
48,8 F51TW9001A4QR3-220 F51TW9001AKBCM-217
F585NNT02HCS60-14
48,9 BX251602-294 F51TW9001AKBCM-524
51,8 F51TW9001A4QR3-931 i10662s637
51,9 BX667531-913 i10662s913
54,7 F51TW9001DCJIX-133
56,5 i11963s735
56,6 i11963s628
60,0 AL751031-926 F585NNT02JKHPC-77
60,5 i08536s3
60,8 CT580493-531
61,4 BX681699-1119
62,8 BX252605-264
64,6 E39-CATA_198
67,1 BX253583-1097 F51TW9001AP54L-483
F51TW9001AP54L-216
69,6 FN256629-261 FN256629-229
70,6 i09987s1141
70,8 i10705s1339
72,3 FN693067-1411
72,4 AL751273-63
72,8 i10688s191 i10688s1473
i10732s1177
73,1 AL750612-870 AL750612-1280
BX677618-700
73,2 BX254069-1147
73,4 i10732s1081
75,6 i06373s476
76,1 F51TW9001APVNZ-838 F51TW9001C63F9-454
78,2 i11697s1085
80,2 CT577468-81
80,7 BX254227-453
82,8 m941
85,6 CT581722-195
94,5 CT574661-395 F51TW9001BVD7X-290
96,0 BX250913-684
97,3 F51TW9001ELOKF-66
99,2 BX250497-2008
99,5 m907
102,2 F51TW9001AGJXJ-682
103,2 BX678027-255 CT574776-419
104,0 i13097s642
104,4 i11008s998
104,7 i14767s610
104,9 BX678027-410
105,4 F51TW9002F0Z9T-2026
108,3 CR392907-129
110,6 CT575158-558
112,3 BX254445-646
114,5 BX679619-542
114,7 BX679619-429
115,7 m74
115,8 F51TW9001AFSCM-100 BX253034-208
116,2 m667
118,2 CT577280-282
119,3 BX249896-209 F51TW9001AZG2W-933
119,7 i10270s184 i10418s659
120,4 BX678393-384
123,6 i12653s487
124,0 F51TW9001BPO0S-196
131,7 CT581617-1015
132,0 BX682723-815
134,6 CT578288-829
136,1 BX681357-153
Oria6
0,0 CR393801-1276 FN696255-2166
2,8 CT577923-1185
7,4 BX667542-546
13,2 AL750648-2031
14,6 F51TW9001A8C2G-2749
17,4 F5V9AAZ01BL3BD-215
18,6 CT574704-1202 CT582877-159
19,6 AGP1
20,3 F51TW9001AOS8U-853
20,8 F51TW9001AOS8U-342 F51TW9001AOS8U-759
21,3 i12171s1105 i12171s833
21,6 CT575287-579
22,1 BX250378-892
23,8 F51TW9002H5N49-901
24,1 E44-GATA_170
24,7 AL749732-476
25,0 CT582064-1257
26,4 BX255243-2062
28,3 F585NNT02I9XWO-81
30,9 m92
35,4 BX784154-568
37,0 F51TW9001AN0YM-590
37,6 F51TW9001AFOEI-1171
38,6 AL749951-52
39,8 m1307
43,9 m1089
44,8 BX680745-71 AJ309098-284
46,1 i10846s945 i10846s1026
i13490s578
47,6 F585NNT02HCS60-14 F51TW9001AKBCM-217
47,7 F51TW9001AKBCM-524
50,5 F51TW9001A4QR3-931
50,6 i10662s637 BX667531-913
50,7 i10662s913
58,7 AL751031-926 F585NNT02JKHPC-77
59,2 i08536s3
59,4 CT580493-531
61,2 BX681699-1119
65,6 BX253583-1097 F51TW9001AP54L-483
F51TW9001AP54L-216
66,6 FN256629-229
66,7 FN256629-261
70,9 AL751273-63
71,3 i10688s191 i10688s1473
i10732s1177
71,6 AL750612-870 AL750612-1280
BX677618-700
71,7 BX254069-1147
71,9 i10732s1081
73,9 i06373s476
74,6 F51TW9001APVNZ-838 F51TW9001C63F9-454
76,8 i11697s1085
79,2 BX254227-453
81,3 m941
84,0 CT581722-195
89,8 BX681399-1094
92,8 F51TW9001BVD7X-290 CT574661-395
94,2 BX250913-684
95,5 F51TW9001ELOKF-66
97,4 BX250497-2008
97,6 m907
100,3 F51TW9001AGJXJ-682
102,2 i13097s642
102,8 i14767s610
103,1 BX678027-410
103,5 F51TW9002F0Z9T-2026
106,4 CR392907-129
109,4 CT575158-558
110,8 BX254445-646
113,6 m74
113,7 BX253034-208 F51TW9001AFSCM-100
114,1 m667
117,5 i10270s184
117,6 i10418s659
121,4 i12653s487
121,7 F51TW9001BPO0S-196
126,2 BX249896-926
128,8 CT581617-1015
129,1 BX682723-815
131,7 CT578288-829
133,3 BX681357-153 F51TW9001BYUUB-1185

LG 5

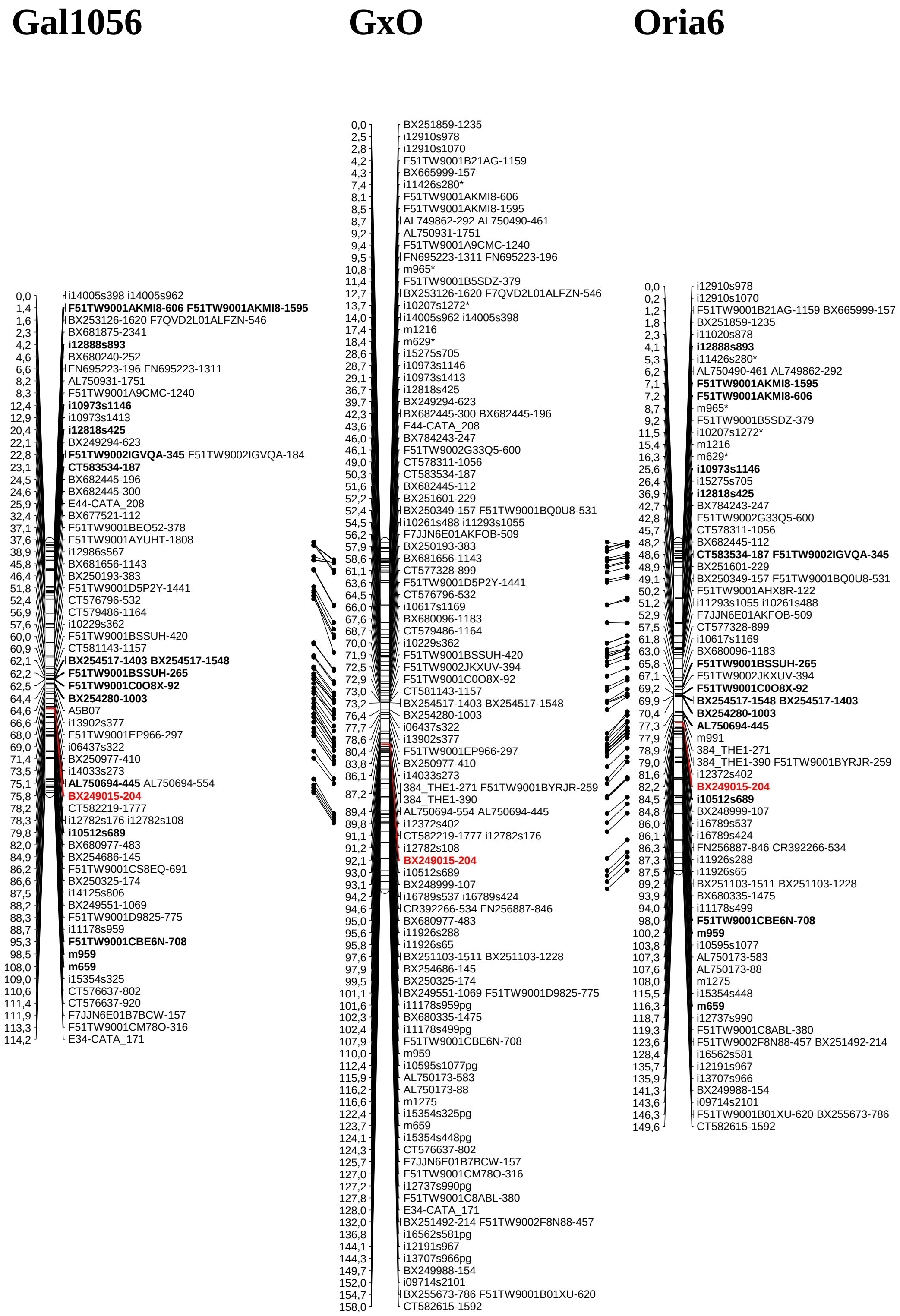
Gal1056
0,0 i14005s398 i14005s962
1,4 F51TW9001AKMI8-606 F51TW9001AKMI8-1595
1,6 BX253126-1620 F7QVD2L01ALFZN-546
2,3 BX681875-2341
4,2 i12888s893
4,6 BX680240-252
6,6 FN695223-196 FN695223-1311
8,2 AL750931-1751
8,3 F51TW9001A9CMC-1240
12,4 i10973s1146
12,9 i10973s1413
20,4 i12818s425
22,1 BX249294-623
22,8 F51TW9002IGVQA-345 F51TW9002IGVQA-184
23,1 CT583534-187
24,5 BX682445-196
24,6 BX682445-300
25,9 E44-CATA_208
32,4 BX677521-112
37,1 F51TW9001BEO52-378
37,6 F51TW9001AYUHT-1808
38,9 i12986s567
45,8 BX681656-1143
46,4 BX250193-383
51,8 F51TW9001D5P2Y-1441
52,4 CT576796-532
56,9 CT579486-1164
57,6 i10229s362
60,0 F51TW9001BSSUH-420
60,9 CT581143-1157
62,1 BX254517-1403 BX254517-1548
62,2 F51TW9001BSSUH-265
62,5 F51TW9001C0O8X-92
64,4 BX254280-1003
64,6 A5B07
66,6 i13902s377
68,0 F51TW9001EP966-297
69,0 i06437s322
71,4 BX250977-410
73,5 i14033s273
75,1 AL750694-445 AL750694-554
75,8 BX249015-204
78,2 CT582219-1777
78,3 i12782s176 i12782s108
79,8 i10512s689
82,0 BX680977-483
84,9 BX254686-145
86,2 F51TW9001CS8EQ-691
86,6 BX250325-174
87,5 i14125s806
88,2 BX249551-1069
88,3 F51TW9001D9825-775
88,7 i11178s959
95,3 F51TW9001CBE6N-708
98,5 m959
108,0 m659
109,0 i15354s325
110,6 CT576637-802
111,4 CT576637-920
111,9 F7JJN6E01B7BCW-157
113,3 F51TW9001CM78O-316
114,2 E34-CATA_171
GxO
0,0 BX251859-1235
2,5 i12910s978
2,8 i12910s1070
4,2 F51TW9001B21AG-1159
4,3 BX665999-157
7,4 i11426s280*
8,1 F51TW9001AKMI8-606
8,5 F51TW9001AKMI8-1595
8,7 AL749862-292 AL750490-461
9,2 AL750931-1751
9,4 F51TW9001A9CMC-1240
9,5 FN695223-1311 FN695223-196
10,8 m965*
11,4 F51TW9001B5SDZ-379
12,7 BX253126-1620 F7QVD2L01ALFZN-546
13,7 i10207s1272*
14,0 i14005s962 i14005s398
17,4 m1216
18,4 m629*
28,6 i15275s705
28,7 i10973s1146
29,1 i10973s1413
36,7 i12818s425
39,7 BX249294-623
42,3 BX682445-300 BX682445-196
43,6 E44-CATA_208
46,0 BX784243-247
46,1 F51TW9002G33Q5-600
49,0 CT578311-1056
50,3 CT583534-187
51,6 BX682445-112
52,2 BX251601-229
52,4 BX250349-157 F51TW9001BQ0U8-531
54,5 i10261s488 i11293s1055
56,2 F7JJN6E01AKFOB-509
57,9 BX250193-383
58,6 BX681656-1143
61,1 CT577328-899
63,6 F51TW9001D5P2Y-1441
64,5 CT576796-532
66,0 i10617s1169
67,6 BX680096-1183
68,7 CT579486-1164
70,0 i10229s362
71,9 F51TW9001BSSUH-420
72,5 F51TW9002JKXUV-394
72,9 F51TW9001C0O8X-92
73,0 CT581143-1157
73,2 BX254517-1403 BX254517-1548
76,4 BX254280-1003
77,7 i06437s322
78,6 i13902s377
80,4 F51TW9001EP966-297
83,8 BX250977-410
86,1 i14033s273
87,2 384_THE1-271 F51TW9001BYRJR-259
384_THE1-390
89,4 AL750694-554 AL750694-445
89,8 i12372s402
91,1 CT582219-1777 i12782s176
91,2 i12782s108
92,1 BX249015-204
93,0 i10512s689
93,1 BX248999-107
94,2 i16789s537 i16789s424
94,6 CR392266-534 FN256887-846
95,0 BX680977-483
95,6 i11926s288
95,8 i11926s65
97,6 BX251103-1511 BX251103-1228
97,9 BX254686-145
99,5 BX250325-174
101,1 BX249551-1069 F51TW9001D9825-775
101,6 i11178s959pg
102,3 BX680335-1475
102,4 i11178s499pg
107,9 F51TW9001CBE6N-708
110,0 m959
112,4 i10595s1077pg
115,9 AL750173-583
116,2 AL750173-88
116,6 m1275
122,4 i15354s325pg
123,7 m659
124,1 i15354s448pg
124,3 CT576637-802
125,7 F7JJN6E01B7BCW-157
127,0 F51TW9001CM78O-316
127,2 i12737s990pg
127,8 F51TW9001C8ABL-380
128,0 E34-CATA_171
132,0 BX251492-214 F51TW9002F8N88-457
136,8 i16562s581pg
144,1 i12191s967
144,3 i13707s966pg
149,7 BX249988-154
152,0 i09714s2101
154,7 BX255673-786 F51TW9001B01XU-620
158,0 CT582615-1592
Oria6
0,0 i12910s978
0,2 i12910s1070
1,2 F51TW9001B21AG-1159 BX665999-157
1,8 BX251859-1235
2,3 i11020s878
4,1 i12888s893
5,3 i11426s280*
6,2 AL750490-461 AL749862-292
7,1 F51TW9001AKMI8-1595
7,2 F51TW9001AKMI8-606
8,7 m965*
9,2 F51TW9001B5SDZ-379
11,5 i10207s1272*
15,4 m1216
16,3 m629*
25,6 i10973s1146
26,4 i15275s705
36,9 i12818s425
42,7 BX784243-247
42,8 F51TW9002G33Q5-600
45,7 CT578311-1056
48,2 BX682445-112
48,6 CT583534-187 F51TW9002IGVQA-345
48,9 BX251601-229
49,1 BX250349-157 F51TW9001BQ0U8-531
50,2 F51TW9001AHX8R-122
51,2 i11293s1055 i10261s488
52,9 F7JJN6E01AKFOB-509
57,5 CT577328-899
61,8 i10617s1169
63,0 BX680096-1183
65,8 F51TW9001BSSUH-265
67,1 F51TW9002JKXUV-394
69,2 F51TW9001C0O8X-92
69,9 BX254517-1548 BX254517-1403
70,4 BX254280-1003
77,3 AL750694-445
77,9 m991
78,9 384_THE1-271
79,0 384_THE1-390 F51TW9001BYRJR-259
81,6 i12372s402
82,2 BX249015-204
84,5 i10512s689
84,8 BX248999-107
86,0 i16789s537
86,1 i16789s424
86,3 FN256887-846 CR392266-534
87,3 i11926s288
87,5 i11926s65
89,2 BX251103-1511 BX251103-1228
93,9 BX680335-1475
94,0 i11178s499
98,0 F51TW9001CBE6N-708
100,2 m959
103,8 i10595s1077
107,3 AL750173-583
107,6 AL750173-88
108,0 m1275
115,5 i15354s448
116,3 m659
118,7 i12737s990
119,3 F51TW9001C8ABL-380
123,6 F51TW9002F8N88-457 BX251492-214
128,4 i16562s581
135,7 i12191s967
135,9 i13707s966
141,3 BX249988-154
143,6 i09714s2101
146,3 F51TW9001B01XU-620 BX255673-786
149,6 CT582615-1592

LG 6

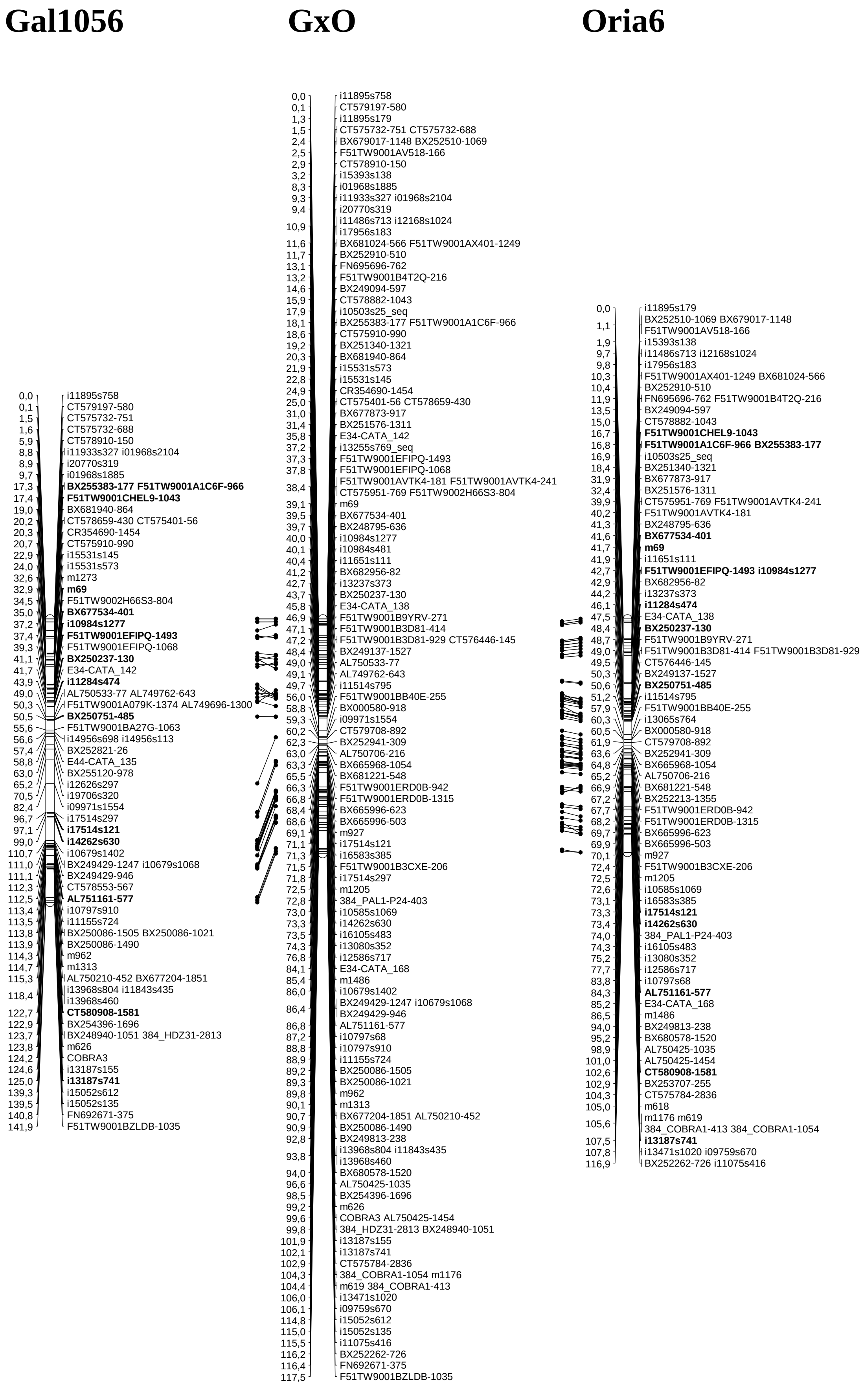

Gal1056
0,0 i11895s758
0,1 CT579197-580
1,5 CT575732-751
1,6 CT575732-688
5,9 CT578910-150
8,8 i11933s327 i01968s2104
8,9 i20770s319
9,7 i01968s1885
17,3 BX255383-177 F51TW9001A1C6F-966
17,4 F51TW9001CHEL9-1043
19,0 BX681940-864
20,2 CT578659-430 CT575401-56
20,3 CR354690-1454
20,7 CT575910-990
22,9 i15531s145
24,0 i15531s573
32,6 m1273
32,9 m69
34,5 F51TW9002H66S3-804
35,0 BX677534-401
37,2 i10984s1277
37,4 F51TW9001EFIPQ-1493
39,3 F51TW9001EFIPQ-1068
41,1 BX250237-130
41,7 E34-CATA_142
43,9 i11284s474
49,0 AL750533-77 AL749762-643
50,3 F51TW9001A079K-1374 AL749696-1300
50,5 BX250751-485
55,6 F51TW9001BA27G-1063
56,6 i14956s698 i14956s113
57,4 BX252821-26
58,8 E44-CATA_135
63,0 BX255120-978
65,2 i12626s297
70,5 i19706s320
82,4 i09971s1554
96,7 i17514s297
97,1 i17514s121
99,0 i14262s630
110,7 i10679s1402
111,0 BX249429-1247 i10679s1068
111,1 BX249429-946
112,3 CT578553-567
112,5 AL751161-577
113,4 i10797s910
113,5 i11155s724
113,8 BX250086-1505 BX250086-1021
113,9 BX250086-1490
114,3 m962
114,7 m1313
115,3 AL750210-452 BX677204-1851
118,4 i13968s804 i11843s435
i13968s460
122,7 CT580908-1581
122,9 BX254396-1696
123,7 BX248940-1051 384_HDZ31-2813
123,8 m626
124,2 COBRA3
124,6 i13187s155
125,0 i13187s741
139,3 i15052s612
139,5 i15052s135
140,8 FN692671-375
141,9 F51TW9001BZLDB-1035
GxO
0,0 i11895s758
0,1 CT579197-580
1,3 i11895s179
1,5 CT575732-751 CT575732-688
2,4 BX679017-1148 BX252510-1069
2,5 F51TW9001AV518-166
2,9 CT578910-150
3,2 i15393s138
8,3 i01968s1885
9,3 i11933s327 i01968s2104
9,4 i20770s319
10,9 i11486s713 i12168s1024
i17956s183
11,6 BX681024-566 F51TW9001AX401-1249
11,7 BX252910-510
13,1 FN695696-762
13,2 F51TW9001B4T2Q-216
14,6 BX249094-597
15,9 CT578882-1043
17,9 i10503s25_seq
18,1 BX255383-177 F51TW9001A1C6F-966
18,6 CT575910-990
19,2 BX251340-1321
20,3 BX681940-864
21,9 i15531s573
22,8 i15531s145
24,9 CR354690-1454
25,0 CT575401-56 CT578659-430
31,0 BX677873-917
31,4 BX251576-1311
35,8 E34-CATA_142
37,2 i13255s769_seq
37,3 F51TW9001EFIPQ-1493
37,8 F51TW9001EFIPQ-1068
38,4 F51TW9001AVTK4-181 F51TW9001AVTK4-241
CT575951-769 F51TW9002H66S3-804
39,1 m69
39,5 BX677534-401
39,7 BX248795-636
40,0 i10984s1277
40,1 i10984s481
40,4 i11651s111
41,2 BX682956-82
42,7 i13237s373
43,7 BX250237-130
45,8 E34-CATA_138
46,9 F51TW9001B9YRV-271
47,1 F51TW9001B3D81-414
47,2 F51TW9001B3D81-929 CT576446-145
48,4 BX249137-1527
49,0 AL750533-77
49,1 AL749762-643
49,7 i11514s795
56,0 F51TW9001BB40E-255
58,8 BX000580-918
59,3 i09971s1554
60,2 CT579708-892
62,3 BX252941-309
63,0 AL750706-216
63,3 BX665968-1054
65,5 BX681221-548
66,3 F51TW9001ERD0B-942
66,8 F51TW9001ERD0B-1315
68,4 BX665996-623
68,6 BX665996-503
69,1 m927
71,1 i17514s121
71,3 i16583s385
71,5 F51TW9001B3CXE-206
71,8 i17514s297
72,5 m1205
72,8 384_PAL1-P24-403
73,0 i10585s1069
73,3 i14262s630
73,5 i16105s483
74,3 i13080s352
76,8 i12586s717
84,1 E34-CATA_168
85,4 m1486
86,0 i10679s1402
86,4 BX249429-1247 i10679s1068
BX249429-946
86,8 AL751161-577
87,2 i10797s68
88,8 i10797s910
88,9 i11155s724
89,2 BX250086-1505
89,3 BX250086-1021
89,8 m962
90,1 m1313
90,7 BX677204-1851 AL750210-452
90,9 BX250086-1490
92,8 BX249813-238
93,8 i13968s804 i11843s435
i13968s460
94,0 BX680578-1520
96,6 AL750425-1035
98,5 BX254396-1696
99,2 m626
99,6 COBRA3 AL750425-1454
99,8 384_HDZ31-2813 BX248940-1051
101,9 i13187s155
102,1 i13187s741
102,9 CT575784-2836
104,3 384_COBRA1-1054 m1176
104,4 m619 384_COBRA1-413
106,0 i13471s1020
106,1 i09759s670
114,8 i15052s612
115,0 i15052s135
115,5 i11075s416
116,2 BX252262-726
116,4 FN692671-375
117,5 F51TW9001BZLDB-1035
Oria6
0,0 i11895s179
1,1 BX252510-1069 BX679017-1148
F51TW9001AV518-166
1,9 i15393s138
9,7 i11486s713 i12168s1024
9,8 i17956s183
10,3 F51TW9001AX401-1249 BX681024-566
10,4 BX252910-510
11,9 FN695696-762 F51TW9001B4T2Q-216
13,5 BX249094-597
15,0 CT578882-1043
16,7 F51TW9001CHEL9-1043
16,8 F51TW9001A1C6F-966 BX255383-177
16,9 i10503s25_seq
18,4 BX251340-1321
31,9 BX677873-917
32,4 BX251576-1311
39,9 CT575951-769 F51TW9001AVTK4-241
40,2 F51TW9001AVTK4-181
41,3 BX248795-636
41,6 BX677534-401
41,7 m69
41,9 i11651s111
42,7 F51TW9001EFIPQ-1493 i10984s1277
42,9 BX682956-82
44,2 i13237s373
46,1 i11284s474
47,5 E34-CATA_138
48,4 BX250237-130
48,7 F51TW9001B9YRV-271
49,0 F51TW9001B3D81-414 F51TW9001B3D81-929
49,5 CT576446-145
50,3 BX249137-1527
50,6 BX250751-485
51,2 i11514s795
57,9 F51TW9001BB40E-255
60,3 i13065s764
60,5 BX000580-918
61,9 CT579708-892
63,6 BX252941-309
64,8 BX665968-1054
65,2 AL750706-216
66,9 BX681221-548
67,2 BX252213-1355
67,7 F51TW9001ERD0B-942
68,2 F51TW9001ERD0B-1315
69,7 BX665996-623
69,9 BX665996-503
70,1 m927
72,4 F51TW9001B3CXE-206
72,5 m1205
72,6 i10585s1069
73,1 i16583s385
73,3 i17514s121
73,4 i14262s630
74,0 384_PAL1-P24-403
74,3 i16105s483
75,2 i13080s352
77,7 i12586s717
83,8 i10797s68
84,3 AL751161-577
85,2 E34-CATA_168
86,5 m1486
94,0 BX249813-238
95,2 BX680578-1520
98,9 AL750425-1035
101,0 AL750425-1454
102,6 CT580908-1581
102,9 BX253707-255
104,3 CT575784-2836
105,0 m618
105,6 m1176 m619
384_COBRA1-413 384_COBRA1-1054
107,5 i13187s741
107,8 i13471s1020 i09759s670
116,9 BX252262-726 i11075s416

LG 7

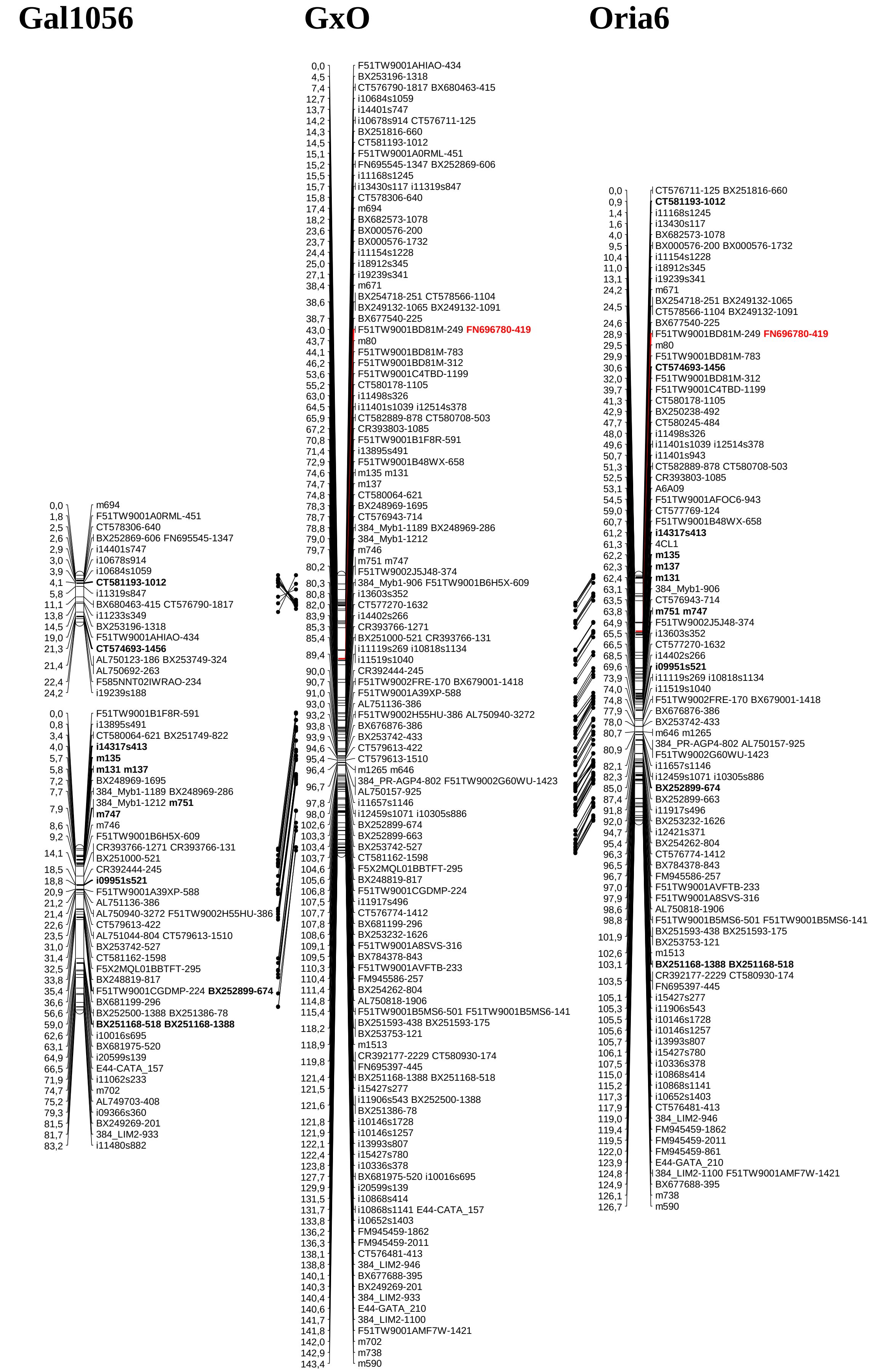

Gal1056
0,0
1,8
2,5
2,6
2,9
3,0
3,9
4,1
5,8
11,1
13,8
14,5
19,0
21,3
21,4
22,4
24,2
m694
F51TW9001A0RML-451
CT578306-640
BX252869-606 FN695545-1347
i14401s747
i10678s914
i10684s1059
CT581193-1012
i11319s847
BX680463-415 CT576790-1817
i11233s349
BX253196-1318
F51TW9001AHIAO-434
CT574693-1456
AL750123-186 BX253749-324
AL750692-263
F585NNT02IWRAO-234
i19239s188
0,0
0,8
3,4
4,0
5,7
5,8
7,2
7,7
7,9
8,6
9,2
14,1
18,5
18,8
20,9
21,2
21,4
22,6
23,5
31,0
31,4
32,5
33,8
35,4
36,6
56,6
59,0
62,6
63,1
64,9
66,5
71,9
74,7
75,2
79,3
81,5
81,7
83,2
F51TW9001B1F8R-591
i13895s491
CT580064-621 BX251749-822
i14317s413
m135
m131 m137
BX248969-1695
384_Myb1-1189 BX248969-286
384_Myb1-1212 m751
m747
m746
F51TW9001B6H5X-609
CR393766-1271 CR393766-131
BX251000-521
CR392444-245
i09951s521
F51TW9001A39XP-588
AL751136-386
AL750940-3272 F51TW9002H55HU-386
CT579613-422
AL751044-804 CT579613-1510
BX253742-527
CT581162-1598
F5X2MQL01BBTFT-295
BX248819-817
F51TW9001CGDMP-224 BX252899-674
BX681199-296
BX252500-1388 BX251386-78
BX251168-518 BX251168-1388
i10016s695
BX681975-520
i20599s139
E44-CATA_157
i11062s233
m702
AL749703-408
i09366s360
BX249269-201
384_LIM2-933
i11480s882
GxO
0,0
4,5
7,4
12,7
13,7
14,2
14,3
14,5
15,1
15,2
15,5
15,7
15,8
17,4
18,2
23,6
23,7
24,4
25,0
27,1
38,4
38,6
38,7
43,0
43,7
44,1
46,2
53,6
55,2
63,0
64,5
65,9
67,2
70,8
71,4
72,9
74,6
74,7
74,8
78,3
78,7
78,8
79,0
79,7
80,2
80,3
80,8
82,0
83,9
85,3
85,4
89,4
90,0
90,7
91,0
93,0
93,2
93,8
93,9
94,6
95,4
96,4
96,7
97,8
98,0
102,6
103,3
103,4
103,7
104,6
105,6
106,8
107,5
107,7
107,8
108,6
109,1
109,5
110,3
110,4
111,4
114,8
115,4
118,2
118,9
119,8
121,4
121,5
121,6
121,8
121,9
122,1
122,4
123,8
127,7
129,9
131,5
131,7
133,8
136,2
136,3
138,1
138,8
140,1
140,3
140,4
140,6
141,7
141,8
142,0
142,9
143,4
F51TW9001AHIAO-434
BX253196-1318
CT576790-1817 BX680463-415
i10684s1059
i14401s747
i10678s914 CT576711-125
BX251816-660
CT581193-1012
F51TW9001A0RML-451
FN695545-1347 BX252869-606
i11168s1245
i13430s117 i11319s847
CT578306-640
m694
BX682573-1078
BX000576-200
BX000576-1732
i11154s1228
i18912s345
i19239s341
m671
BX254718-251 CT578566-1104
BX249132-1065 BX249132-1091
BX677540-225
F51TW9001BD81M-249 FN696780-419
m80
F51TW9001BD81M-783
F51TW9001BD81M-312
F51TW9001C4TBD-1199
CT580178-1105
i11498s326
i11401s1039 i12514s378
CT582889-878 CT580708-503
CR393803-1085
F51TW9001B1F8R-591
i13895s491
F51TW9001B48WX-658
m135 m131
m137
CT580064-621
BX248969-1695
CT576943-714
384_Myb1-1189 BX248969-286
384_Myb1-1212
m746
m751 m747
F51TW9002J5J48-374
384_Myb1-906 F51TW9001B6H5X-609
i13603s352
CT577270-1632
i14402s266
CR393766-1271
BX251000-521 CR393766-131
i11119s269 i10818s1134
i11519s1040
CR392444-245
F51TW9002FRE-170 BX679001-1418
F51TW9001A39XP-588
AL751136-386
F51TW9002H55HU-386 AL750940-3272
BX676876-386
BX253742-433
CT579613-422
CT579613-1510
m1265 m646
384_PR-AGP4-802 F51TW9002G60WU-1423
AL750157-925
i11657s1146
i12459s1071 i10305s886
BX252899-674
BX252899-663
BX253742-527
CT581162-1598
F5X2MQL01BBTFT-295
BX248819-817
F51TW9001CGDMP-224
i11917s496
CT576774-1412
BX681199-296
BX253232-1626
F51TW9001A8SVS-316
BX784378-843
F51TW9001AVFTB-233
FM945586-257
BX254262-804
AL750818-1906
F51TW9001B5MS6-501 F51TW9001B5MS6-141
BX251593-438 BX251593-175
BX253753-121
m1513
CR392177-2229 CT580930-174
FN695397-445
BX251168-1388 BX251168-518
i15427s277
i11906s543 BX252500-1388
BX251386-78
i10146s1728
i10146s1257
i13993s807
i15427s780
i10336s378
BX681975-520 i10016s695
i20599s139
i10868s414
i10868s1141 E44-CATA_157
i10652s1403
FM945459-1862
FM945459-2011
CT576481-413
384_LIM2-946
BX677688-395
BX249269-201
384_LIM2-933
E44-GATA_210
384_LIM2-1100
F51TW9001AMF7W-1421
m702
m738
m590
Oria6
0,0
0,9
1,4
1,6
4,0
9,5
10,4
11,0
13,1
24,2
24,5
24,6
28,9
29,5
29,9
30,6
32,0
39,7
41,3
42,9
47,7
48,0
49,6
50,7
51,3
52,5
53,1
54,5
59,0
60,7
61,2
61,3
62,2
62,3
62,4
63,1
63,5
63,8
64,9
65,5
66,5
68,5
69,6
73,9
74,0
74,8
77,9
78,0
80,7
80,9
82,1
82,3
85,0
87,4
91,8
92,0
94,7
95,4
96,3
96,5
96,7
97,0
97,9
98,6
98,8
101,9
102,6
103,1
103,5
105,1
105,3
105,5
105,6
105,7
106,1
107,5
115,0
115,2
117,3
117,9
119,0
119,4
119,5
122,0
123,9
124,8
124,9
126,1
126,7
CT576711-125 BX251816-660
CT581193-1012
i11168s1245
i13430s117
BX682573-1078
BX000576-200 BX000576-1732
i11154s1228
i18912s345
i19239s341
m671
BX254718-251 BX249132-1065
CT578566-1104 BX249132-1091
BX677540-225
F51TW9001BD81M-249 FN696780-419
m80
F51TW9001BD81M-783
CT574693-1456
F51TW9001BD81M-312
F51TW9001C4TBD-1199
CT580178-1105
BX250238-492
CT580245-484
i11498s326
i11401s1039 i12514s378
i11401s943
CT582889-878 CT580708-503
CR393803-1085
A6A09
F51TW9001AFOC6-943
CT577769-124
F51TW9001B48WX-658
i14317s413
4CL1
m135
m137
m131
384_Myb1-906
CT576943-714
m751 m747
F51TW9002J5J48-374
i13603s352
CT577270-1632
i14402s266
i09951s521
i11119s269 i10818s1134
i11519s1040
F51TW9002FRE-170 BX679001-1418
BX676876-386
BX253742-433
m646 m1265
384_PR-AGP4-802 AL750157-925
F51TW9002G60WU-1423
i11657s1146
i12459s1071 i10305s886
BX252899-674
BX252899-663
i11917s496
BX253232-1626
i12421s371
BX254262-804
CT576774-1412
BX784378-843
FM945586-257
F51TW9001AVFTB-233
F51TW9001A8SVS-316
AL750818-1906
F51TW9001B5MS6-501 F51TW9001B5MS6-141
BX251593-438 BX251593-175
BX253753-121
m1513
BX251168-1388 BX251168-518
CR392177-2229 CT580930-174
FN695397-445
i15427s277
i11906s543
i10146s1728
i10146s1257
i13993s807
i15427s780
i10336s378
i10868s414
i10868s1141
i10652s1403
CT576481-413
384_LIM2-946
FM945459-1862
FM945459-2011
FM945459-861
E44-GATA_210
384_LIM2-1100 F51TW9001AMF7W-1421
BX677688-395
m738
m590

LG 8

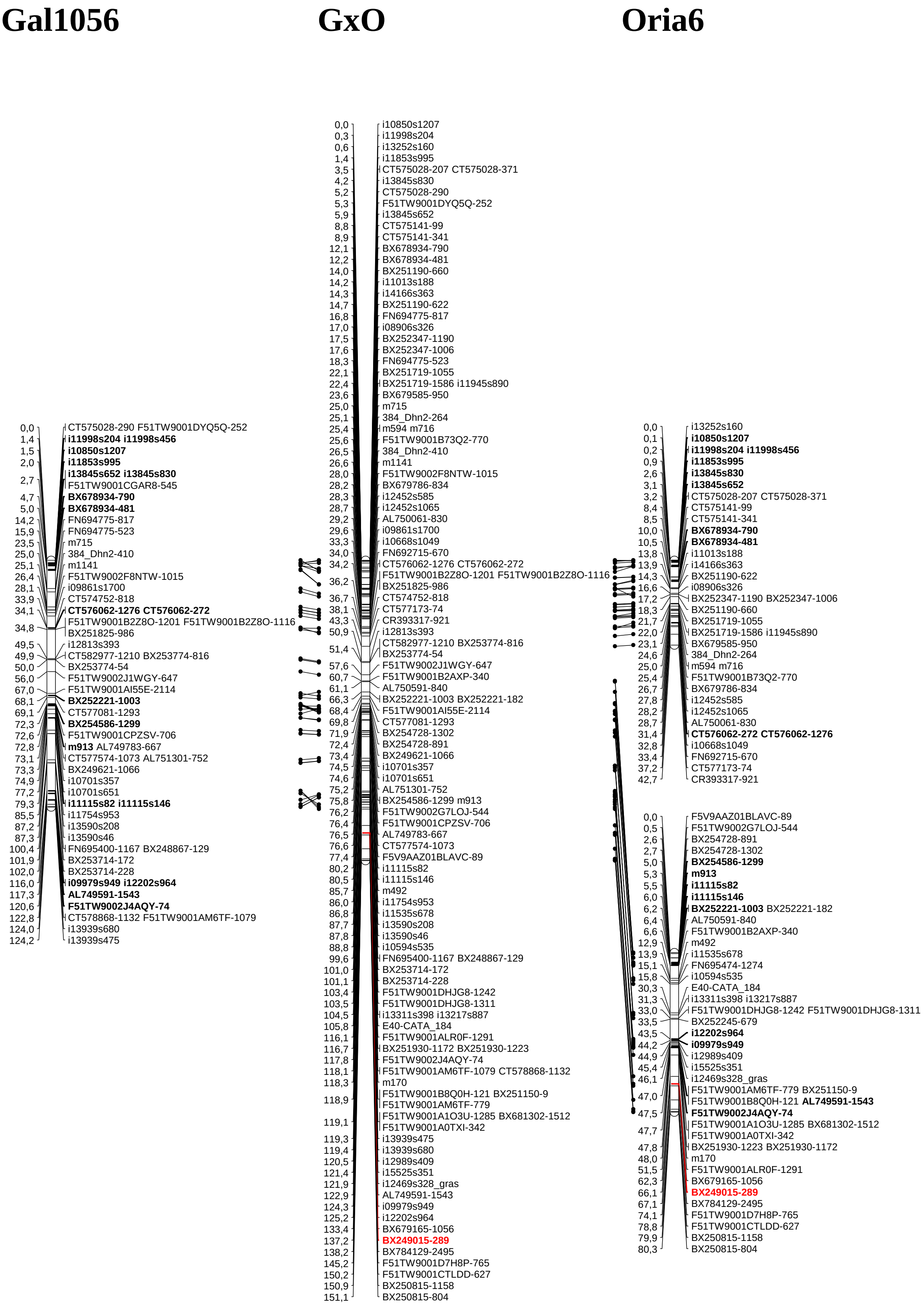

Gal1056
GxO
Oria6
0,0 CT575028-290 F51TW9001DYQ5Q-252
1,4 i11998s204 i11998s456
1,5 i10850s1207
2,0 i11853s995
2,7 i13845s652 i13845s830
F51TW9001CGAR8-545
4,7 BX678934-790
5,0 BX678934-481
14,2 FN694775-817
15,9 FN694775-523
23,5 m715
25,0 384_Dhn2-410
25,1 m1141
26,4 F51TW9002F8NTW-1015
28,1 i09861s1700
33,9 CT574752-818
34,1 CT576062-1276 CT576062-272
34,8 F51TW9001B2Z8O-1201 F51TW9001B2Z8O-1116
BX251825-986
49,5 i12813s393
49,9 CT582977-1210 BX253774-816
50,0 BX253774-54
56,0 F51TW9002J1WGY-647
67,0 F51TW9001AI55E-2114
68,1 BX252221-1003
69,1 CT577081-1293
72,3 BX254586-1299
72,6 F51TW9001CPZSV-706
72,8 m913 AL749783-667
73,1 CT577574-1073 AL751301-752
73,3 BX249621-1066
74,9 i10701s357
77,2 i10701s651
79,3 i11115s82 i11115s146
85,5 i11754s953
87,2 i13590s208
87,3 i13590s46
100,4 FN695400-1167 BX248867-129
101,9 BX253714-172
102,0 BX253714-228
116,0 i09979s949 i12202s964
117,3 AL749591-1543
120,6 F51TW9002J4AQY-74
122,8 CT578868-1132 F51TW9001AM6TF-1079
124,0 i13939s680
124,2 i13939s475
0,0 i10850s1207
0,3 i11998s204
0,6 i13252s160
1,4 i11853s995
3,5 CT575028-207 CT575028-371
4,2 i13845s830
5,2 CT575028-290
5,3 F51TW9001DYQ5Q-252
5,9 i13845s652
8,8 CT575141-99
8,9 CT575141-341
12,1 BX678934-790
12,2 BX678934-481
14,0 BX251190-660
14,2 i11013s188
14,3 i14166s363
14,7 BX251190-622
16,8 FN694775-817
17,0 i08906s326
17,5 BX252347-1190
17,6 BX252347-1006
18,3 FN694775-523
22,1 BX251719-1055
22,4 BX251719-1586 i11945s890
23,6 BX679585-950
25,0 m715
25,1 384_Dhn2-264
25,4 m594 m716
25,6 F51TW9001B73Q2-770
26,5 384_Dhn2-410
26,6 m1141
28,0 F51TW9002F8NTW-1015
28,2 BX679786-834
28,3 i12452s585
28,7 i12452s1065
29,2 AL750061-830
29,6 i09861s1700
33,3 i10668s1049
34,0 FN692715-670
34,2 CT576062-1276 CT576062-272
36,2 F51TW9001B2Z8O-1201 F51TW9001B2Z8O-1116
BX251825-986
36,7 CT574752-818
38,1 CT577173-74
43,3 CR393317-921
50,9 i12813s393
51,4 CT582977-1210 BX253774-816
BX253774-54
57,6 F51TW9002J1WGY-647
60,7 F51TW9001B2AXP-340
61,1 AL750591-840
66,3 BX252221-1003 BX252221-182
68,4 F51TW9001AI55E-2114
69,8 CT577081-1293
71,9 BX254728-1302
72,4 BX254728-891
73,4 BX249621-1066
74,5 i10701s357
74,6 i10701s651
75,2 AL751301-752
75,8 BX254586-1299 m913
76,2 F51TW9002G7LOJ-544
76,4 F51TW9001CPZSV-706
76,5 AL749783-667
76,6 CT577574-1073
77,4 F5V9AAZ01BLAVC-89
80,2 i11115s82
80,5 i11115s146
85,7 m492
86,0 i11754s953
86,8 i11535s678
87,7 i13590s208
87,8 i13590s46
88,8 i10594s535
99,6 FN695400-1167 BX248867-129
101,0 BX253714-172
101,1 BX253714-228
103,4 F51TW9001DHJG8-1242
103,5 F51TW9001DHJG8-1311
104,5 i13311s398 i13217s887
105,8 E40-CATA_184
116,1 F51TW9001ALR0F-1291
116,7 BX251930-1172 BX251930-1223
117,8 F51TW9002J4AQY-74
118,1 F51TW9001AM6TF-1079 CT578868-1132
118,3 m170
118,9 F51TW9001B8Q0H-121 BX251150-9
F51TW9001AM6TF-779
119,1 F51TW9001A1O3U-1285 BX681302-1512
F51TW9001A0TXI-342
119,3 i13939s475
119,4 i13939s680
120,5 i12989s409
121,4 i15525s351
121,9 i12469s328_gras
122,9 AL749591-1543
124,3 i09979s949
125,2 i12202s964
133,4 BX679165-1056
137,2 BX249015-289
138,2 BX784129-2495
145,2 F51TW9001D7H8P-765
150,2 F51TW9001CTLDD-627
150,9 BX250815-1158
151,1 BX250815-804
0,0 i13252s160
0,1 i10850s1207
0,2 i11998s204 i11998s456
0,9 i11853s995
2,6 i13845s830
3,1 i13845s652
3,2 CT575028-207 CT575028-371
8,4 CT575141-99
8,5 CT575141-341
10,0 BX678934-790
10,5 BX678934-481
13,8 i11013s188
13,9 i14166s363
14,3 BX251190-622
16,6 i08906s326
17,2 BX252347-1190 BX252347-1006
18,3 BX251190-660
21,7 BX251719-1055
22,0 BX251719-1586 i11945s890
23,1 BX679585-950
24,6 384_Dhn2-264
25,0 m594 m716
25,4 F51TW9001B73Q2-770
26,7 BX679786-834
27,8 i12452s585
28,2 i12452s1065
28,7 AL750061-830
31,4 CT576062-272 CT576062-1276
32,8 i10668s1049
33,4 FN692715-670
37,2 CT577173-74
42,7 CR393317-921
0,0 F5V9AAZ01BLAVC-89
0,5 F51TW9002G7LOJ-544
2,6 BX254728-891
2,7 BX254728-1302
5,0 BX254586-1299
5,3 m913
5,5 i11115s82
6,0 i11115s146
6,2 BX252221-1003 BX252221-182
6,4 AL750591-840
6,6 F51TW9001B2AXP-340
12,9 m492
13,9 i11535s678
15,1 FN695474-1274
15,8 i10594s535
30,3 E40-CATA_184
31,3 i13311s398 i13217s887
33,0 F51TW9001DHJG8-1242 F51TW9001DHJG8-1311
33,5 BX252245-679
43,5 i12202s964
44,2 i09979s949
44,9 i12989s409
45,4 i15525s351
46,1 i12469s328_gras
47,0 F51TW9001AM6TF-779 BX251150-9
F51TW9001B8Q0H-121 AL749591-1543
47,5 F51TW9002J4AQY-74
47,7 F51TW9001A1O3U-1285 BX681302-1512
F51TW9001A0TXI-342
47,8 BX251930-1223 BX251930-1172
48,0 m170
51,5 F51TW9001ALR0F-1291
62,3 BX679165-1056
66,1 BX249015-289
67,1 BX784129-2495
74,1 F51TW9001D7H8P-765
78,8 F51TW9001CTLDD-627
79,9 BX250815-1158
80,3 BX250815-804

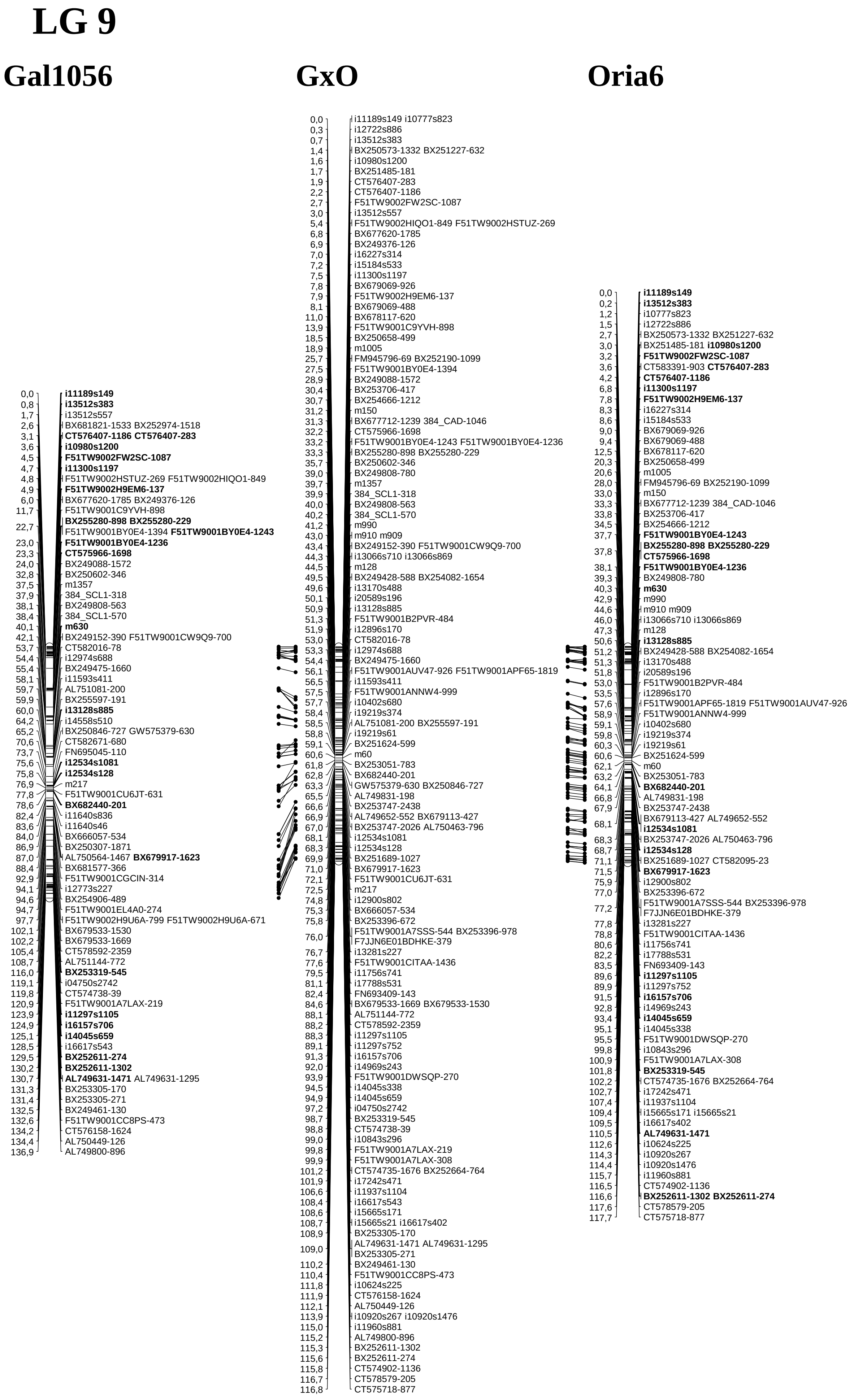

LG 9
Gal1056
GxO
Oria6
Gal1056:
0,0 i11189s149
0,8 i13512s383
1,7 i13512s557
2,6 BX681821-1533 BX252974-1518
3,1 CT576407-1186 CT576407-283
3,6 i10980s1200
4,5 F51TW9002FW2SC-1087
4,7 i11300s1197
4,8 F51TW9002HSTUZ-269 F51TW9002HIQO1-849
4,9 F51TW9002H9EM6-137
6,0 BX677620-1785 BX249376-126
11,7 F51TW9001C9YVH-898
BX255280-898 BX255280-229
22,7 F51TW9001BY0E4-1394 F51TW9001BY0E4-1243
23,0 F51TW9001BY0E4-1236
23,3 CT575966-1698
24,0 BX249088-1572
32,8 BX250602-346
37,5 m1357
37,9 384_SCL1-318
38,1 BX249808-563
38,4 384_SCL1-570
40,1 m630
42,1 BX249152-390 F51TW9001CW9Q9-700
53,7 CT582016-78
54,4 i12974s688
55,4 BX249475-1660
58,1 i11593s411
59,7 AL751081-200
59,9 BX255597-191
60,0 i13128s885
64,2 i14558s510
65,2 BX250846-727 GW575379-630
70,6 CT582671-680
73,7 FN695045-110
75,6 i12534s1081
75,8 i12534s128
76,9 m217
77,8 F51TW9001CU6JT-631
78,6 BX682440-201
82,4 i11640s836
83,6 i11640s46
84,0 BX666057-534
86,9 BX250307-1871
87,0 AL750564-1467 BX679917-1623
88,4 BX681577-366
92,9 F51TW9001CGCIN-314
94,1 i12773s227
94,6 BX254906-489
94,7 F51TW9001EL4A0-274
97,7 F51TW9002H9U6A-799 F51TW9002H9U6A-671
102,1 BX679533-1530
102,2 BX679533-1669
105,4 CT578592-2359
108,7 AL751144-772
116,0 BX253319-545
119,1 i04750s2742
119,8 CT574738-39
120,9 F51TW9001A7LAX-219
123,9 i11297s1105
124,9 i16157s706
125,1 i14045s659
128,5 i16617s543
129,5 BX252611-274
130,2 BX252611-1302
130,7 AL749631-1471 AL749631-1295
131,3 BX253305-170
131,4 BX253305-271
132,5 BX249461-130
132,6 F51TW9001CC8PS-473
134,2 CT576158-1624
134,4 AL750449-126
136,9 AL749800-896
GxO:
0,0 i11189s149 i10777s823
0,3 i12722s886
0,7 i13512s383
1,4 BX250573-1332 BX251227-632
1,6 i10980s1200
1,7 BX251485-181
1,9 CT576407-283
2,2 CT576407-1186
2,7 F51TW9002FW2SC-1087
3,0 i13512s557
5,4 F51TW9002HIQO1-849 F51TW9002HSTUZ-269
6,8 BX677620-1785
6,9 BX249376-126
7,0 i16227s314
7,2 i15184s533
7,5 i11300s1197
7,8 BX679069-926
7,9 F51TW9002H9EM6-137
8,1 BX679069-488
11,0 BX678117-620
13,9 F51TW9001C9YVH-898
18,5 BX250658-499
18,9 m1005
25,7 FM945796-69 BX252190-1099
27,5 F51TW9001BY0E4-1394
28,9 BX249088-1572
30,4 BX253706-417
30,7 BX254666-1212
31,2 m150
31,3 BX677712-1239 384_CAD-1046
32,2 CT575966-1698
33,2 F51TW9001BY0E4-1243 F51TW9001BY0E4-1236
33,3 BX255280-898 BX255280-229
35,7 BX250602-346
39,0 BX249808-780
39,7 m1357
39,9 384_SCL1-318
40,0 BX249808-563
40,2 384_SCL1-570
41,2 m990
43,0 m910 m909
43,4 BX249152-390 F51TW9001CW9Q9-700
44,3 i13066s710 i13066s869
44,5 m128
49,5 BX249428-588 BX254082-1654
49,6 i13170s488
50,1 i20589s196
50,9 i13128s885
51,3 F51TW9001B2PVR-484
51,9 i12896s170
53,0 CT582016-78
53,3 i12974s688
54,4 BX249475-1660
56,1 F51TW9001AUV47-926 F51TW9001APF65-1819
56,5 i11593s411
57,5 F51TW9001ANNW4-999
57,7 i10402s680
58,4 i19219s374
58,5 AL751081-200 BX255597-191
58,8 i19219s61
59,1 BX251624-599
60,6 m60
61,8 BX253051-783
62,8 BX682440-201
63,3 GW575379-630 BX250846-727
65,5 AL749831-198
66,6 BX253747-2438
66,9 AL749652-552 BX679113-427
67,0 BX253747-2026 AL750463-796
68,1 i12534s1081
68,3 i12534s128
69,9 BX251689-1027
71,0 BX679917-1623
72,1 F51TW9001CU6JT-631
72,5 m217
74,8 i12900s802
75,3 BX666057-534
75,8 BX253396-672
F51TW9001A7SSS-544 BX253396-978
76,0 F7JJN6E01BDHKE-379
76,7 i13281s227
77,6 F51TW9001CITAA-1436
79,5 i11756s741
81,1 i17788s531
82,4 FN693409-143
84,6 BX679533-1669 BX679533-1530
88,1 AL751144-772
88,2 CT578592-2359
88,3 i11297s1105
89,1 i11297s752
91,3 i16157s706
92,0 i14969s243
93,9 F51TW9001DWSQP-270
94,5 i14045s338
94,9 i14045s659
97,2 i04750s2742
98,7 BX253319-545
98,8 CT574738-39
99,0 i10843s296
99,8 F51TW9001A7LAX-219
99,9 F51TW9001A7LAX-308
101,2 CT574735-1676 BX252664-764
101,9 i17242s471
106,6 i11937s1104
108,4 i16617s543
108,6 i15665s171
108,7 i15665s21 i16617s402
108,9 BX253305-170
AL749631-1471 AL749631-1295
109,0 BX253305-271
110,2 BX249461-130
110,4 F51TW9001CC8PS-473
111,8 i10624s225
111,9 CT576158-1624
112,1 AL750449-126
113,9 i10920s267 i10920s1476
115,0 i11960s881
115,2 AL749800-896
115,3 BX252611-1302
115,6 BX252611-274
115,8 CT574902-1136
116,7 CT578579-205
116,8 CT575718-877
Oria6:
0,0 i11189s149
0,2 i13512s383
1,2 i10777s823
1,5 i12722s886
2,7 BX250573-1332 BX251227-632
3,0 BX251485-181 i10980s1200
3,2 F51TW9002FW2SC-1087
3,6 CT583391-903 CT576407-283
4,2 CT576407-1186
6,8 i11300s1197
7,8 F51TW9002H9EM6-137
8,3 i16227s314
8,6 i15184s533
9,0 BX679069-926
9,4 BX679069-488
12,5 BX678117-620
20,3 BX250658-499
20,6 m1005
28,0 FM945796-69 BX252190-1099
33,0 m150
33,3 BX677712-1239 384_CAD-1046
33,8 BX253706-417
34,5 BX254666-1212
37,7 F51TW9001BY0E4-1243
BX255280-898 BX255280-229
37,8 CT575966-1698
38,1 F51TW9001BY0E4-1236
39,3 BX249808-780
40,3 m630
42,9 m990
44,6 m910 m909
46,0 i13066s710 i13066s869
47,3 m128
50,6 i13128s885
51,2 BX249428-588 BX254082-1654
51,3 i13170s488
51,8 i20589s196
53,0 F51TW9001B2PVR-484
53,5 i12896s170
57,6 F51TW9001APF65-1819 F51TW9001AUV47-926
58,9 F51TW9001ANNW4-999
59,1 i10402s680
59,8 i19219s374
60,3 i19219s61
60,6 BX251624-599
62,1 m60
63,2 BX253051-783
64,1 BX682440-201
66,8 AL749831-198
67,9 BX253747-2438
BX679113-427 AL749652-552
68,1 i12534s1081
68,3 BX253747-2026 AL750463-796
68,7 i12534s128
71,1 BX251689-1027 CT582095-23
71,5 BX679917-1623
75,9 i12900s802
77,0 BX253396-672
F51TW9001A7SSS-544 BX253396-978
77,2 F7JJN6E01BDHKE-379
77,8 i13281s227
78,8 F51TW9001CITAA-1436
80,6 i11756s741
82,2 i17788s531
83,5 FN693409-143
89,6 i11297s1105
89,9 i11297s752
91,5 i16157s706
92,8 i14969s243
93,4 i14045s659
95,1 i14045s338
95,5 F51TW9001DWSQP-270
99,8 i10843s296
100,9 F51TW9001A7LAX-308
101,8 BX253319-545
102,2 CT574735-1676 BX252664-764
102,7 i17242s471
107,4 i11937s1104
109,4 i15665s171 i15665s21
109,5 i16617s402
110,5 AL749631-1471
112,6 i10624s225
114,3 i10920s267
114,4 i10920s1476
115,7 i11960s881
116,5 CT574902-1136
116,6 BX252611-1302 BX252611-274
117,6 CT578579-205
117,7 CT575718-877

LG 10

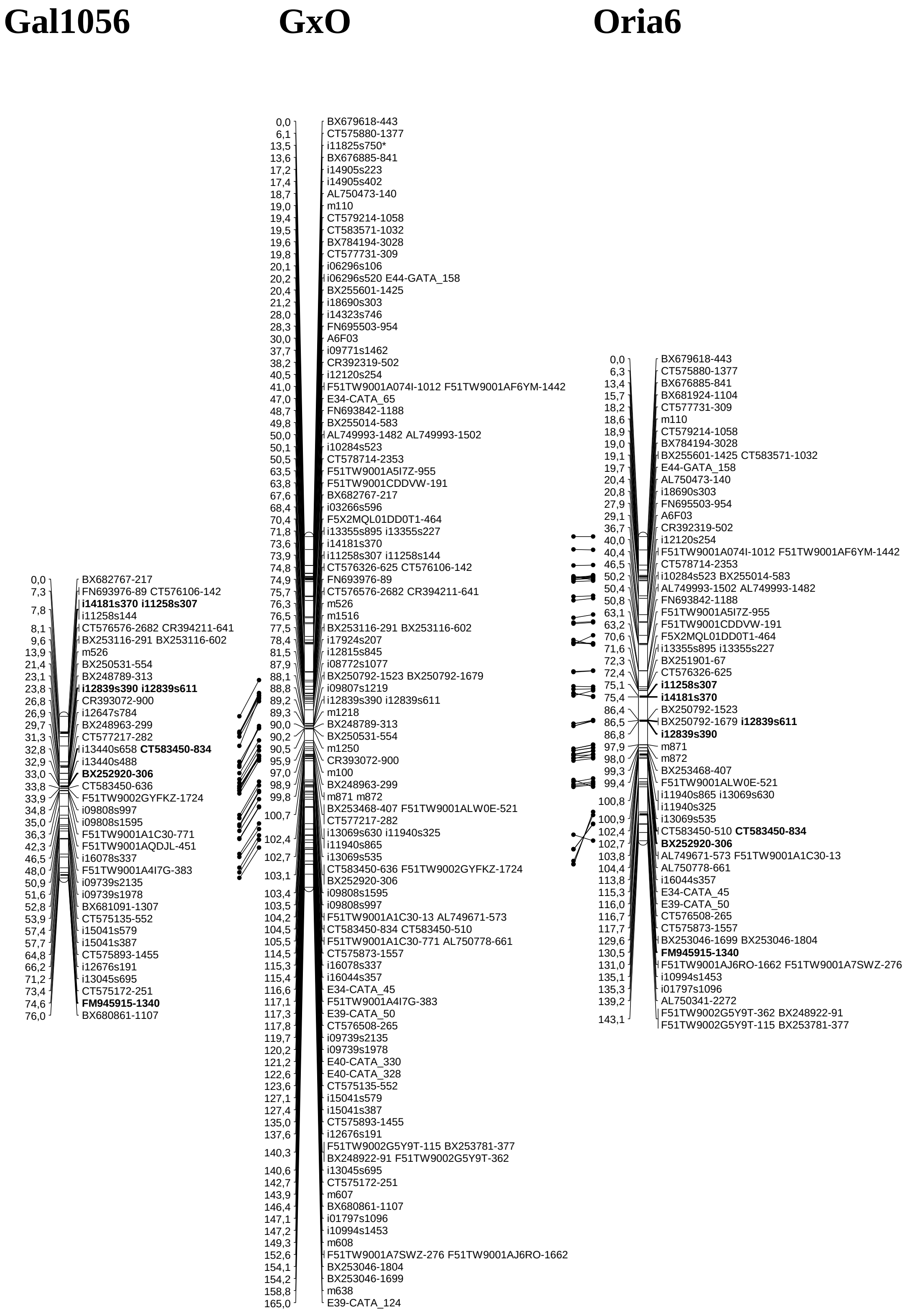
Gal1056
0,0 BX682767-217
7,3 FN693976-89 CT576106-142
7,8 i14181s370 i11258s307 / i11258s144
8,1 CT576576-2682 CR394211-641
9,6 BX253116-291 BX253116-602
13,9 m526
21,4 BX250531-554
23,1 BX248789-313
23,8 i12839s390 i12839s611
26,8 CR393072-900
26,9 i12647s784
29,7 BX248963-299
31,3 CT577217-282
32,8 i13440s658 CT583450-834
32,9 i13440s488
33,0 BX252920-306
33,8 CT583450-636
33,9 F51TW9002GYFKZ-1724
34,8 i09808s997
35,0 i09808s1595
36,3 F51TW9001A1C30-771
42,3 F51TW9001AQDJL-451
46,5 i16078s337
48,0 F51TW9001A4I7G-383
50,9 i09739s2135
51,6 i09739s1978
52,8 BX681091-1307
53,9 CT575135-552
57,4 i15041s579
57,7 i15041s387
64,8 CT575893-1455
66,2 i12676s191
71,2 i13045s695
73,4 CT575172-251
74,6 FM945915-1340
76,0 BX680861-1107
GxO
0,0 BX679618-443
6,1 CT575880-1377
13,5 i11825s750*
13,6 BX676885-841
17,2 i14905s223
17,4 i14905s402
18,7 AL750473-140
19,0 m110
19,4 CT579214-1058
19,5 CT583571-1032
19,6 BX784194-3028
19,8 CT577731-309
20,1 i06296s106
20,2 i06296s520 E44-GATA_158
20,4 BX255601-1425
21,2 i18690s303
28,0 i14323s746
28,3 FN695503-954
30,0 A6F03
37,7 i09771s1462
38,2 CR392319-502
40,5 i12120s254
41,0 F51TW9001A074I-1012 F51TW9001AF6YM-1442
47,0 E34-CATA_65
48,7 FN693842-1188
49,8 BX255014-583
50,0 AL749993-1482 AL749993-1502
50,1 i10284s523
50,5 CT578714-2353
63,5 F51TW9001A5I7Z-955
63,8 F51TW9001CDDVW-191
67,6 BX682767-217
68,4 i03266s596
70,4 F5X2MQL01DD0T1-464
71,8 i13355s895 i13355s227
73,6 i14181s370
73,9 i11258s307 i11258s144
74,8 CT576326-625 CT576106-142
74,9 FN693976-89
75,7 CT576576-2682 CR394211-641
76,3 m526
76,5 m1516
77,5 BX253116-291 BX253116-602
78,4 i17924s207
81,5 i12815s845
87,9 i08772s1077
88,1 BX250792-1523 BX250792-1679
88,8 i09807s1219
89,2 i12839s390 i12839s611
89,3 m1218
90,0 BX248789-313
90,2 BX250531-554
90,5 m1250
95,9 CR393072-900
97,0 m100
98,9 BX248963-299
99,8 m871 m872
100,7 BX253468-407 F51TW9001ALW0E-521 / CT577217-282
102,4 i13069s630 i11940s325 / i11940s865
102,7 i13069s535
103,1 CT583450-636 F51TW9002GYFKZ-1724 / BX252920-306
103,4 i09808s1595
103,5 i09808s997
104,2 F51TW9001A1C30-13 AL749671-573
104,5 CT583450-834 CT583450-510
105,5 F51TW9001A1C30-771 AL750778-661
114,5 CT575873-1557
115,3 i16078s337
115,4 i16044s357
116,6 E34-CATA_45
117,1 F51TW9001A4I7G-383
117,3 E39-CATA_50
117,8 CT576508-265
119,7 i09739s2135
120,2 i09739s1978
121,2 E40-CATA_330
122,6 E40-CATA_328
123,6 CT575135-552
127,1 i15041s579
127,4 i15041s387
135,0 CT575893-1455
137,6 i12676s191
140,3 F51TW9002G5Y9T-115 BX253781-377 / BX248922-91 F51TW9002G5Y9T-362
140,6 i13045s695
142,7 CT575172-251
143,9 m607
146,4 BX680861-1107
147,1 i01797s1096
147,2 i10994s1453
149,3 m608
152,6 F51TW9001A7SWZ-276 F51TW9001AJ6RO-1662
154,1 BX253046-1804
154,2 BX253046-1699
158,8 m638
165,0 E39-CATA_124
Oria6
0,0 BX679618-443
6,3 CT575880-1377
13,4 BX676885-841
15,7 BX681924-1104
18,2 CT577731-309
18,6 m110
18,9 CT579214-1058
19,0 BX784194-3028
19,1 BX255601-1425 CT583571-1032
19,7 E44-GATA_158
20,4 AL750473-140
20,8 i18690s303
27,9 FN695503-954
29,1 A6F03
36,7 CR392319-502
40,0 i12120s254
40,4 F51TW9001A074I-1012 F51TW9001AF6YM-1442
46,5 CT578714-2353
50,2 i10284s523 BX255014-583
50,4 AL749993-1502 AL749993-1482
50,8 FN693842-1188
63,1 F51TW9001A5I7Z-955
63,2 F51TW9001CDDVW-191
70,6 F5X2MQL01DD0T1-464
71,6 i13355s895 i13355s227
72,3 BX251901-67
72,4 CT576326-625
75,1 i11258s307
75,4 i14181s370
86,4 BX250792-1523
86,5 BX250792-1679 i12839s611
86,8 i12839s390
97,9 m871
98,0 m872
99,3 BX253468-407
99,4 F51TW9001ALW0E-521
100,8 i11940s865 i13069s630 / i11940s325
100,9 i13069s535
102,4 CT583450-510 CT583450-834
102,7 BX252920-306
103,8 AL749671-573 F51TW9001A1C30-13
104,4 AL750778-661
113,8 i16044s357
115,3 E34-CATA_45
116,0 E39-CATA_50
116,7 CT576508-265
117,7 CT575873-1557
129,6 BX253046-1699 BX253046-1804
130,5 FM945915-1340
131,0 F51TW9001AJ6RO-1662 F51TW9001A7SWZ-276
135,1 i10994s1453
135,3 i01797s1096
139,2 AL750341-2272
143,1 F51TW9002G5Y9T-362 BX248922-91 / F51TW9002G5Y9T-115 BX253781-377

LG 11

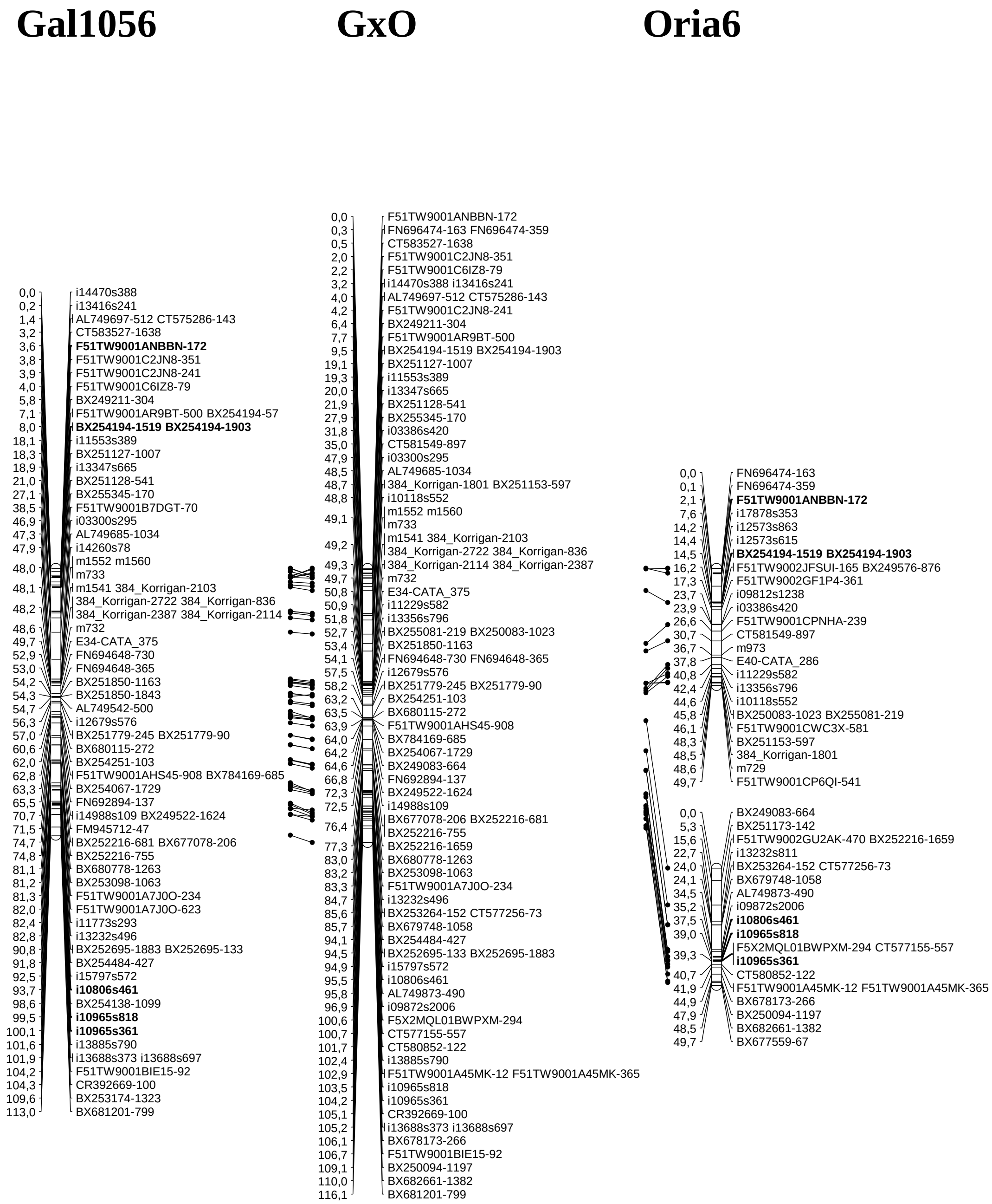

Gal1056
GxO
Oria6
0,0 i14470s388
0,2 i13416s241
1,4 AL749697-512 CT575286-143
3,2 CT583527-1638
3,6 F51TW9001ANBBN-172
3,8 F51TW9001C2JN8-351
3,9 F51TW9001C2JN8-241
4,0 F51TW9001C6IZ8-79
5,8 BX249211-304
7,1 F51TW9001AR9BT-500 BX254194-57
8,0 BX254194-1519 BX254194-1903
18,1 i11553s389
18,3 BX251127-1007
18,9 i13347s665
21,0 BX251128-541
27,1 BX255345-170
38,5 F51TW9001B7DGT-70
46,9 i03300s295
47,3 AL749685-1034
47,9 i14260s78
48,0 m1552 m1560
m733
48,1 m1541 384_Korrigan-2103
384_Korrigan-2722 384_Korrigan-836
48,2 384_Korrigan-2387 384_Korrigan-2114
48,6 m732
49,7 E34-CATA_375
52,9 FN694648-730
53,0 FN694648-365
54,2 BX251850-1163
54,3 BX251850-1843
54,7 AL749542-500
56,3 i12679s576
57,0 BX251779-245 BX251779-90
60,6 BX680115-272
62,0 BX254251-103
62,8 F51TW9001AHS45-908 BX784169-685
63,3 BX254067-1729
65,5 FN692894-137
70,7 i14988s109 BX249522-1624
71,5 FM945712-47
74,7 BX252216-681 BX677078-206
74,8 BX252216-755
81,1 BX680778-1263
81,2 BX253098-1063
81,3 F51TW9001A7J0O-234
82,0 F51TW9001A7J0O-623
82,4 i11773s293
82,8 i13232s496
90,8 BX252695-1883 BX252695-133
91,8 BX254484-427
92,5 i15797s572
93,7 i10806s461
98,6 BX254138-1099
99,5 i10965s818
100,1 i10965s361
101,6 i13885s790
101,9 i13688s373 i13688s697
104,2 F51TW9001BIE15-92
104,3 CR392669-100
109,6 BX253174-1323
113,0 BX681201-799
0,0 F51TW9001ANBBN-172
0,3 FN696474-163 FN696474-359
0,5 CT583527-1638
2,0 F51TW9001C2JN8-351
2,2 F51TW9001C6IZ8-79
3,2 i14470s388 i13416s241
4,0 AL749697-512 CT575286-143
4,2 F51TW9001C2JN8-241
6,4 BX249211-304
7,7 F51TW9001AR9BT-500
9,5 BX254194-1519 BX254194-1903
19,1 BX251127-1007
19,3 i11553s389
20,0 i13347s665
21,9 BX251128-541
27,9 BX255345-170
31,8 i03386s420
35,0 CT581549-897
47,9 i03300s295
48,5 AL749685-1034
48,7 384_Korrigan-1801 BX251153-597
48,8 i10118s552
49,1 m1552 m1560
m733
49,2 m1541 384_Korrigan-2103
384_Korrigan-2722 384_Korrigan-836
49,3 384_Korrigan-2114 384_Korrigan-2387
49,7 m732
50,8 E34-CATA_375
50,9 i11229s582
51,8 i13356s796
52,7 BX255081-219 BX250083-1023
53,4 BX251850-1163
54,1 FN694648-730 FN694648-365
57,5 i12679s576
58,2 BX251779-245 BX251779-90
63,2 BX254251-103
63,5 BX680115-272
63,9 F51TW9001AHS45-908
64,0 BX784169-685
64,2 BX254067-1729
64,6 BX249083-664
66,8 FN692894-137
72,3 BX249522-1624
72,5 i14988s109
76,4 BX677078-206 BX252216-681
BX252216-755
77,3 BX252216-1659
83,0 BX680778-1263
83,2 BX253098-1063
83,3 F51TW9001A7J0O-234
84,7 i13232s496
85,6 BX253264-152 CT577256-73
85,7 BX679748-1058
94,1 BX254484-427
94,5 BX252695-133 BX252695-1883
94,9 i15797s572
95,5 i10806s461
95,8 AL749873-490
96,9 i09872s2006
100,6 F5X2MQL01BWPXM-294
100,7 CT577155-557
101,7 CT580852-122
102,4 i13885s790
102,9 F51TW9001A45MK-12 F51TW9001A45MK-365
103,5 i10965s818
104,2 i10965s361
105,1 CR392669-100
105,2 i13688s373 i13688s697
106,1 BX678173-266
106,7 F51TW9001BIE15-92
109,1 BX250094-1197
110,0 BX682661-1382
116,1 BX681201-799
0,0 FN696474-163
0,1 FN696474-359
2,1 F51TW9001ANBBN-172
7,6 i17878s353
14,2 i12573s863
14,4 i12573s615
14,5 BX254194-1519 BX254194-1903
16,2 F51TW9002JFSUI-165 BX249576-876
17,3 F51TW9002GF1P4-361
23,7 i09812s1238
23,9 i03386s420
26,6 F51TW9001CPNHA-239
30,7 CT581549-897
36,7 m973
37,8 E40-CATA_286
40,8 i11229s582
42,4 i13356s796
44,6 i10118s552
45,8 BX250083-1023 BX255081-219
46,1 F51TW9001CWC3X-581
48,3 BX251153-597
48,5 384_Korrigan-1801
48,6 m729
49,7 F51TW9001CP6QI-541
0,0 BX249083-664
5,3 BX251173-142
15,6 F51TW9002GU2AK-470 BX252216-1659
22,7 i13232s811
24,0 BX253264-152 CT577256-73
24,1 BX679748-1058
34,5 AL749873-490
35,2 i09872s2006
37,5 i10806s461
39,0 i10965s818
39,3 F5X2MQL01BWPXM-294 CT577155-557
i10965s361
40,7 CT580852-122
41,9 F51TW9001A45MK-12 F51TW9001A45MK-365
44,9 BX678173-266
47,9 BX250094-1197
48,5 BX682661-1382
49,7 BX677559-67

LG 12

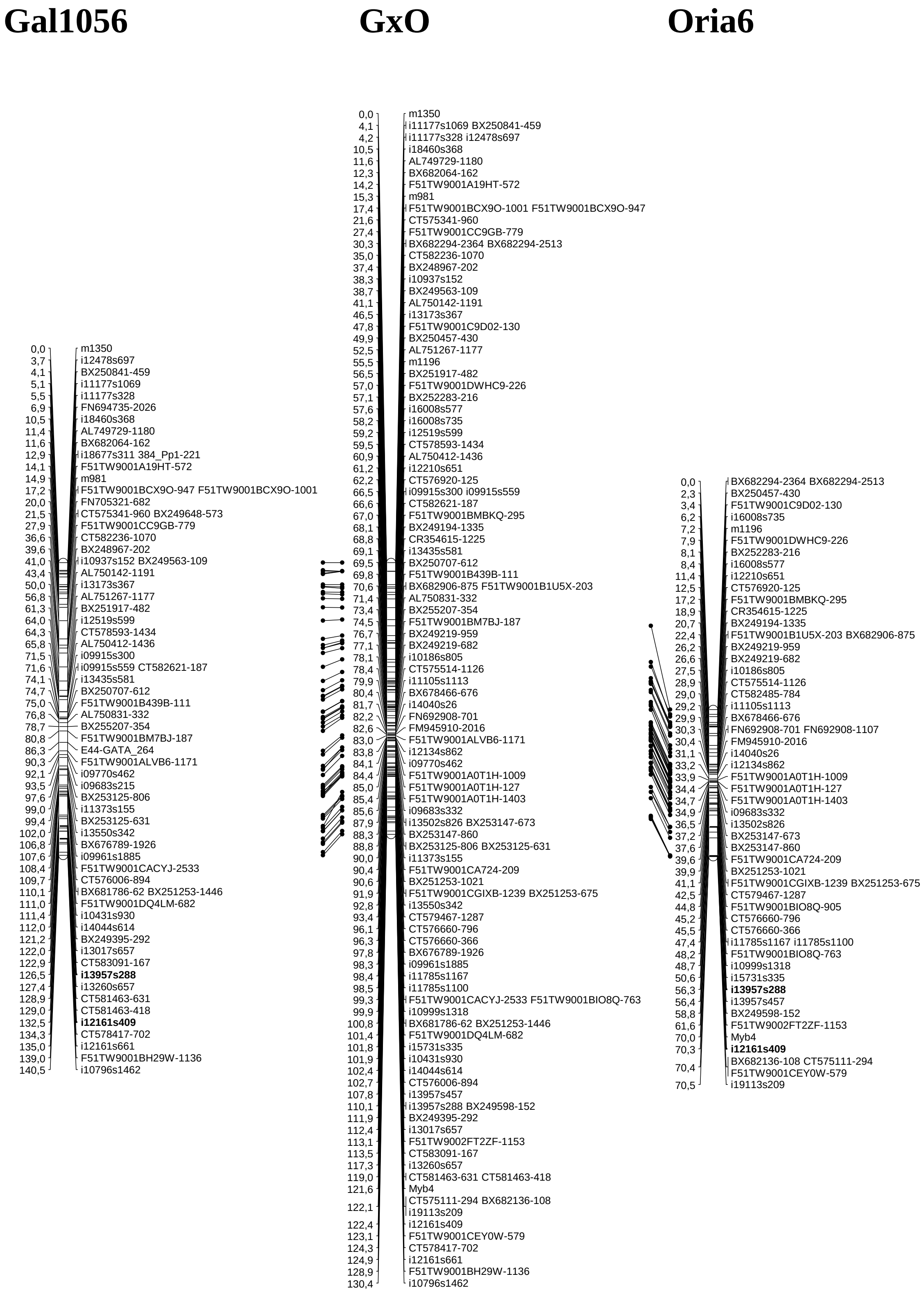
Gal1056
0,0 m1350
3,7 i12478s697
4,1 BX250841-459
5,1 i11177s1069
5,5 i11177s328
6,9 FN694735-2026
10,5 i18460s368
11,4 AL749729-1180
11,6 BX682064-162
12,9 i18677s311 384_Pp1-221
14,1 F51TW9001A19HT-572
14,9 m981
17,2 F51TW9001BCX9O-947 F51TW9001BCX9O-1001
20,0 FN705321-682
21,5 CT575341-960 BX249648-573
27,9 F51TW9001CC9GB-779
36,6 CT582236-1070
39,6 BX248967-202
41,0 i10937s152 BX249563-109
43,4 AL750142-1191
50,0 i13173s367
56,8 AL751267-1177
61,3 BX251917-482
64,0 i12519s599
64,3 CT578593-1434
65,8 AL750412-1436
71,5 i09915s300
71,6 i09915s559 CT582621-187
74,1 i13435s581
74,7 BX250707-612
75,0 F51TW9001B439B-111
76,8 AL750831-332
78,7 BX255207-354
80,8 F51TW9001BM7BJ-187
86,3 E44-GATA_264
90,3 F51TW9001ALVB6-1171
92,1 i09770s462
93,5 i09683s215
97,6 BX253125-806
99,0 i11373s155
99,4 BX253125-631
102,0 i13550s342
106,8 BX676789-1926
107,6 i09961s1885
108,4 F51TW9001CACYJ-2533
109,7 CT576006-894
110,1 BX681786-62 BX251253-1446
111,0 F51TW9001DQ4LM-682
111,4 i10431s930
112,0 i14044s614
121,2 BX249395-292
122,0 i13017s657
122,9 CT583091-167
126,5 i13957s288
127,4 i13260s657
128,9 CT581463-631
129,0 CT581463-418
132,5 i12161s409
134,3 CT578417-702
135,0 i12161s661
139,0 F51TW9001BH29W-1136
140,5 i10796s1462
GxO
0,0 m1350
4,1 i11177s1069 BX250841-459
4,2 i11177s328 i12478s697
10,5 i18460s368
11,6 AL749729-1180
12,3 BX682064-162
14,2 F51TW9001A19HT-572
15,3 m981
17,4 F51TW9001BCX9O-1001 F51TW9001BCX9O-947
21,6 CT575341-960
27,4 F51TW9001CC9GB-779
30,3 BX682294-2364 BX682294-2513
35,0 CT582236-1070
37,4 BX248967-202
38,3 i10937s152
38,7 BX249563-109
41,1 AL750142-1191
46,5 i13173s367
47,8 F51TW9001C9D02-130
49,9 BX250457-430
52,5 AL751267-1177
55,5 m1196
56,5 BX251917-482
57,0 F51TW9001DWHC9-226
57,1 BX252283-216
57,6 i16008s577
58,2 i16008s735
59,2 i12519s599
59,5 CT578593-1434
60,9 AL750412-1436
61,2 i12210s651
62,2 CT576920-125
66,5 i09915s300 i09915s559
66,6 CT582621-187
67,0 F51TW9001BMBKQ-295
68,1 BX249194-1335
68,8 CR354615-1225
69,1 i13435s581
69,5 BX250707-612
69,8 F51TW9001B439B-111
70,6 BX682906-875 F51TW9001B1U5X-203
71,4 AL750831-332
73,4 BX255207-354
74,5 F51TW9001BM7BJ-187
76,7 BX249219-959
77,1 BX249219-682
78,1 i10186s805
78,4 CT575514-1126
79,9 i11105s1113
80,4 BX678466-676
81,7 i14040s26
82,2 FN692908-701
82,6 FM945910-2016
83,0 F51TW9001ALVB6-1171
83,8 i12134s862
84,1 i09770s462
84,4 F51TW9001A0T1H-1009
85,0 F51TW9001A0T1H-127
85,4 F51TW9001A0T1H-1403
85,6 i09683s332
87,9 i13502s826 BX253147-673
88,3 BX253147-860
88,8 BX253125-806 BX253125-631
90,0 i11373s155
90,4 F51TW9001CA724-209
90,6 BX251253-1021
91,9 F51TW9001CGIXB-1239 BX251253-675
92,8 i13550s342
93,4 CT579467-1287
96,1 CT576660-796
96,3 CT576660-366
97,8 BX676789-1926
98,3 i09961s1885
98,4 i11785s1167
98,5 i11785s1100
99,3 F51TW9001CACYJ-2533 F51TW9001BIO8Q-763
99,9 i10999s1318
100,8 BX681786-62 BX251253-1446
101,4 F51TW9001DQ4LM-682
101,8 i15731s335
101,9 i10431s930
102,4 i14044s614
102,7 CT576006-894
107,8 i13957s457
110,1 i13957s288 BX249598-152
111,9 BX249395-292
112,4 i13017s657
113,1 F51TW9002FT2ZF-1153
113,5 CT583091-167
117,3 i13260s657
119,0 CT581463-631 CT581463-418
121,6 Myb4
122,1 CT575111-294 BX682136-108
i19113s209
122,4 i12161s409
123,1 F51TW9001CEY0W-579
124,3 CT578417-702
124,9 i12161s661
128,9 F51TW9001BH29W-1136
130,4 i10796s1462
Oria6
0,0 BX682294-2364 BX682294-2513
2,3 BX250457-430
3,4 F51TW9001C9D02-130
6,2 i16008s735
7,2 m1196
7,9 F51TW9001DWHC9-226
8,1 BX252283-216
8,4 i16008s577
11,4 i12210s651
12,5 CT576920-125
17,2 F51TW9001BMBKQ-295
18,9 CR354615-1225
20,7 BX249194-1335
22,4 F51TW9001B1U5X-203 BX682906-875
26,2 BX249219-959
26,6 BX249219-682
27,5 i10186s805
28,9 CT575514-1126
29,0 CT582485-784
29,2 i11105s1113
29,9 BX678466-676
30,3 FN692908-701 FN692908-1107
30,4 FM945910-2016
31,1 i14040s26
33,2 i12134s862
33,9 F51TW9001A0T1H-1009
34,4 F51TW9001A0T1H-127
34,7 F51TW9001A0T1H-1403
34,9 i09683s332
36,5 i13502s826
37,2 BX253147-673
37,6 BX253147-860
39,6 F51TW9001CA724-209
39,9 BX251253-1021
41,1 F51TW9001CGIXB-1239 BX251253-675
42,5 CT579467-1287
44,8 F51TW9001BIO8Q-905
45,2 CT576660-796
45,5 CT576660-366
47,4 i11785s1167 i11785s1100
48,2 F51TW9001BIO8Q-763
48,7 i10999s1318
50,6 i15731s335
56,3 i13957s288
56,4 i13957s457
58,8 BX249598-152
61,6 F51TW9002FT2ZF-1153
70,0 Myb4
70,3 i12161s409
70,4 BX682136-108 CT575111-294
F51TW9001CEY0W-579
70,5 i19113s209
