# Supplementary material for: Genetic control of functional traits related to photosynthesis and water use efficiency in Pinus pinaster Ait. drought response: integration of genome annotation, allele association and QTL detection for candidate gene identification
Source: BMC Genomics. 2014 Jun 12;15(1):464. doi: 10.1186/1471-2164-15-464 (PMC4144121; doi:10.1186/1471-2164-15-464)
Supplement: Supplementary file 6 — Additional file 6: Marker order comparison with maps obtained by Chancerel et al. [20] . (PDF 63 KB) [file 12864_2013_6163_MOESM6_ESM.pdf]

Additional File 7. Marker order comparison between integrated map of the two progenitors of Gal 1056 x Oria 6 controlled cross obtained in this study (GxO) and framework markers positioned in Chancerel et al. [19] linkage maps (G2F, G2M and F2). Area between vertical or horizontal black lines represents the linkage group size for each of the analyzed maps. Red dots represent the non-syntenic markers of contigs FN696780, CT577280, AL749831 and BX249015 that are discussed in the text.

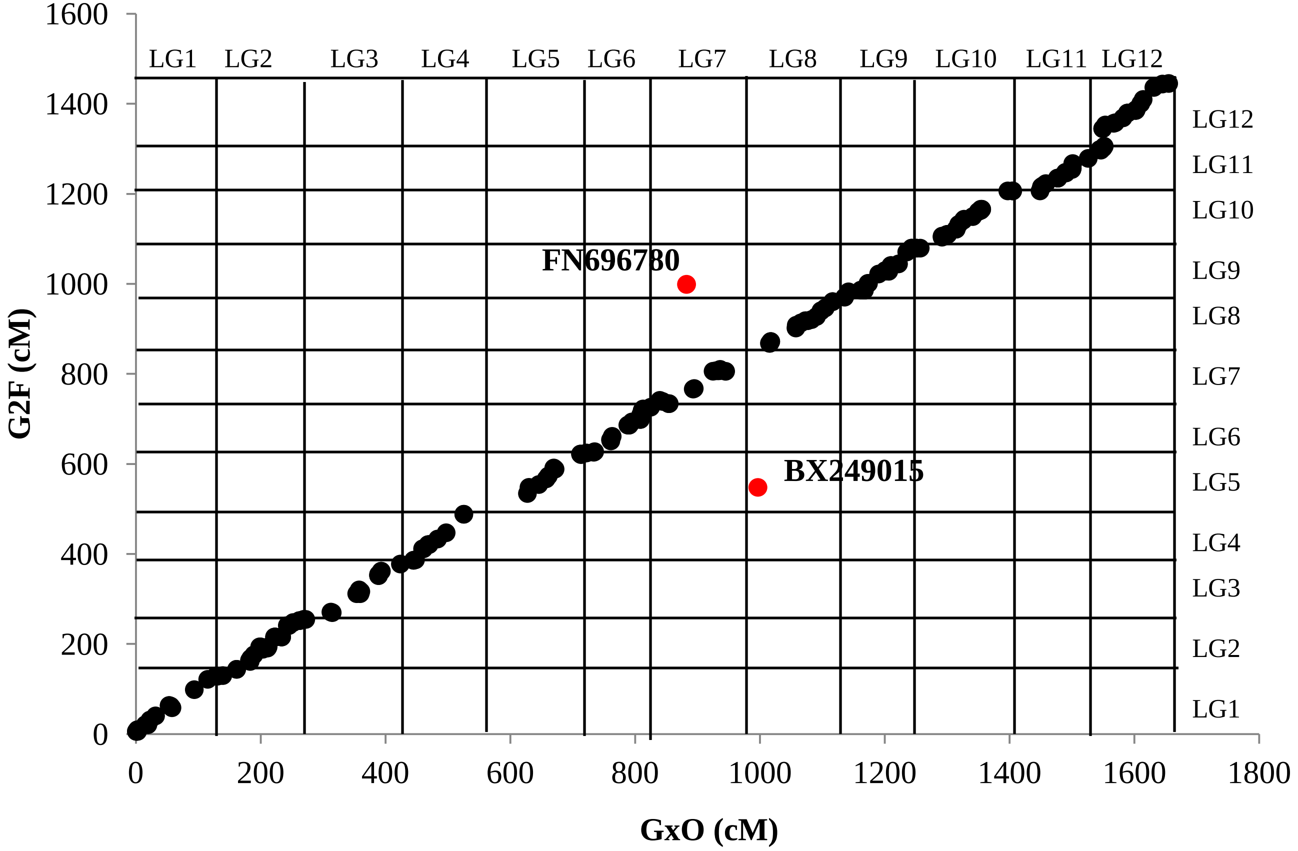

1600
1400
1200
1000
800
600
400
200
0
G2F (cM)
LG1
LG2
LG3
LG4
LG5
LG6
LG7
LG8
LG9
LG10
LG11
LG12
FN696780
BX249015
0
200
400
600
800
1000
1200
1400
1600
1800
GxO (cM)

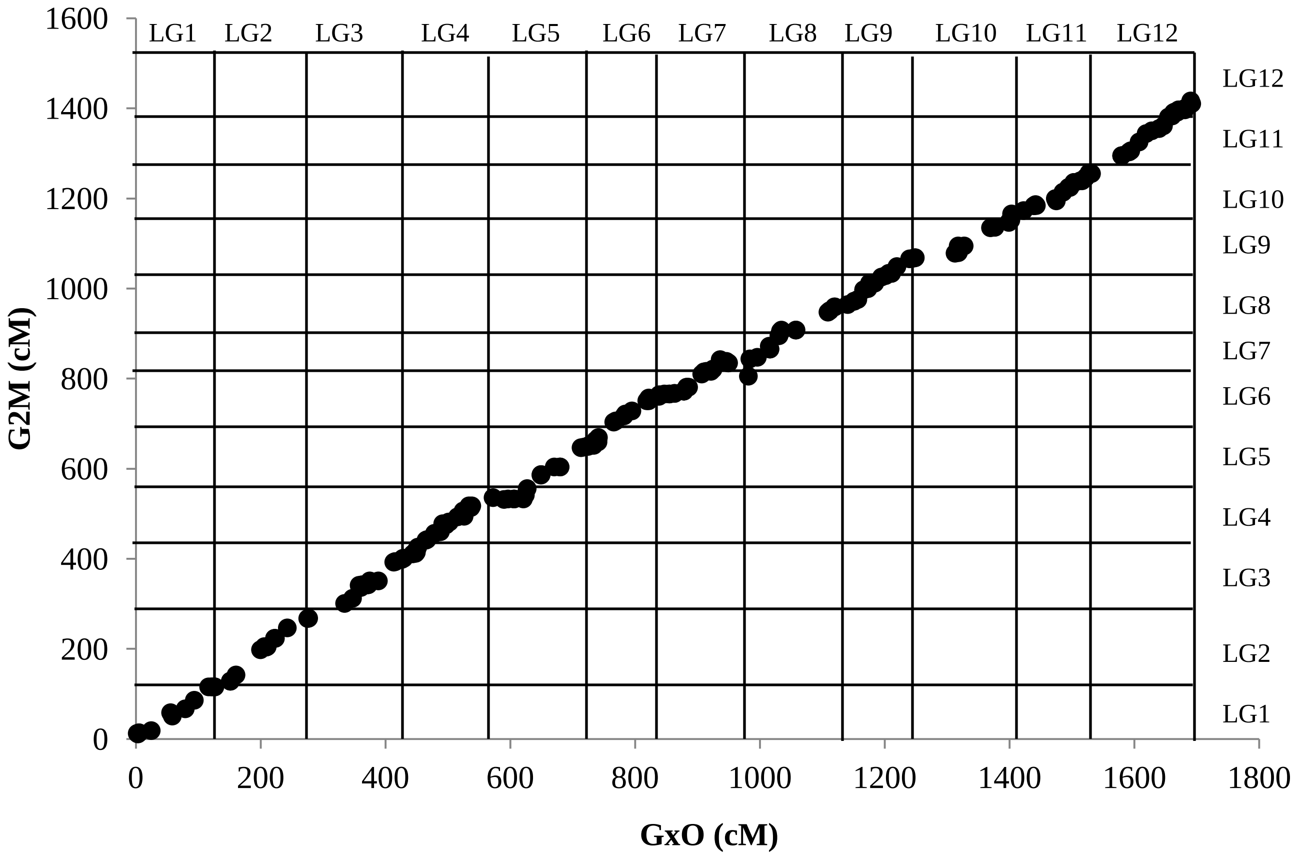
G2M (cM)
GxO (cM)
0
200
400
600
800
1000
1200
1400
1600
1800
LG1
LG2
LG3
LG4
LG5
LG6
LG7
LG8
LG9
LG10
LG11
LG12

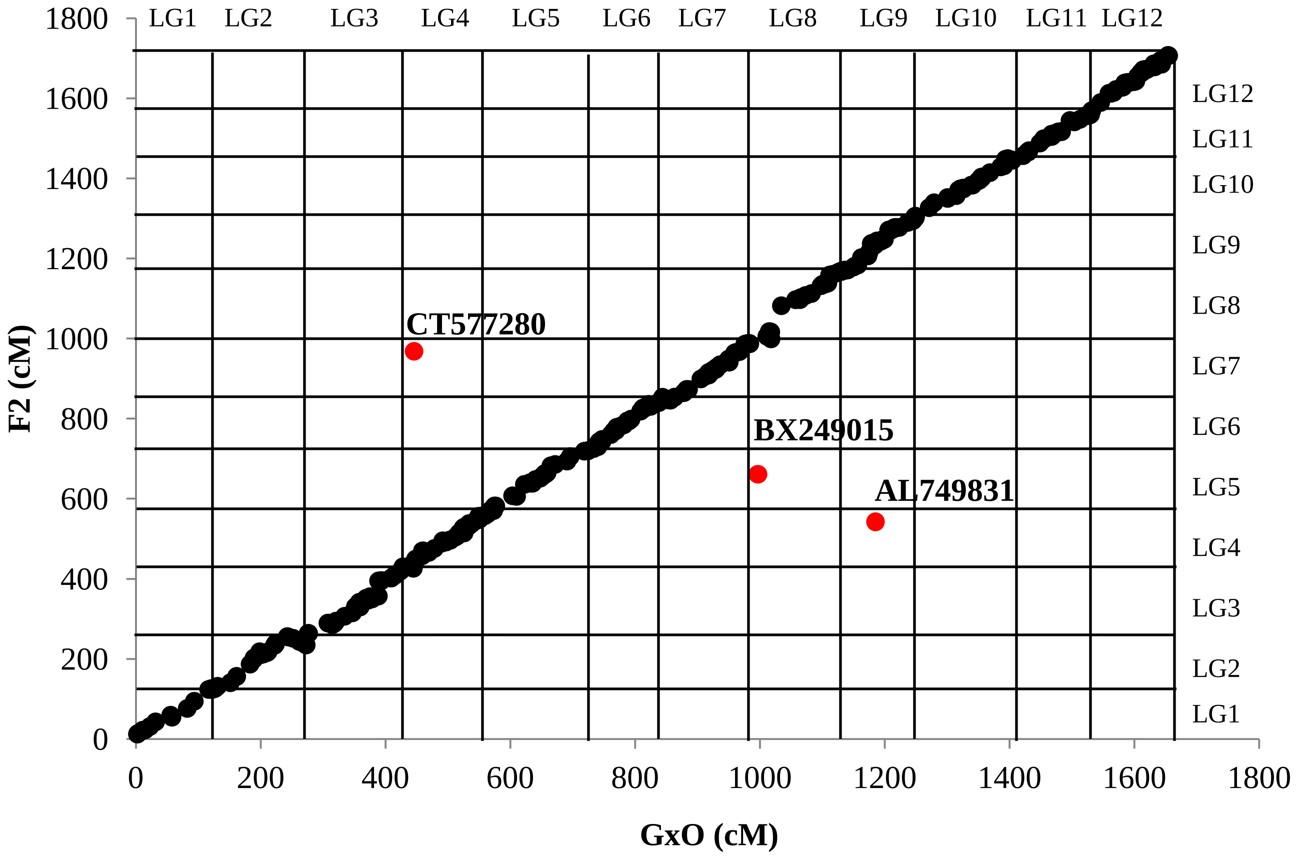
LG1
LG2
LG3
LG4
LG5
LG6
LG7
LG8
LG9
LG10
LG11
LG12
F2 (cM)
GxO (cM)
CT577280
BX249015
AL749831
0
200
400
600
800
1000
1200
1400
1600
1800
